# *Tribolium madens* satellitome reveals a network of highly abundant satellite DNAs in megabase-sized regions hallmarked by macro-dyad symmetries

**DOI:** 10.1186/s13059-026-04022-0

**Published:** 2026-03-07

**Authors:** Damira Veseljak, Evelin Despot-Slade, Marin Volarić, Lucija Horvat, Nevenka Meštrović, Brankica Mravinac

**Affiliations:** Division of Molecular Biology, Ruđer Bošković Institute, Bijenička cesta 54, Zagreb, 10000 Croatia

**Keywords:** Satellite DNA, Satellitome, Centromere, Pericentromeric regions, Macro-dyad symmetry, Non-B-form DNA, Satellite/microsatellite conjunction, Genome assembly, *Tribolium*

## Abstract

**Background:**

Tandemly repeated satellite DNAs (satDNAs) are among the most copious sequences of eukaryotic genomes. They often reside in centromeric regions, but their diversity among different organisms obscures the properties that centromere-competent satDNAs should possess.

**Results:**

Here, we explore the satellitome of the satDNA-rich flour beetle *Tribolium madens*. By combining short-read Illumina and long-read PacBio HiFi sequencing, we identify 124 satDNAs comprising 41.4% of the genome. We find that 38% of the genome sequence originates from a ~ 110 bp element that gives rise to two distinct satDNAs, the major and minor satellites, which occupy multi-megabase regions likely encompassing (peri)centromeres of all chromosomes. Fine-scale analysis of long-range organization reveals that intermingled arrays of the major and minor satDNAs are arranged in macro-dyad symmetries with the potential to form hairpin or cruciform structures spanning tens of kilobases. The inversion sites within macro-dyad symmetries and the transition zones between the major and minor satDNA arrays are highly conserved, indicating structural significance. The organization of the *Tribolium madens* putative (peri)centromeric satDNAs is comparable to that of the closely related *Tribolium freemani* and *Tribolium castaneum*, whose completely different dominant satDNAs also incline toward macro-dyad symmetries.

**Conclusions:**

We propose that satDNA-related macro-dyad symmetries may affect the organization of (peri)centromeric chromatin, potentially also influencing centromere specification. The analogous pattern in congeners suggests that such symmetries are an intrinsic feature of *Tribolium* (peri)centromeric regions, implying that repeat organization and potential non-canonical DNA structures could be functionally more significant than the primary sequence of satDNA repeats.

**Supplementary Information:**

The online version contains supplementary material available at 10.1186/s13059-026-04022-0.

## Background

Satellite DNAs (satDNAs) are tandemly repeated sequences that, together with transposable elements, dominate in eukaryotic genomes. Despite their abundance, the ubiquitous function of satDNAs is not yet clear. Among the various proposed roles, the most consistently observed is their presence in the centromeric regions of most animal and plant species [1, 2].

The remarkable diversity of centromeric DNAs, even among closely related species, rules out the existence of a universal centromere-competent satDNA. Consequently, independently of DNA sequence, functional centromeres are mostly defined by the presence of a centromere-specific variants of histone H3, so-called CenH3 [3]. However, within a single organism, centromeric regions often harbor several different types of repetitive sequences, and CenH3 proteins most often preferentially associate with one of them, implying that there may be specific properties inherent to the DNA sequence that contribute to its functional adequacy. The best candidates for such “sequence-encoded” properties could be protein-binding motifs or sequence conformations specific for functional centromeres. However, no known nucleotide motif from satellite repeats, including the best-studied 17-bp CENP-B box from the human alpha satellite [4], passed the test of a ubiquitous, universally functional centromere-specific motif. On the other hand, structural features, although more difficult to recognize, might be more inclusive in centromere specification. The propensity to form secondary structures is highlighted as one of the possible traits that characterize centromere-competent satDNAs [5], arguing that non-B-form DNA structures at centromeres may represent a mechanism for centromere specification [5–8].

Next-generation sequencing boosted the discovery of numerous satellites in genomes, leading to described satellitome collections for individual organisms, some containing over a hundred distinct satellites [9, 10]. The number of publications cataloging newly identified satellites across various species is rapidly increasing, but studies that explore their long-range organization are much rarer and are mainly limited to the most researched model organisms [11–15]. While long-read DNA sequencing by Pacific Biosciences High Fidelity (PacBio HiFi) and Oxford Nanopore Technology (ONT) platforms has launched a new era in satDNA research, the precise assembling of these sequences remains a major challenge and often the weakest point in genome assemblies [16]. High abundance, repetitive nature, and considerable variability of satDNAs, particularly in centromeric regions, often hamper genome assembly efforts especially in satDNA-rich species [17]. Despite these challenges, studying the long-range organization of satDNAs provides valuable insights into their evolutionary dynamics and functional significance, justifying ongoing efforts to include these regions in genome assemblies [18].

The black flour beetle *Tribolium madens* (Charp.) [19] belongs to an insect genus that includes some of the most important pests of stored products, including the well-known Coleoptera model species *Tribolium castaneum* [20, 21]. Beyond their economic importance, *Tribolium* beetles have also become valuable models for studying satDNAs, as satellites prevail in their genomes [22]. In *T. madens*, two satDNAs have been identified so far, the major and minor satellites, which account for 30% and 4% of the genome, respectively [23]. In situ hybridization revealed that the two satellites colocalize within the (peri)centromeric regions [23], and pulsed-field gel electrophoresis (PFGE) corroborated with fiber-FISH experiments suggested that their arrays intermingle [24]. Cloning of minor satDNA repeats also captured a fragment representing the junction of the two satellites, confirming their close association [25]. However, the organization of these satellites has not been analyzed at a fine scale. There is also no information on the presence of other satDNAs in *T. madens*.

In this study, we investigate the satellitome of *T. madens*. To this end, we generated a PacBio HiFi assembly enriched for repetitive sequences and complemented it with Illumina sequencing for assembly-independent detection of tandem repeats. By integrating these two WGS approaches, we characterized the satDNA repertoire of the genome and explored the organization of its most abundant components. Focusing on dominant satDNAs and their genomic arrangement, we examine patterns of large-scale organization and compare them with those observed in closely related *Tribolium* species, thereby placing the satellitome of *T. madens* in a broader evolutionary and potentially functional context.

## Results

### *Tribolium madens* whole-genome sequencing and identification of satDNA candidates

To identify *T. madens* satDNAs by assembly-independent detection, we sequenced the *T. madens* genome using Illumina sequencing to generate short whole-genome reads for TAREAN analysis. TAREAN, a computational tool designed for assembly-free identification of satDNAs from short reads [26], is particularly valuable for detecting satDNAs in organisms with high satDNA content, where accurate genome assembling is challenging or unfeasible. Seven independent TAREAN analyses, each using different randomly subsampled short-reads dataset of a low genome coverage (< 1x), yielded 256 clusters of satDNA candidates (Additional file 1: Table S1). After removing duplicates using an all-to-all self-BLAST, we created a custom database containing consensus sequences of potential satDNAs.

The next step was to verify the repetitiveness of the potential satDNAs by mapping their repeats on the *T. madens* NCBI reference genome Tmad_KSU_1.1, the Illumina-based assembly consisting of 112 scaffolds. We first annotated the two previously described satDNAs, which make up > 30% of the genome [23], but only 0.08% of the Tmad_KSU_1.1 assembly corresponded to these abundant satDNAs. This indicated that tandemly repeated sequences are severely underrepresented in the current reference assembly, which thus proved inadequate for studying the long-range organization of satDNA sequences.

**Fig. 1:**
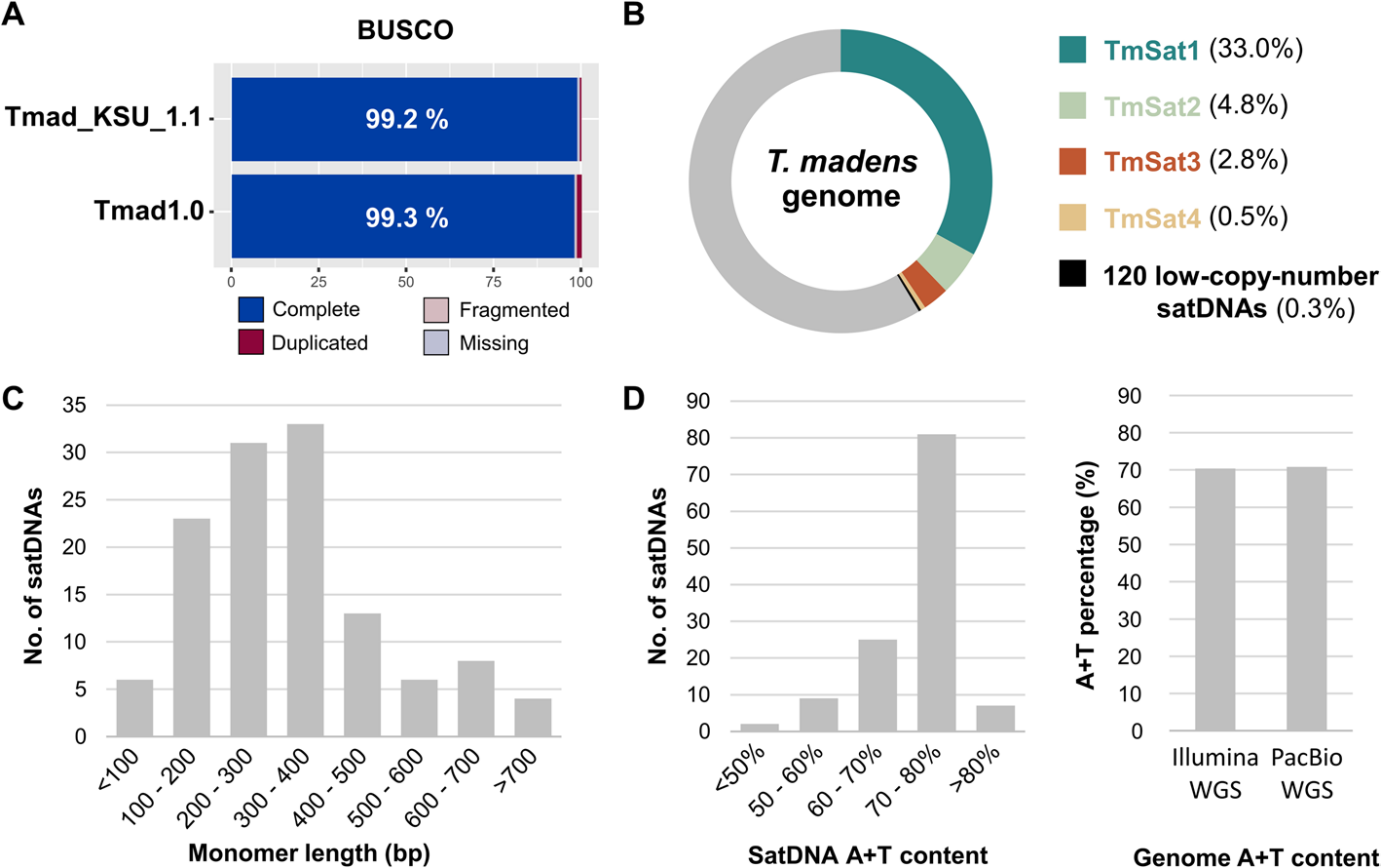
General characteristics of the *T. madens* genome assemblies and the satellitome. **A** Gene completeness assessment according to BUSCO analysis of the *T. madens* genomes assemblies Tmad_KSU_1.1 (NCBI RefSeq assembly GCF_015345945.1) and Tmad1.0 (this work), using the lineage dataset endopterygota_odb10 and gene predictor AUGUSTUS. **B** The proportion of 124 satDNAs in the *T. madens* genome, according to TAREAN estimates. **C** Monomer length distribution of 124 *T. madens* satDNA consensus sequences. **D** A + T content of 124 *T. madens* satDNA consensus sequences, and A + T content of the *T. madens* genome, according to Illumina WGS short reads and PacBio HiFi WGS long reads obtained in this work

To provide an assembly with better representation of repetitive DNAs, we sequenced the *T. madens* genome using highly accurate long-read PacBio HiFi sequencing. We obtained a total of 22.8 Gb of raw sequencing data, corresponding to 76 × genome coverage, that we assembled using the Hifiasm assembler. The resulting assembly, named Tmad1.0, comprises 363 Mb of genomic sequence positioned in 409 contigs (Additional file 1: Table S2). Although contig-level, the Tmad1.0 assembly has a N50 score of 13.8 Mb, which confirms its great contiguity. The quality of the Tmad1.0 assembly was also assessed by BUSCO analysis, which confirmed > 99% gene completeness, comparable to that of the Tmad_KSU_1.1 assembly (Fig. 1A, Additional file 1: Table S3). Importantly, with comparable high quality in the coding regions, Tmad1.0 included far more repeats of the two most represented satellites, 759,584 in Tmad1.0 compared to 505 in Tmad_KSU_1.1, showing markedly improved representation of repetitive sequences.

After generating the repetitive DNA-enhanced Tmad1.0 assembly, we mapped the TAREAN-defined consensus sequences to it by applying a ≥ 70% sequence similarity criterion. We inspected the annotations and classified as satellites those candidates with at least five tandemized copies in the assembly. Consistent with this approach, in this study we operationally define satDNAs as tandemly organized sequences identified by graph-based clustering that contain five or more consecutive repeat units in the assembly. Using this definition, we identified 124 satDNAs, including two previously known [23] and 122 newly discovered (Additional file 1: Table S4).

Annotation of 124 satDNA consensus sequences under the described conditions revealed 775,720 repeats in Tmad1.0 in contrast to 1,269 in Tmad_KSU_1.1, confirming the substantially higher satDNA content in our assembly (Additional file 1: Table S5). Therefore, we used it for all subsequent satDNA analyses.

Given the potential interplay between transposable elements (TEs) and satDNAs in shaping satellitome evolution, we evaluated whether any of the 124 satDNAs might be TE-derived. Using the Tmad1.0 assembly, we generated a TE library with the Earl Grey pipeline [27] (Additional file 2: Fig. S1), annotated all TE copies across the genome, and assessed the overlap with the 124 satDNA loci. Although 79 satDNAs showed partial overlap with at least one Earl Grey annotation, in all cases the predominant number of overlaps corresponded to low-complexity sequences, simple repeats, satellites, or unclassified elements (Additional file 1: Table S6). In addition, we screened the consensus sequences of all 124 satDNAs against the Repbase collection of repetitive elements [28]. Only partial similarities, limited to short or discontinuous segments (Additional file 2: Fig. S2), were detected for 84 satDNAs, including 33 that matched more than one TE, often from different TE classes (Additional file 1: Table S7). Taken together, these analyses provided no evidence that any *T. madens* satDNA, in the entirety of its repeat unit, is unambiguously derived from a TE.

### Satellitome characterization and in situ localization of most prominent satDNAs

The 124 satDNAs that we identified in *T. madens* account for 41.4% of the genome (Fig. 1B, Additional file 1: Table S8). According to TAREAN estimates, the two most abundant satDNAs, here named TmSat1 and TmSat2, represent 33.0% and 4.8% of the genome, respectively. Additionally, we identified two other satDNAs with substantial copy numbers, TmSat3 and TmSat4, comprising 2.8% and 0.5% of the genome, respectively. We classified these as moderately abundant satellites. Of the remaining 120 satDNAs, none individually makes up more than 0.05% of the genome (Additional file 1: Table S8), and together they constitute only 0.3% of the total genome. They are thus categorized as low-copy-number satellites (Fig. 1B).

Regarding repeat unit length, the *T. madens* satDNAs range from 55 to 1275 bp, with 87 of them having monomeric units between 100 and 400 bp (Fig. 1C, Additional file 1: Table S8). Only two satDNAs possess monomers longer than 1 kb, one of which is the moderately abundant satellite TmSat3, with a repeat unit of 1264 bp. Despite the diverse genomic contributions and repeat unit lengths, we found that most satDNAs in *T. madens* exhibit a pronouncedly high A + T content. The majority of these satDNAs have an A + T composition between 70 and 80% (Fig. 1D, Additional file 1: Table S8). Our WGS revealed that *T. madens* has an overall A + T-rich genome, with 70.4% A + T content based on Illumina reads and 70.8% based on PacBio HiFi reads (Fig. 1D). Obviously, the high A + T content of the genome is largely driven by the emphasized A + T composition of the satellitome.

We next performed fluorescence in situ hybridization (FISH) on metaphase chromosomes to determine the chromosomal position of the most prominent satDNAs. The karyotype of *T. madens* (2n = 20) consists of 18 autosomes, a pair of sex chromosomes (XX in females, Xy_p_ in males), and variable number of small supernumerary (B) chromosomes [29]. On the chromosome spreads that we prepared from male gonads, we most often observed 11 large chromosomes (five pairs of autosomes and the chromosome X), along with 10–12 smaller chromosomes (four pairs of autosomes, the male sex chromosome y_p_, and 1–3 supernumeraries).

**Fig. 2:**
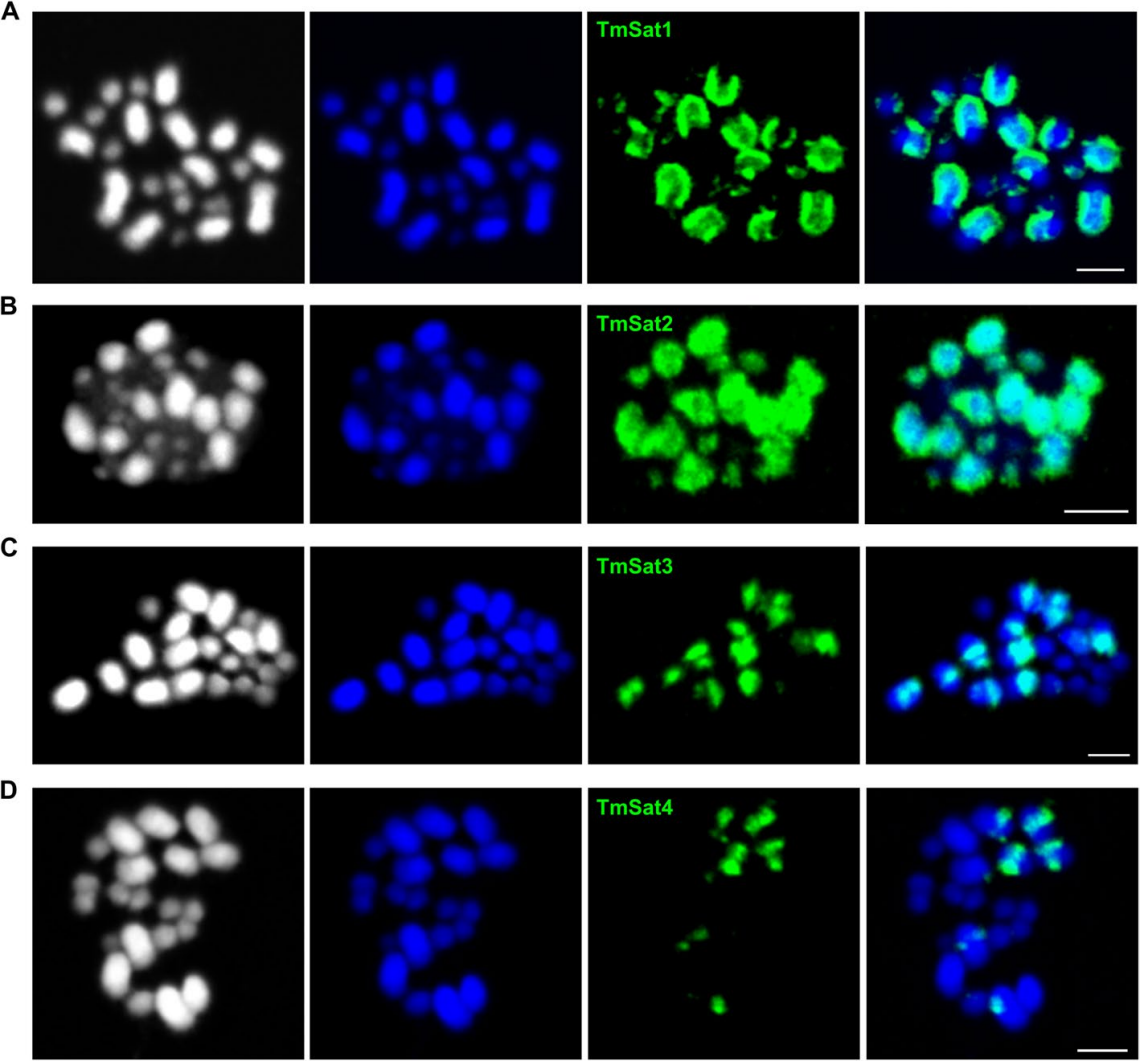
Localization of satDNAs TmSat1 (**A**), TmSat2 (**B**), TmSat3 (**C**), and TmSat4 (**D**) on the *T. madens* metaphase chromosomes determined by fluorescence in situ hybridization. The first panels show the chromosomes (2n = 20 + supernumeraries) in a black and white version to better visualize the contours of the chromosomes. The chromosomes are stained in DAPI (blue fluorescence), while satDNA-specific probes are shown in green. The bar represents 3 μm

The previous study [23] has shown that the two most abundant satellites, the major satDNA TmSat1 and the minor satDNA TmSat2, are present on all chromosomes of the complement, including supernumeraries. This finding was confirmed by our FISH analysis (Fig. 2A, B). The colocalization of these two satellites in the extensive heterochromatic regions, which are brightly stained by DAPI, is particularly noticeable on the large chromosomes (Fig. 2A, B). On the 11 large chromosomes, we also detected strong signals from TmSat3 (Fig. 2C). The less abundant TmSat4 was identified on 8 large chromosomes, showing pronounced signals on two pairs, moderate signals on another pair, and very weak signals on yet another pair (Fig. 2D).

In addition to localization of highly and moderately abundant satDNAs, we also examined the chromosomal position of seven low-copy-number satellites, TmSat5 to TmSat11 (Additional file 2: Fig. S3). Among these, TmSat5 (Additional file 2: Fig. S3A), TmSat8 (Additional file 2: Fig. S3D) and TmSat11 (Additional file 2: Fig. S3G) were detected exclusively on a pair of small chromosomes, while TmSat10 (Additional file 2: Fig. S3F) was localized to a pair of large chromosomes. In contrast, TmSat6 (Additional file 2: Fig. S3B), TmSat7 (Additional file 2: Fig. S3C) and TmSat9 (Additional file 2: Fig. S3E) showed a broader distribution, being present on multiple chromosomes, including both large and small ones. In general, regardless of their presence on one or more chromosomes, the weaker intensity of signals from the low-copy-number satDNAs is in line with their low abundance in the genome, predicted by TAREAN (Additional file 1: Table S8).

### Organization of the highly abundant TmSat1 and TmSat2 satDNAs

The pioneering study on the major satellite TmSat1 and the minor satellite TmSat2, based on 225 bp and 704 bp repeats, respectively, established that both satDNAs harbor related ~ 110 bp subunits, but it also claimed that the complex structure of TmSat2 includes a 408-bp segment of extraneous sequence [23].

Here, we reexamined the minor satellite TmSat2 and corrected its consensus sequence (explained in detail in the Methods section). From the improved consensus we discovered that TmSat2 repeat unit consists entirely of seven related subunits (sub_A – sub_G), ranging from 59 to 113 bp, which share 49.5–92.9% similarity among them (Additional file 2: Fig. S4A, B). The TmSat2 subunits also show 50.5–74.35% similarity to the two TmSat1 subunits mad1_1 and mad1_2 (Additional file 2: Fig. S4B). In terms of intersatellite subunits’ similarities, mad1_1 and mad1_2 are most similar to sub_C and sub_D (Additional file 2: Fig. S4C), which are directly inverted within TmSat2. Regarding inverted sequences, in all nine subunits of TmSat1 and TmSat2 we identified short (< 10 bp) inverted repeats that could potentially adopt simple secondary structures, such as small hairpins (Additional file 2: Fig. S5A). In addition, we discovered that the TmSat2 subunits, which are arranged in opposite orientation within a monomer, form ~ 300 bp long, imperfect inverted repeats, resulting in a huge, ~ 600 bp palindrome-like sequence. Consequently, the TmSat2 repeat may be capable of forming a substantially larger dyad symmetry-based structure (Additional file 2: Fig. S5B) than previously predicted [23]. According to the predicted thermodynamic stability of potential non-B DNA structures, the minimum free-energy (ΔG) values of the seven TmSat2 subunits (sub_A–sub_G) range from −0.59 kcal/mol to −5.21 kcal/mol, whereas the predicted secondary structure of the full TmSat2 monomer shows a markedly lower minimum free energy (ΔG = –133.78 kcal/mol, Additional file 2: Fig. S5). Theoretically, this large difference indicates a much greater thermodynamic potential for the TmSat2 monomer to adopt a dyad-symmetry–based fold.

**Fig. 3:**
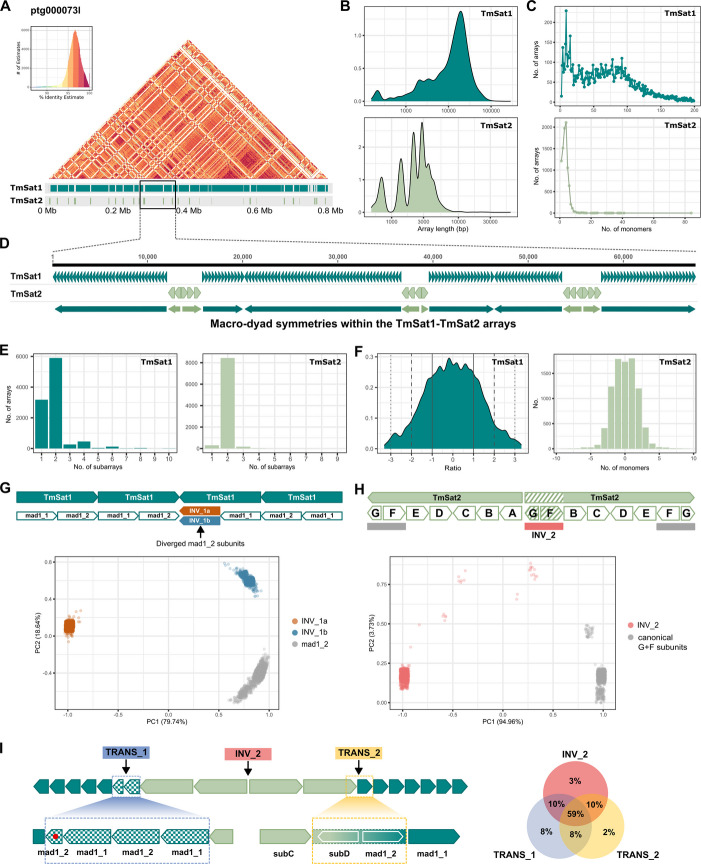
The organization of the major satDNA TmSat1 and the minor satDNA TmSat2 in the *T. madens* genome. **A** ModDotPlot visualization of regions comprising intermingled arrays of TmSat1 and TmSat2. **B** KDE length distribution of the TmSat1 and TmSat2 arrays. **C** Number of monomers in the TmSat1 and TmSat2 arrays. **D** A schematic representation of the TmSat1 and TmSat2 macro-dyad symmetries within multi-megabase sized regions. **E** Number of subarrays in the TmSat1 and TmSat2 arrays. **F** Distributions of length differences between direct and inverted subarrays in the TmSat1 and TmSat2 arrays. **G** A schematic of the inversion sites in TmSat1 arrays with the indicated diverged subunits INV_1a and INV_1b at the inversion point. The PCA clustering of INV-1a, INV_1b and the canonical mad1_2 subunits (2000 randomly selected sequences for each subunit) is shown below the schematic. **H** A schematic of the inversion site in TmSat2 arrays with the indicated INV_2 segment (diverged sub_G and sub_F subunits marked by diagonal stripes) at the inversion point. The PCA clustering of the INV_2 segment and the canonical sub_G + sub_F segments (1000 randomly selected sequences for each category) is shown below the schematic. **I** A schematic illustrating the TRANS_1 and TRANS_2 transition zones between TmSat1 (dark green) and TmSat2 (light green) arrays, with the structure of the transition zones shown enlarged. In the TRANS_1 schematic, the red dot indicates ~ 60 bp deletion within the distal mad1_2 subunit. On the right, a Venn diagram shows the presence of TRANS_1, TRANS_2 and the inversion site INV_2 in the 5764 TmSat2 arrays analyzed

The intermingling of TmSat1 and TmSat2 arrays in the (peri)centromeric regions has been suggested by PFGE and fiber-FISH experiments with limited resolution [24]. Here, benefiting from the high accuracy of PacBio HiFi sequencing, we studied organization of TmSat1/TmSat2 repeats at the ultra-fine, nucleotide-level resolution. To this end, we used SatXplor, a pipeline designed for the comprehensive characterization of satDNAs in complex genome assemblies [30]. First, by annotating TmSat1 and TmSat2 repeats and defining their arrays in the Tmad1.0 assembly, we identified contigs containing multi-megabase sized regions, that are composed of highly intermixed arrays of the two satellites (Fig. 3A). The size of these regions suggests that they correspond to the large chromosomal areas in which the overlap of the TmSat1 and TmSat2 FISH signals was detected (Fig. 2A, B). Additional file 2: Fig. S6 shows ModDotPlot visualization of several representative multi-megabase TmSat1-TmSat2 regions, the largest of which is 9.7 Mb long. The high homogeneity of the ModDotPlot patterns across these huge regions might indicate that TmSat1 and TmSat2 arrays, as the predominant sequences of these putative pericentromeric domains, could also potentially contribute to the composition of the centromeric regions. Within the multi-megabase sized regions, some very long arrays composed of a single satellite were identified, up to 288 kb for TmSat1 and up to 60 kb for TmSat2 (Additional file 1: Table S9). Despite these extreme lengths, continuous arrays consisting of only one satellite are, on average, significantly shorter, with a median length of 14 kb for TmSat1 and a median length of 2.2 kb for TmSat2 (Fig. 3B, Additional file 1: Table S9). Accordingly, TmSat1 arrays comprise many more repeats, with arrays containing up to 100 monomers being prevailing, while the majority of TmSat2 arrays contain up to 8 monomers (Fig. 3C). Our data on array sizes differ to some extent from estimates obtained from measurements of TmSat1/TmSat2 arrays on extended DNA fibers [24]. The discrepancies may be caused by the limited resolution of the experimental stretching and measurement of DNA fibers, coupled by limited number of arrays measured [24], but a partial misassembly in the bioinformatic approach cannot be ruled out either. However, both approaches support a pattern of intense intermixed organization of longer TmSat1 arrays occasionally interrupted by short TmSat2 arrays containing mostly a few repeats.

Regarding the intermingling of *T. madens* major and minor satDNAs, our analyses at the monomer-level resolution revealed a remarkable feature of the TmSat1-TmSat2 organization that was not detected in the previous studies. Namely, we discovered that both satellites, despite the differences in their arrays’ length, form huge palindrome-like structures at the array level. Specifically, both satellites exhibit changes in monomer orientation within their arrays, leading to the formation of several kb long inverted subarrays (Fig. 3D). The number of subarrays may vary, but both satellites predominantly contain two subarrays within their arrays (Fig. 3E), highlighting the formation of macro-dyad symmetries. The length of these macro-dyad symmetries ranges from a few kilobases to several tens of kilobases. The longest macro-dyad symmetries that we annotated in the Tmad1.0 assembly are 75 kb for TmSat1 and 26 kb for TmSat2 (Additional file 1: Table S10). Even though the lengths of the direct and inverted subarrays oscillate, the arrays of both satellites tend towards general symmetry at the genome level (Fig. 3F). Notably, for TmSat1, we found that one-directional arrays (i.e. those that do not contain subarrays) tend to be shorter than those containing one or more subarray inversions (Additional file 2: Fig. S7), suggesting that macro-dyad symmetries might be favored by longer arrays.

Prompted by their frequent occurrence, we investigated how conserved these macro-dyad symmetries are at the sequence level. We first focused on the inversion sites where the subarrays change orientation. For TmSat1, we identified two predominant inversion types, named INV_1a and INV_1b (Fig. 3G). In both cases, one of the opposing mad1_2 subunits is degenerate, showing 23.5% (INV_1a) or 10.7% (INV_1b) divergence from the standard mad1_2 subunit (Additional file 2: Fig. S8A). However, these inversion site subunits are very conserved within their respective groups; 2044 detected INV_1a sequences showed 98.9% similarity, while 3486 INV_1b sequences exhibited 98.2% conservation. The PCA analysis, which included INV_1a and INV_1b sequences as well as randomly selected mad_12 subunits distal to inversion sites, showed a clear separation of sequences into the three clusters (Fig. 3G). The high homogeneity of INV-1a and INV_1b subunits and clear distinction from the standard mad1_2 subunits (Fig. 3G) imply a possible functional significance. Predicted non-B-form conformations, which could potentially arise from inverted subunits at inversion sites (Additional file 2: Fig. S8B), could add to this hypothesis. The predicted hairpins have long stems (58 bp in the INV_1a sites, 98 bp in the INV_1b sites), which could contribute to their stability (with ΔG = –30.64 kcal/mol for INV_1a inversion, and ΔG = –54.98 kcal/mol for INV_1b inversion), but they also end in multiple loops that might be recognized by DNA-binding and chromatin-associated proteins.

In TmSat2 arrays, the inversion sites, where TmSat2 repeats change their orientation, involve the juxtaposed subunits sub_G and sub_F (Fig. 3H) instead of subunit sub_A. This inversion segment, named INV_2, is also highly conserved (98.2%) across 6428 analyzed inversion sites. The INV_2-specific subunits sub_G and sub_F differ from the canonical subunits sub_G and sub_F by 12 nucleotide positions (Additional file 2: Fig. S9A), and the PCA plot illustrates their clustering into two distinct groups (Fig. 3H). It can be assumed that the inversely oriented TmSat2 units emerged from the rearrangement event. We hypothesize that the inversion rearrangement could most easily have occurred by ectopic recombination between the highly similar subunits sub_E and sub_B, which share the highest pairwise similarity (92.9%) among the TmSat2 subunits (Additional file 2: Fig. S9B).

After we established that the inversion sites within TmSat1 and TmSat2 arrays are very conserved, we next focused on the junction regions between TmSat1 and TmSat2 arrays. We identified two prevalent types of transition zones, termed TRANS_1 and TRANS_2 (Fig. 3I). Importantly, both TRANS_1 and TRANS_2, though differing in sequence and being usually located on opposite sides of the TmSat2 arrays, do not contain extrinsic elements. Instead, they represent specific derivatives of the TmSat1 and TmSat2 subunits. TRANS_1, 389 bp long transition toward TmSat1 array, is characterized by the first two TmSat1 monomers of increased variability (82.2% and 68.4%) and a distinctive ~ 60 bp deletion within the distal mad1_2 subunit (Fig. 3I, Additional file 2: Fig. S10A). This transition corresponds to the TMADhinf fragment captured in previous restriction digestion experiments [25]. On the other hand, TRANS_2 is a very specific 124 bp long chimeric element generated from the truncated subunit TmSat2 sub_D and truncated subunit TmSat1 mad1_2 (Fig. 3I). The alignment in Additional file 2: Fig. 10B shows that the truncation of the two subunits occurs sharply, being characterized by duplication of the 6-nt motif TTCAAT.

Given the high degree of conservation of INV_2, TRANS_1 and TRANS_2 segments, we were interested in how often these three elements occur simultaneously in TmSat2 arrays. Analysis of the 5764 TmSat2 arrays showed that 3385 of them (59%) include all three elements (Fig. 3I). Therefore, we conclude that the minor satellite TmSat2 is preferentially embedded in the major satellite TmSat1 in the form of a “cassette”. Based on macro-dyad symmetry, the “cassettes” are defined by a specific inversion point INV_2 and bordering transition zones, TRANS_1 and TRANS_2, within which the number of TmSat2 repeats oscillates. The conservation of the structure frame (dyad symmetry with three defined key points) with some flexibility (varying number of TmSat2 repeats within a “cassette”) suggests that the distribution of the minor satellite TmSat2 within the much more abundant major satellite might have a structural–functional significance.

The assembly of satDNA arrays can lead to a certain degree of misassembling due to their repetitive nature. For that reason, we checked the described organization of the TmSat1-TmSat2 arrays on the raw PacBio HiFi sequencing data. Indeed, we found numerous raw reads demonstrating a mixed arrangement of major and minor satellites based on macro-dyad symmetries. We also confirmed the presence of conserved inversion sites and transition zones between the two satDNAs on the raw HiFi reads. Additional file 2: Fig. S11 shows some of these reads authenticating the TmSat1-TmSat2 organization.

To better understand how TmSat1 and TmSat2 arrays are evolving within the genome, we analyzed the intraspecific variation of their monomer sequences across multiple contigs. We selected the 12 longest Tmad1.0 contigs (> 10 Mb, Additional file 1: Table S11) and randomly extracted annotated monomer copies from TmSat1 and TmSat2 arrays. From these contigs, we also extracted inversion sites (INV_1a, INV_1b, INV_2) and transition zones (TRANS_1, TRANS_2). PCA analyses of these seven datasets showed no pronounced contig-specific clustering; instead, sequences from different contigs largely intermingled (Additional file 2: Fig. S12). To better visualize sequence relationships, we constructed undirected graph networks, which confirmed that although occasional small contig-specific clusters appeared, most clusters contained sequences originating from multiple contigs (Additional file 2: Fig. S13). In addition to supporting conserved structural features across the genome, these results may also indicate ongoing sequence exchange among different arrays, potentially including those located on different chromosomes. Žinić et al. [24] reported associations of non-homologous chromosomes via (peri)centromeric heterochromatin during meiotic prophase I, proposing that such interactions could facilitate satellite sequence exchange. In our cytological experiments, we observed the same bouquet-like formations, and two-color FISH confirmed the overlap of TmSat1 and TmSat2 signals in these three-dimensional associations of non-homologous chromosomes (Additional file 2: Fig. S14).

### Moderately abundant satDNAs TmSat3 and TmSat4

The satDNAs TmSat3 and TmSat4, although not present on all chromosomes (Fig. 2C, D), stand out among the 122 newly discovered satellites due to their genomic share and rather long repeat units. The 1264-bp monomers of TmSat3 occupy 2.8% of the genome, while the 692-bp monomers of TmSat4 account for 0.5%. Analysis of their consensus sequences revealed numerous short inverted repeats, which could potentially lead to the formation of complex secondary structures (Additional file 2: Fig. S15), though with lower predicted stability (ΔG = –56.45 kcal/mol for TmSat3 and –24.34 kcal/mol for TmSat4) compared to TmSat2. Despite the long repeat units, neither TmSat3 nor TmSat4 shows a clearly defined substructure such are those observed in the major and minor satellites.

**Fig. 4:**
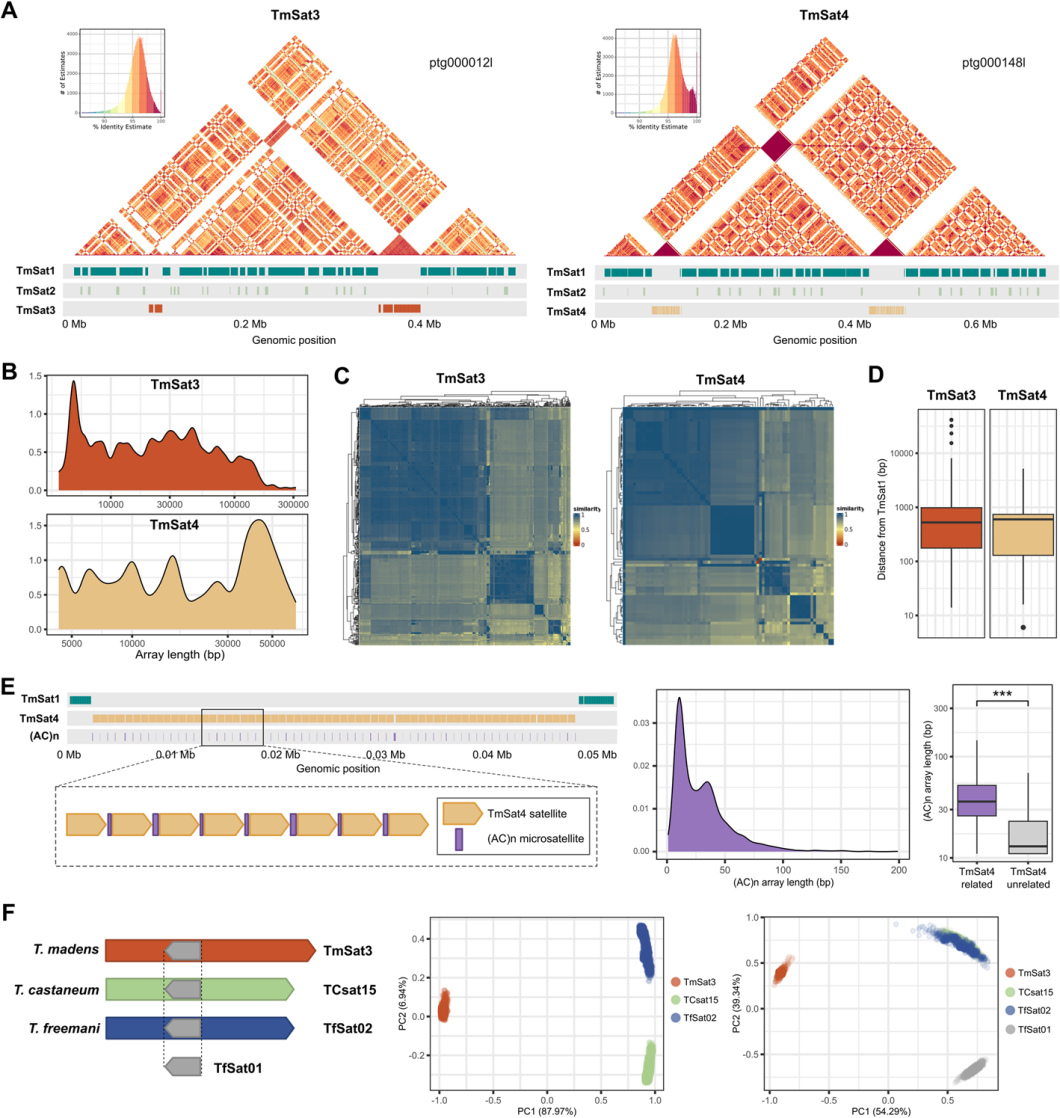
The organization of the satDNAs TmSat3 and TmSat4. **A** ModDotPlot visualization of the regions harboring the TmSat3 and TmSat4 arrays. **B** KDE length distribution of the TmSat3 and TmSat4 arrays. **C** Heatmap visualization of the sequence similarity of the 2 kb flanking regions adjacent to the TmSat3 and TmSat4 arrays. The similarities are indicated by the color-coded legend. The color-coded legend shows the similarities from the lowest (red) to the highest (blue). **D** Box plots showing distances of the TmSat3 and TmSat4 arrays to the nearest TmSat1 arrays. The black line within a box represents the median length distance. **E** The satellite/microsatellite conjunction between TmSat4 satellite repeats and (AC)n microsatellite arrays. The schematic representation of TmSat4 long-range organization (left), the length distribution of (AC)n arrays (center), and the box plot analysis of the length of TmSat4-related and TmSat4-unrelated (AC)n arrays (right). **F** The relationships between the orthologous satDNAs TmSat3 from *T. madens*, TCsat15 from *T. castaneum* and TfSat02 from *T. freemani*. A schematic showing the three orthologous satDNAs and the position of the TfSat01-like segment in them (left). The PCA clustering of TmSat3, TCsat15, and TfSat02 repeats (center). The PCA clustering of TfSat01 and TfSat01-like segments from TmSat3, TCsat15 and TfSat02 repeats (right)

By analyzing their annotations in the Tmad1.0 assembly, we discovered that both satellites are embedded between the blocks of TmSat1-TmSat2 arrays (Fig. 4A). The lengths of TmSat3 and TmSat4 arrays vary widely (Additional file 1: Table S9). They are frequently present as single monomers or short stretches, but can also form extensive arrays (Fig. 4B). The longest annotated TmSat3 array spans 314 kb, while the largest detected TmSat4 array reaches 66 kb. These lengths can explain the strong FISH signals that TmSat3 and TmSat4 show on certain chromosomes (Fig. 2C, D). Regarding the arrays’ inner structure, both satellites, occasionally exhibit changes in repeat orientation within arrays, but such switches occur infrequently compared to the major and minor satellites.

To explore the surrounding context of TmSat3 and TmSat4 arrays, we extracted and analyzed 2 kb of their upstream and downstream flanking regions. As can be seen from the heatmap visualizations of the pairwise similarities between the extracted flanking regions, they are quite homogeneous (Fig. 4C). Closer examination revealed that the flanking regions contain mainly TmSat1 satDNA, which is consistent with the finding that most TmSat3 and TmSat4 arrays are located in the vicinity of the TmSat1 satellite, mostly at distances of less than 1 kb (Fig. 4D). By inspecting the junctions between TmSat1 and TmSat3 or TmSat4 arrays, we discovered that these contacts are abrupt, most often involving the junction of truncated repeats from adjacent satellites (Additional file 2: Fig. S16). Nevertheless, these truncations occur in a non-preferential manner, and no dominant transition sequence was identified between the moderately abundant satellites and the major satDNA TmSat1.

Regarding TmSat4, we report a non-standard feature of satDNA long-range organization that we detected in the Tmad1.0 assembly. Namely, the 692 bp long consensus sequence of TmSat4, as defined by TAREAN, starts and ends with (AC)_5_ and (AC)_3_ tracts, respectively (Additional file 1: Table S4, marked in blue). By examining the consecutive repeats within TmSat4 arrays, we found that, in addition to the monomer termini-assigned AC tracts, the TmSat4 repeats are regularly separated by additional (AC)n stretches (Fig. 4E, left panel). Typically for a microsatellite, the length of these intercalary (AC)n spacers varies (Fig. 4E, middle panel), with the longest detected being 418 bp long. We checked all (AC)n sites in the *T. madens* assembly, and established that 59% of them (2047 out of 3458) were linked to the TmSat4 repeats. The remaining 1411 stretches (TmSat4-unrelated) are randomly scattered across the genome with no significant organizational pattern. We also found that TmSat-unrelated (AC)n sites are overall shorter (with a median of 12 bp) than TmSat4-linked ones (with a median of 42 bp) (Fig. 4E, right panel). Thus, of the total 128 kb that the (AC)n microsatellite makes up in the *T. madens* assembly, 80% (103 kb) is linked to the TmSat4 repeats. We emphasize that in the Tmad1.0 assembly we did not find any TmSat4 arrays without (AC)n spacers. We confirmed the presence of (AC)n spacers between the TmSat4 repeats also in the raw HiFi reads (Additional file 2: Fig. S17), which indicates that this satellite/microsatellite conjunction is an authentic TmSat4 long-range organization, no matter how atypical it is for classical satellites.

### Orthologous *Tribolium* satDNAs

Within the genus *Tribolium*, *T. madens* belongs to the castaneum group, which also includes *T. castaneum* and *Tribolium freemani*. *T. castaneum* and *T. freemani* are not only among the most closely related *Tribolium* species, but also have available high-quality genome assemblies [17, 31] and well-characterized satellitomes [32–34]. We therefore utilized these valuable sources to investigate the evolutionary dynamics of *T. madens* satDNAs. We first focused on determining whether the four most abundant *T. madens* satellites have orthologs in *T. castaneum*/*T. freemani* genomes. For this purpose, we used two approaches: 1) a BLAST search using as queries TmSat1/TmSat2/TmSat3/TmSat4 consensus sequences with relaxed parameters to allow the identification of highly diverged sequences, and 2) an analysis based on TmSat-specific k-mers.

For TmSat1, TmSat2 and TmSat4, we were unable to identify orthologs in *T. castaneum* and *T. freemani* using BLAST, even with very relaxed parameters. Next, we generated k-mers (k = 16) from the satDNA consensus sequences and applied an approximate matching strategy using a Levenshtein edit distance ≤ 3 to search for homologous regions across the two genomes. The k-mer analysis revealed the highest similarity only for short stretches of A + T homopolymers, which are preferentially mapped to the A + T-rich segments of the TmSat1, TmSat2 or TmSat4 consensuses (Additional file 2: Fig. S18). As we could not identify any significant common sequence from these A + T-rich k-mers, we assume that the similarity is most likely due to the high A + T content of all three genomes analyzed. In sum, none of the analyses identified recognizable orthologs for TmSat1, TmSat2 or TmSat4.

In contrast, for TmSat3 we unambiguously identified its satDNA orthologs: TCsat15 in *T. castaneum* and TfSat02 in *T. freemani*. The alignment of consensus sequences revealed that the orthologs from *T. castaneum* and *T. freemani* are more closely related to each other, being 85.7% similar, while TmSat3 shares only 66.5% and 67.5% similarity with them, respectively (Additional file 2: Fig. S19A). To support this conclusion with a larger number of repeats, we extracted all TmSat3-like copies mapped in the *T. castaneum* TcasONT and *T. freemani* Tfree1.0 genome assemblies and compared them with 2500 randomly selected TmSat3 copies from *T. madens*. The PCA plot of 6744 aligned repeats clearly separated the sequences into three distinct clusters based on the species of origin (Fig. 4F, left plot). The first principal component (PC1) of the PCA corroborated the closer relationship between the *T. castaneum* and *T. freemani* orthologs compared to TmSat3 repeats.

In the previous study on the *T. freemani* satellitome, we discovered that the satDNA TfSat02, more specifically its 166 bp segment, served as the source of the current *T. freemani* major satDNA TfSat01 [34]. Since the orthologs TCsat15 and TmSat3 also harbor a TfSat01-like segment (the schematic on Fig. 4F), we investigated the relationships between these segments. We extracted TfSat01-like segments from the TfSat02, TCsat15 and TmSat3, and compared them to 2500 randomly subsampled TfSat01 monomers from the *T. freemani* genome. The PCA clearly segregated the TfSat01 monomers in a distinct cluster, separated from TfSat02/TCsat15/TmSat3 segments (Fig. 4F, right plot). This indicates that TfSat01 has undergone extensive proliferation, which differentiated it from the ancestral source sequence. Notably, in this analysis, the TfSat02 and TCsat15 segments form a common, mostly overlapping cluster with a relatively high average similarity of 92.0% (Fig. 4F, Additional file 2: Fig. S19B). At the same time, the TmSat3-originated segments group separately, confirming that the TmSat3 repeats also in the TfSat01-corresponding segment show a greater evolutionary distance from the *T. castaneum/T. freemani* orthologs.

Considering that low-copy-number satellites may also have orthologs in related species, we examined their relationships. Using consensus sequences from the low-copy-number satellites of *T. madens* as queries, we identified orthologous repeats for 12 *T. madens* low-copy-number satDNAs in *T. castaneum* and *T. freemani*. Six of them have orthologs among the previously identified *T. castaneum*/*T. freemani* low-copy-number satDNAs (Additional file 1: Table S8, Additional file 2: Fig. S20). The remaining six *T. madens* low-copy-satellites have related copies in *T. castaneum* and *T. freemani*, but these orthologous copies were not recognized and classified as satDNAs in the previous analyses of the *T. castaneum* and *T. freemani* satellitomes (Additional file 1: Table S8).

**Fig. 5:**
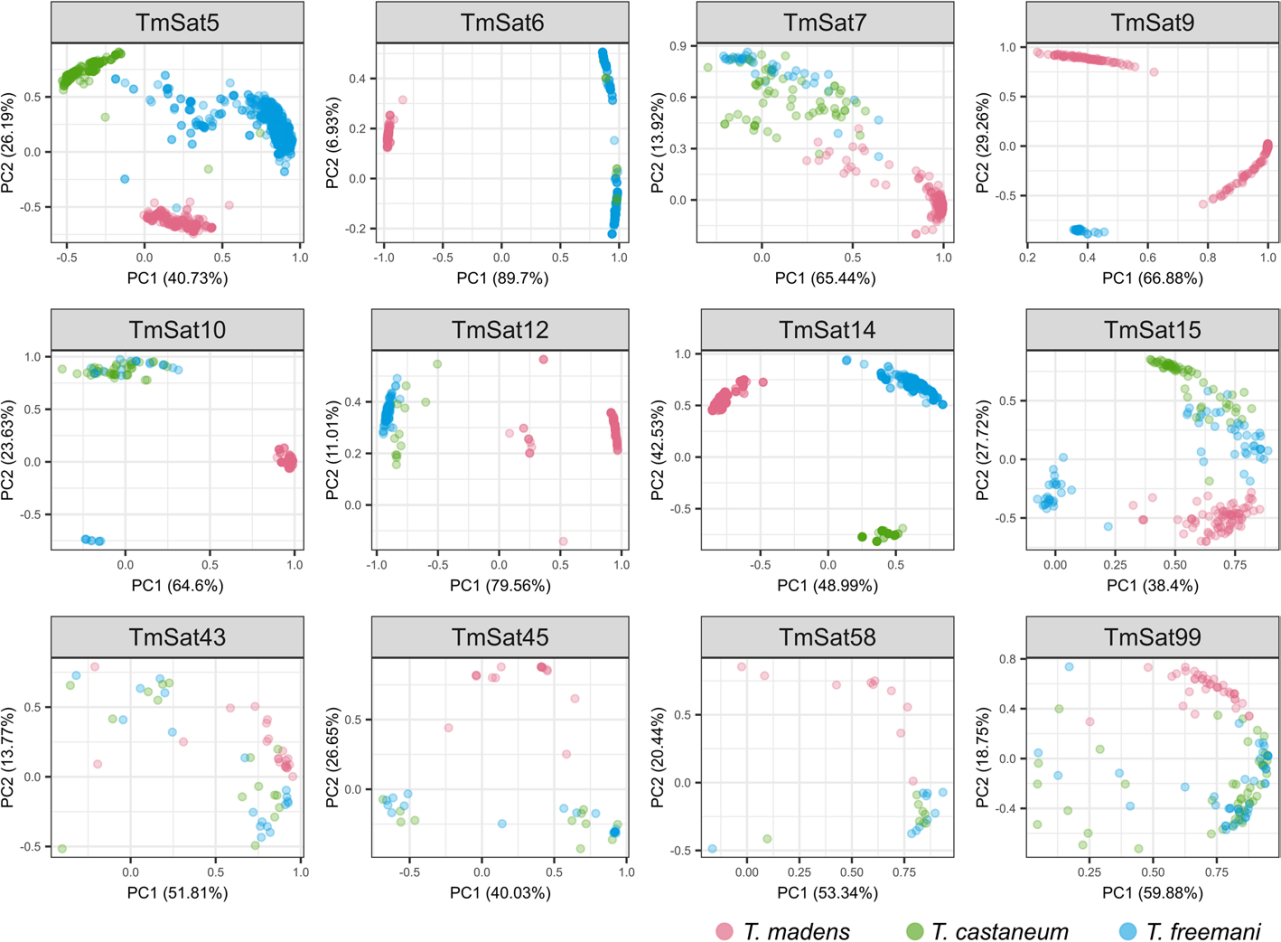
Principle component analysis (PCA) of the *T. madens* low-copy-number satDNAs and their orthologous copies in the congeneric species *T. castaneum* and *T. freemani*. The dots colored according to the species of origin (*T. madens* in red, *T. castaneum* in green, *T. freemani* in blue) represent repeat copies annotated in the *T. madens* Tmad1.0 assembly, the *T. castaneum* TcasONT assembly and the *T. freemani* Tfree1.0 assembly. The proportions of variance for the first two principal components, PC1 and PC2, are shown in brackets on the axes

PCAs of aligned orthologous repeats revealed that the majority of *T. madens* low-copy-number satellites do not mix with orthologous copies from *T. castaneum* and *T. freemani* (Fig. 5). Partial intermingling of *T. madens* repeats was observed only for TmSat7, TmSat43, and TmSat99, but also in these cases the overlap between the *T. castaneum* and *T. freemani* sequences was more pronounced. Overall, the comparison of the low-copy-number orthologs supports the conclusion drawn from the moderately abundant satellite TmSat3, namely that within the castaneum group, *T. madens* is distant to the sibling pair *T. castaneum* and *T. freemani*. Such satDNA correlations among the three congeners are in complete agreement with their phylogenetic relationships inferred from different mitochondrial and nuclear markers [35, 36].

## Discussion

The aim of this work was to explore the satellitome of the flour beetle *T. madens* and to decipher how satellites shape its satDNA-rich genome. A necessary prerequisite for such an analysis was the availability of a high-quality genome assembly with adequate representation of repetitive sequences.

The main obstacle to accurate genome reconstruction is not the genome's size, but the abundance of tandemly repeated sequences [16]. The beetle *T. madens*, with 33% of its genome composed of a single satDNA, is an exemplar of a difficult-to-assemble genome. To the best of our knowledge, none of the currently available T2T assemblies contain any individual satDNA that exceeds this proportion. It is therefore not surprising that the current NCBI reference genome assembly of *T. madens*, Tmad_KSU_1.1 (GCF_015345945.1), is not assembled to the chromosomal level and shows a severe underrepresentation of tandem repeats. We believe that the repetitive regions were excluded from Tmad_KSU_1.1 because they could not be reliably reconstructed from the short Illumina WGS reads used for that assembly. The long-read-based Tmad1.0 assembly, generated in this study, provides far more comprehensive coverage of tandem repeats and, although formally a contig-level assembly, includes several exceptionally long contigs (12–27 Mb) that may correspond to entire chromosomes. It should be noted that the size of the Tmad1.0 assembly (360 Mb) exceeds the experimentally estimated *T. madens* genome size (about 300 Mb) [37]. Several factors may contribute to this discrepancy. One possibility is intraspecific variation in genome size, largely driven by differences in repetitive sequence abundance, which has been documented among individuals and populations in many species [38–40], including *Tribolium* [37]. The variable number of supernumerary chromosomes in *T. madens*, ranging from 0 to 10 [29], may also play a role. In addition, since Tmad1.0 is an unphased assembly, some highly repetitive regions may appear as separate contigs rather than being collapsed. Repeat collapses and expansions are among the most common assembly errors [41, 42] and are practically unavoidable in highly repetitive genomes. Although we cannot exclude the possibility of some repeat-expansion misassemblies in Tmad1.0, all structural findings reported in this study were verified against PacBio HiFi raw reads, confirming the reliability of our conclusions.

In the *T. madens* genome, we identified 124 satDNAs, a number comparable to those reported for other insect genomes [43, 44]. Of note, we defined the *T. madens* satellites based on the presence of ≥ 5 consecutive copies in the assembly, which also captured repeats of very low abundance (< 0.05%). Because such sequences depart from the traditional concept of satellites as highly abundant sequences, we classified them as low-copy-number satellites. Nonetheless, in the propulsive age of satellitome characterizations, it is likely that the field will need to redefine the criteria for satellite sequences or establish a lower threshold for what constitutes a satDNA.

The most consequential finding of our satellitome analysis is the elucidated long-range organization of TmSat1 and TmSat2, the two dominant satellites that together occupy 37.8% of the genome. These satellites were previously described as the major and minor satellites, with a slightly lower estimated genome proportion of 34% [23]. This discrepancy probably results from the use of different methods to estimate satDNA proportions, but it may also reflect individual or population-level variation in the analyzed specimens, including variation in the number of supernumerary chromosomes carrying these satDNAs. Earlier cytogenetic studies showed that these two satDNAs occupy the pericentromeric heterochromatin of all chromosomes [23] and suggested that they may also include regions of the functional centromere [24]. Our high-resolution analyses confirmed that the large TmSat1–TmSat2 domains are highly homogeneous, which could further support this suggestion. However, identifying functional centromeres requires evidence of interactions with centromere-specific proteins such as CenH3. Our work in the related species *T. castaneum* has shown that its unusually elongated centromeres, known as metapolycentromeres, span up to 40% of chromosome length and are built on a repertoire of repetitive sequences dominated by a major satDNA [45]. Based on preliminary tests in which *T. castaneum* CenH3 antibodies failed to recognize *T. madens*, we conclude that *T. madens* encodes a species-specific CenH3. However, the extensive TmSat1/TmSat2 regions lead us to hypothesize that *T. madens* may also possess extended centromeres, with TmSat1/TmSat2 contributing to centromere formation. Yet, without experimental evidence, this remains a speculative hypothesis that requires functional validation. Accordingly, in the following discussion, we refer to TmSat1 and TmSat2 as putative (peri)centromeric satellites to emphasize that their functional status is currently unconfirmed.

**Fig. 6:**
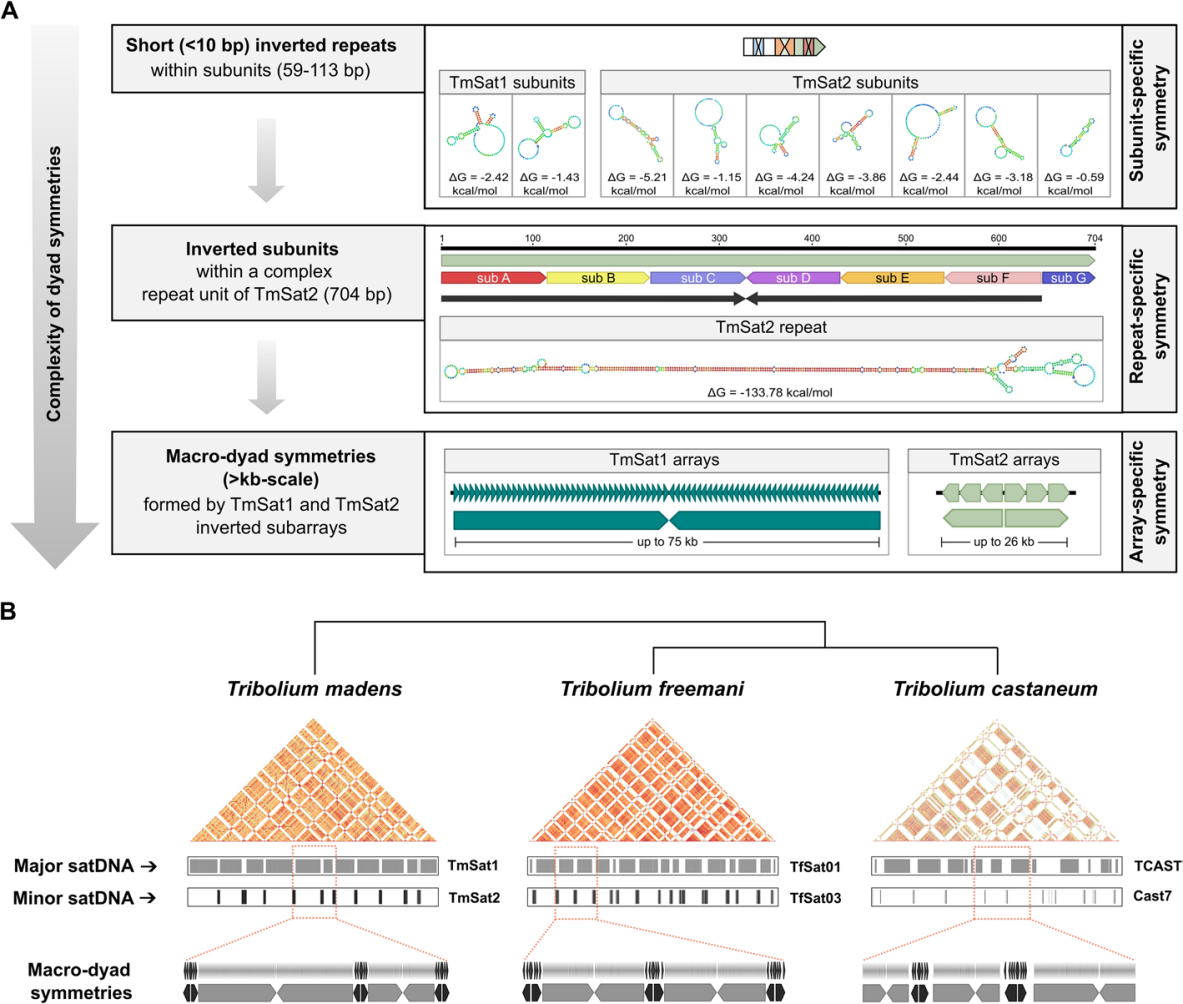
SatDNA-related dyad symmetries in *T. madens*. **A** The three levels of dyad symmetries in the satDNAs TmSat1 and TmSat2: subunit-specific, repeat-specific, and array-specific. Enlarged views of the predicted secondary structures for the TmSat1/TmSat2 subunits and repeats are shown in Additional file 2: Fig. S5. **B** Schematic representation of the long-range organization of major and minor satDNAs within the putative centromeric regions of the three *Tribolium* species: *T. madens*, *T. freemani* and *T. castaneum*. Although the dominant satDNAs differ among these species, their arrays exhibit a common tendency towards macro-dyad symmetry

High-throughput satellitome analysis combined with a high-quality genome assembly enabled us to revise the consensus sequences of the known satellites and to show that, in addition to the major satellite TmSat1, the repeating unit of the minor satellite TmSat2 is entirely derived from an ancestral 110 bp unit. In other words, nearly 38% of the *T. madens* genome originates from a single progenitor sequence. Equally, if not more, remarkable is the discovery that this ~ 110 bp basic sequence, by populating the *T. madens* genome via higher-order repeats, generated three levels of DNA dyad symmetry (Fig. 6A): (1) numerous short (< 10 bp) inverted repeats within TmSat1 and TmSat2 subunits, (2) a large ~ 600 bp dyad symmetry within the 704 bp-long TmSat2 monomers, and (3) extensive macro-dyad symmetries spanning up to several tens of kilobases within both TmSat1 and TmSat2 arrays. The three levels of dyad symmetry, which differ in complexity, strongly suggest biological relevance. Yet, the question remains as to which level is functionally more significant.

Short dyad symmetries generated by < 10 bp inverted repeats, like those observed in TmSat1 and TmSat2 subunits, have been found enriched in centromeric sequences across a wide range of taxa, including Old World monkeys, horse, chicken, fission yeast and several plant species, and have been proposed to play a role in centromere specification [5]. This hypothesis is further supported by data from *Drosophila melanogaster*, whose centromeres are enriched in predicted non-canonical secondary structures, including short dyad symmetries on the Y centromere [7]. In contrast, longer dyad symmetries within satellite repeats, such as that based on ~ 300 bp inverted segments in TmSat2 monomers, are not so common. One of the rare examples involves the 80 bp inverted repeats in the 385 bp satDNA of the parasitoid wasp *Trichogramma brassicae* [46]. Notably, the most prominent intra-monomeric dyad symmetry reported to date occurs in another *Tribolium* beetle, *Tribolium brevicornis*, whose 1061 bp major satellite contains ~ 470 bp long inverted segments [47]. From a theoretical standpoint, for TmSat2, predicted thermodynamic stabilities suggest that formation of a hairpin encompassing the entire monomer is more likely than multiple smaller hairpins within individual subunits. However, these predictions are computational and do not confirm that such structures form in vivo. While it remains unverified whether long inverted segments in *Tribolium* satDNA repeats form intra-monomeric hairpins/cruciforms*,* their presence in putative (peri)centromeric regions is consistent with the model in which such non-B-form DNA structures may facilitate deposition of centromere-specific proteins [5, 48].

Most intriguing is the biological relevance of the macro-dyad symmetries formed by multi-kilobase arrays of *T. madens* major and minor satDNA. Over 50 years ago, it was established by HAP chromatography and electron microscopy that eukaryotic DNA can form hairpin structures up to several kilobases long due to intrastranded inverted repeats [49]. In *D. melanogaster* it was shown that these structures can range from very short to over 15 kb [50]. Given the size of the macro-dyad symmetries in TmSat1 and TmSat2 arrays, we hypothesize that similarly large hairpins could also form in *T. madens*. Functionally, these hairpins might contribute to the organization of the *T. madens* extensive (peri)centromeric chromatin. Variations in subarray lengths suggest flexibility in hairpin stem length, and the highly conserved inversion sites in both TmSat1 and TmSat2 arrays raise the possibility that loops formed at these sites are functionally relevant, potentially serving as protein-binding targets. Conversely, DNA hairpins can compromise genome stability by obstructing replication and causing DNA breakage [8, 51, 52]. Thus, if such hairpins do form, their folding must be flexible, transient, and tightly regulated to balance chromatin stability with dynamic cell-cycle functions.

To date, macro-dyad symmetries formed by satDNA arrays have been documented exclusively within the genus *Tribolium*. Our recent genomic study of the congeneric species *T. freemani* and *T. castaneum* revealed that these species also harbor macro-dyad symmetries composed of multi-kilobase arrays of two intermingled satDNAs, a dominant major and a less abundant minor satellite [34]. Notably, the major satDNAs of *T. freemani*, *T. castaneum* and *T. madens* are entirely unrelated at the sequence level. The minor satDNAs of *T. freemani* and *T. castaneum* show the common origin, but even their repeats have undergone substantial changes and differentiated into utterly species-specific units. It is significant that the three species, while changing the nucleotide composition of the most abundant satellites, have retained a conserved organizational pattern characterized by macro-dyad symmetries in both major and minor satDNAs (Fig. 6B). Moreover, unlike *T. madens*, neither *T. freemani* nor *T. castaneum* shows sequence similarity between their major and minor satDNAs. Taken together, these distinct repertoires of putative (peri)centromeric satellites suggest that the primary sequence of satDNA repeats may be of secondary importance, whereas their organization into dyad symmetries, potentially capable of adopting non-B-DNA conformations, appears to be functionally significant.

We hypothesize that the turnover of the putative (peri)centromeric satDNAs, particularly the major satellites, which are completely divergent among the three *Tribolium* species and comprise 17–33% of their genomes, occurred through amplification bursts potentially associated with speciation. The study of centromere diversity between *Arabidopsis thaliana* and its sister species *Arabidopsis lyrata* detected rapid satDNA diversification occurring through repeated bursts of satellite homogenization in response to centrophilic transposon invasions [53]. The rapid reorganization of centromeric sequences has also been documented in related *Drosophila* species [54]. It is possible that similar mechanisms of high turnover and rapid expansion of different satellite repeats caused interspecific divergence in putative (peri)centromeric regions in *Tribolium* species. This assumption follows the concept of the centromeric paradox, which reconciles conserved centromere function and rapidly evolving centromeric sequences [55]. It could be that, despite extreme nucleotide divergence, *Tribolium* putative centromeric satellites retain similar secondary structures and do not drastically alter the positioning/binding of the centromere-related proteins. Macro-dyad symmetries thus could ensure stable structural conformations keeping favorable environment for kinetochore formation. In this way, even if centromeric DNAs change radically but retain the macro-dyad symmetry, this could provide time for an organism to gradually “refine” the centromeric proteins, which may ultimately lead to reproductive isolation of the emerging species. The closely related siblings *T. castaneum* and *T. freemani*, which can hybridize but produce sterile F1 hybrids [56], offer a suitable system to test this hypothesis.

In *T. madens*, we identified conserved structural features, including preserved inversion sites and transition zones between the frequently intermingling TmSat1 and TmSat2 satDNA arrays. While these segments may have functional significance, the preservation could also reflect mechanisms of their distribution and exchange. In *T. freemani*, which exhibits the same pattern of intense intermingling between the major and minor satDNAs, we detected their arrays in extrachromosomal circular DNAs as well as in bouquet-like 3D formations during meiotic prophase I. We proposed that these mechanisms facilitate the concurrent spread and homogenization of the two satellites through interchromosomal exchange [34]. Given that the *T. madens* major and minor satDNAs were also observed in meiotic bouquet-like associations both previously [24] and in the present study, we believe that the same mechanisms may drive the concomitant spread and homogenization of the TmSat1-TmSat2 arrays.

The satellitome analysis showed that *T. madens* also contains two moderately abundant satDNAs, one of which, TmSat3, is evolutionarily particularly interesting. Orthologs of TmSat3 exist in *T. freemani* and *T. castaneum*, and their sequence divergence mirrors the phylogeny of the three species, confirming that some satDNAs can have a good phylogenetic signal. More importantly, the existence of the three orthologs proves that a segment of their ancestral sequence was propagated into the current major satellite of *T. freemani*, as suggested in our study of the *T. freemani* satellitome [34]. The fact that no copies of the *T. freemani* major satellite were found even in the closest relative *T. castaneum* supports the idea that an amplification burst drastically altered the profile of the dominant satDNAs in these closely related species. This observation aligns with other reports on the rapid exchange of repetitive sequences in centromeric regions, which imply a connection between centromere evolution and speciation [53, 54].

In addition to macro-dyad symmetries, the long-range organization of the *T. madens* satellite regions revealed another structural peculiarity. The repeat units of the moderately abundant satellite TmSat4 alternate with (AC)n microsatellite arrays, forming a composite satellite/microsatellite pattern unusual for “classical” satDNAs. A comparable case was described in the shrimp *Penaeus vannamei*, where ~ 150 bp satellite units were interspersed with 6–15 copies of the CCTAA microsatellite [57]. Given the scarcity of similar records, the significance of such satellite/microsatellite conjunctions remains unclear. However, *D. melanogaster* centromeres show that centromeric satellites can be founded on very short, 5- and 10-bp repeats, and it has been suggested that a recent expansion of short repeats has replaced more complex centromeric repeats [58]. Could satellite/microsatellite conjunctions serve as reservoirs from which short repeats (= microsatellites) can eventually expand into centromeric satellites? We anticipate that as the number of long-read-based genome assemblies increases, additional examples of satellite/microsatellite conjunctions will be discovered across diverse organisms, helping to clarify their possible significance.

## Conclusions

Analysis of the *T. madens* satellitome and the long-range organization of its dominant satDNAs revealed that the multi-megabase regions they occupy are based on macro-dyad symmetries. At present, in the absence of experimental evidence, it remains unclear whether these symmetries have functional relevance or promote the formation of non-B-DNA conformations such as hairpins or cruciforms. However, based on their conservation and localization within large, putative (peri)centromeric regions on all chromosomes, we hypothesize that these sequences may contribute to shaping (peri)centromeric chromatin and possibly to defining centromeres. This idea is further supported by the observation that the (peri)centromeric regions of related species *T. freemani* and *T. castaneum*, although composed of different satDNAs, share the organizational principle of macro-dyad symmetries. The prevalence of macro-dyad symmetries in *Tribolium* points to an intriguing feature of genomic organization, however targeted experimental approaches will be required to elucidate whether this unusual architectural pattern has structural–functional significance.

## Methods

### Animal material

The starter culture of the flour beetle *T. madens* was obtained from the USDA-ARS (Manhattan, Kansas, USA) in 2015 and has been reared as a laboratory culture since then. The beetles were subcultured every four weeks, being propagated in whole wheat flour at 27⁰C and 60% humidity in darkened incubators with internal air circulation.

### PacBio HiFi WGS sequencing

For PacBio HiFi WGS sequencing of *T. madens*, total genomic DNA was extracted from 30 snap-frozen pooled male and female pupae (150 mg) using the Qiagen Genomic tip 100 kit (Qiagen, Germantown, Maryland, USA). DNA isolation and library preparation using SMRTbell Express Template Prep Kit 2.0 (Pacific Biosciences, Menlo Park, California, USA) were performed by the sequencing provider DNA Sequencing Centre (DNASC) at Brigham Young University (Provo, Utah, USA). PacBio HiFi sequencing using the Sequel II System resulted in 1,477,381 HiFi reads with a total length of 22.8 Gb.

### Genome assembly

A total of 22.8 Gb PacBio HiFi reads, with a read N50 of 15,529 bp, were used to generate the *T. madens* genome assembly named Tmad1.0. The high-accuracy of PacBio HiFi reads made them particularly suitable for de novo genome assembly, especially for the resolution of repetitive and complex regions. The genome assembly was performed using Hifiasm v0.16.1 [59], a state-of-the-art assembler optimized for HiFi reads. Hifiasm employs a graph-based approach that is tailored to the high accuracy of HiFi reads and enables haplotype-resolved assembly in diploid genomes. The assembly was conducted without purging haplotypes to retain the full allelic landscape, as our focus was not on generating a collapsed haploid representation but on preserving structural variation and repeat content.

To assess the completeness and quality of the resulting genome assembly Tmad1.0, we used BUSCO v5.8.0 tool [60] with the Endopterygota_odb10 lineage dataset, comprising 2,124 near-universal single-copy orthologs specific to holometabolous insects. BUSCO was run in genome mode using AUGUSTUS as a gene predictor. This provided a robust metric for completeness, fragmentation, and duplication, and offered insights into assembly performance from a functional-genomic perspective.

### Illumina WGS sequencing

For Illumina WGS sequencing, we isolated total genomic DNA from 45 mg of snap-frozen *T. madens* adults (a pooled sample of 12 male and female individuals) using the DNeasy Blood and Tissue Kit (Qiagen, Hilden, Germany). Isolated DNA was quantified using Qubit 2.0 DNA HS Assay (ThermoFisher, Massachusetts, USA), while quality was assessed by Tapestation genomic DNA Assay (Agilent Technologies, California, USA). In the sequencing center Admera Health (South Plainfield, USA), NGS library was constructed using KAPA Hyper Prep Kit (Roche, Switzerland). In order to mitigate index hopping, Illumina® 8-nt unique dual-indices were applied. Equimolar pooling of libraries was performed based on QC values and sequenced on an Illumina NovaSeq X Plus 10B platform (Illumina, California, USA). The Illumina WGS yielded 2 × 17,130,908 paired-end reads (2 × 151 nt), and 5.2 Gb of sequenced data corresponded to approximately 17-fold coverage of the *T. madens* genome.

### SatDNA identification using graph-based clustering

We used the TAREAN pipeline [26] to identify *T. madens* potential satDNAs from the unassembled Illumina reads by a graph-based clustering method. Prior to analysis, the raw Illumina reads were quality-checked by FastQC [61] and preprocessed using the RepeatExplorer2 tools on the Galaxy web server (https://repeatexplorer-elixir.cerit-sc.cz/galaxy/). The interlaced reads were filtered by quality with 95% of bases equal to or above the cut-off value of 10. Qualified reads were subsampled randomly to reduce the size of the input dataset to achieve a low genome coverage skimming datasets. Seven randomly subsampled sets with 168,290 to 1,980,054 input reads, corresponding to genome coverages of ~ 0.1–1x (Additional file 1: Table S1), were used. Through the initial testing of datasets corresponding to different genome coverages, we realized that the highly abundant satDNAs TmSat1 and TmSat2, which comprise more than a third of the genome [23], limit the number of the reads that can be processed to ~ 200,000, even with the option „Perform automatic filtering of abundant satellite repeats “ (Additional file 1: Table S1, analyses T1-T2). Therefore, in the subsequent analyses we filtered reads containing TmSat1 and TmSat2 sequences from the input sets (Additional file 1: Table S1, analyses T3-T7). Consensus sequences of high and low putative satDNAs from seven TAREAN analyses were compiled in a custom database. An all-to-all self-BLAST search was conducted within the custom database to eliminate duplicates. The filtered consensus sequences of the satDNA candidates were then mapped to the *T. madens* genome assembly Tmad1.0. A candidate was classified as satDNA if it was found to be tandemly repeated in an array of at least five tandemized monomers. In that way, 124 satDNAs were identified and cataloged as TmSat1-TmSat124. In order to determine the possible similarity with existing sequences deposited in the NCBI GenBank database [62], satDNA consensus sequences were BLAST-searched against the NCBI GenBank database using the megablast, discontiguous megablast, and blastn algorithms. The TmSat1 satDNA consensus corresponds to the GenBank entry U30598, originally discovered and named as “*Tribolium madens* satellite I” [23]. The consensus sequence TmSat2 shares 90.4% similarity with the GenBank entry U30599 (Additional file 2: Fig. S21A), that was deposited as “*Tribolium madens* satellite II” [23]. Given the almost 10% sequence difference, we evaluated the TmSat2 generated by TAREAN and the sequence U30599. The analysis of the two consensuses annotated in the Tmad1.0 assembly revealed that the TmSat2 sequence is more representative: 1) the mapping using the same criteria resulted in 37% fewer hits for U30599; 2) the distribution of similarities between the detected hits and a query consensus showed a substantial shift to the higher values (> 98%) for TmSat2 (Additional file 2: Fig. S21B), proving that the TmSat1 consensus is more typical. Consequently, we used the TmSat2 consensus in all analyses in this work.

### SatDNA bioinformatics analyses

The basic analyses of satDNA repeats such as monomer length, A + T composition, and mutual pairwise similarities were done using the Geneious Prime 2023.2.1 package (Biomatters Ltd, New Zealand). Secondary structure predictions were done using the RNAfold tool from the ViennaRNA package [63] applying DNA Matthews 2004 energy model. All other analyses were performed as described below, and the scripts used in the analyses have been deposited at Figshare (10.6084/m9.figshare.29134826).

#### Basic and complex annotation workflow

Initial satDNA identifications and simple annotations were conducted using Geneious Prime 2023.2.1 for basic BLAST-based screenings and visual inspections. For more complex analyses, particularly those involving long-range organization and repeat architecture, analyses were performed using a combination of the SatXplor pipeline [30] and custom R scripts.

For satDNA detection and annotation, we used *satannot*, a standalone module of the SatXplor toolkit. As input, we provided a curated set of 124 satDNA consensus sequences generated with TAREAN, which served as the reference query for *satannot*. Parameters used were derived from established best-practice protocols previously described [17, 30], which we refined for our genome:

- satannot annotate sats.fasta genome.fasta --output satannot.gff --threads 24 –perc_id_filter 70 –qcovhsp_filter 70

To prevent redundancy caused by highly overlapping satellite subunits, only the best-scoring hits at a given genomic location were retained using a custom-made script, avoiding nested or tandem duplications in the final annotation set.

#### Array construction and long-range organization

Annotation of satDNA arrays was performed by expanding initial *satannot*-generated monomer annotations by up to one monomer length. Each expanded region was then scanned to detect additional adjacent monomers. If found, the region was further expanded. This iterative extension process allowed us to reconstruct full satDNA arrays, grouping all connected monomers into singular array units, thereby capturing the true span of tandem repeat structures.

To define the inverted repeat structures of TmSat1 and TmSat2 arrays a three-tiered strategy was used:

1. Construct a database of all TmSat1/2 arrays in a “strand-agnostic” manner.
2. Implement the array defining algorithm on specific strands (plus and minus) and overlap them with the database of strand independent arrays.
3. Merge the data to generate a comprehensive map of strand-specific orientation and inverted repeat blocks.

To examine long-range organization, particularly of the TmSat1 and TmSat2 satDNAs, we employed ModDotPlot [64], which enables modular dot-plot analysis sensitive to repeat structure and orientation. ModDotPlot was paired with ggbio [65], an R package for genome visualization, to generate layered visual maps of strand orientation and inversion.

#### Sequence alignments and visualizations

Multiple sequence alignments were performed using MAFFT (v7.505) [66], a fast and accurate alignment tool optimized for large datasets. Most alignments were conducted under the –auto mode, allowing MAFFT to automatically select the optimal strategy based on input complexity and sequence length:

- mafft --auto input_sequences.fasta > aligned_output.fasta

or, for high-accuracy alignments such as those of INV and TRANS sequences:

- mafft --retree 1 --maxiterate 0 input.fasta > output.fasta

For highly divergent sequences or large-scale flanking region comparisons, the L-INS-i algorithm was selected:

- mafft --maxiterate 1000 --localpair input_sequences.fasta > aligned_LINSI.fasta

Genetic distance matrices were calculated in R using the F81 evolutionary model implemented in the *ape* package [67] and used for both principal component analysis (PCA) and graph-based network visualizations. PCA analysis was performed with the FactoMinerR package [68], and the first two dimensions were visualized using ggplot2 [69]. Graph-based networks were constructed by identifying, for each sequence, five closest sequences using the dist.dna function in *ape* under the F81 model, while these relationships were visualized with *igraph* package in R.

#### Flanking region and intersatellite proximity analyses

We analyzed 2 kb flanking regions around TmSat3 and TmSat4 arrays using MAFFT for multiple sequence alignments, followed with clustering and visualization using pheatmap (v1.0.12) in R to explore sequence similarity and transition patterns. Additionally, for each TmSat3/TmSat4 array, we manually identified the closest TmSat1 or TmSat2 arrays and measured the linear distance to estimate proximity to TmSat1/TmSat2 arrays.

#### Junction/transition region analysis

To pinpoint boundaries between TmSat3/TmSat4 satellite arrays and adjacent TmSat1 arrays we used SatXplor’s k-mer edge defining algorithm, a high-resolution method leveraging exact k-mer pattern matching. This approach, which leverages k-mer entropy and positional variance, significantly outperforms traditional BLAST-based junction detection in terms of resolution and false-positive rate.

#### Orthologous satDNA search via k-mer matching

To trace evolutionary origins of TmSat1, TmSat2, and TmSat4 in *T. castaneum* and *T. freemani* we first used a simple BLAST search with permissive parameters, which yielded no pronounced hits. Thus, we decided to employ a dual-pronged k-mer based approach outlined in the following steps:

1. Generate k = 16-mers from consensus satDNA sequences.
2. Run BLASTn (–task short) for these kmers. BLAST failed to yield significant matches thus necessitating the usage of approximate k-mer hits strategy.
3. Perform approximate matching using Levenshtein distance ≤ 3 against the two reference genomes, the *T. castaneum* TcasONT (ENA accession number GCA_950066185.1) and the *T. freemani* Tfree1.0 (NCBI accession number GCA_939628115.1).
4. Pool results from both analyses into a single result table for each genome and create reduced sets of annotations (where multiple consecutive regions have high similarity merge them together regardless of origin) and extract them from their respective genomes.
5. Map the sequences onto TmSatX (where X is 1,2,4) monomers using Bowtie which is best suited for such short sequences.

#### (AC)n microsatellite analysis

To investigate the distribution and relatedness of (AC)n microsatellite arrays with satDNAs, TmSat4 in particular, we conducted a targeted analysis focused on (AC)n repeat motifs, specifically examining stretches of five or more uninterrupted AC dinucleotide repeats (i.e., n ≥ 5). The analysis was performed using the vmatchPattern function from the Biostrings R package (v2.68.1), which enables efficient exact string matching across large genomic sequences. The search was configured to allow zero mismatches, ensuring the identified motifs were precise AC tandem repeats without any nucleotide substitutions:

vmatchPattern("ACACACACAC", genome, max.mismatch=0)

To avoid overlap with TmSat4 annotations, which inherently contain eight AC motifs per monomer as defined by TAREAN, we implemented a subtraction filter from GenomicRanges R package which shortens AC motif ranges within TmSat4 annotation boundaries to isolate microsatellite instances independent of TmSat4 monomers.

#### Evaluation of satDNA-transposon relationships

The potential transposon derivation of the *T. madens* satDNAs was assessed using two approaches. First, TEs in the Tmad1.0 assembly were annotated with the Earl Grey pipeline [27] using default parameters (https://github.com/TobyBaril/EarlGrey). The analysis incorporated an extra Coleoptera-specific partition from the Dfam 3.9 database to improve detection and classification of lineage-specific repetitive elements. We integrated TE and repeat annotations generated by Earl Grey with the satDNA annotations obtained using *satannot*. Only the filtered annotation set provided by Earl Grey was used in downstream analyses to ensure high-confidence repeat calls. Genomic interval overlaps between Earl Grey-derived repeat annotations and satDNA loci were identified using the *foverlaps* function from the *data.table* package in R. The resulting overlaps were then quantified by satDNA family and by the corresponding repeat class assigned by EarlGrey, and the summarized counts are reported. As a second approach, the 124 satDNA consensus sequences were screened with the CENSOR tool [70] against GIRI Repbase (http://www.girinst.org/, accessed on 17 October 2025), a comprehensive database of eukaryotic repetitive DNA elements [28].

### SatDNA probes

We determined the chromosomal localization for eleven *T. madens* satDNAs (TmSat1-TmSat11) using fluorescence in situ hybridization (FISH), and specific DNA probes were used for this purpose. Specific probes for all satDNA were prepared by amplifying fragments of the target satDNA from genomic DNA using PCR, then cloning the fragments into a plasmid vector. Specific PCR primers were designed using Primer3 (v2.3.7) within the Geneious Prime 2023.2.1 software (Biomatters Ltd, New Zealand). Details of the primer sequences and their optimal annealing temperatures are listed in Additional file 1: Table S12. Each PCR reaction contained: 10 ng of genomic DNA, 0.2 μM of each specific primer, 0.2 mM dNTP mix, 2.5 mM MgCl₂, 0.25 U GoTaq G2 Flexi DNA polymerase, 1 × Colorless GoTaq Flexi Buffer (Promega, USA). The PCR program included: (1) initial denaturation at 94 °C for 3 min, (2) 35 cycles of denaturation at 94 °C for 10 s, annealing at the primer-specific temperature for 10 s, extension at 72 °C for 10 s, (3) final extension at 72 °C for 5 min. The amplified satDNA fragments were cloned into the pGEM-T Easy vector (Promega, USA), and transformed into *Escherichia coli* XL10-Gold Ultracompetent Cells (Agilent Technologies, USA), following the manufacturer’s instructions. Positive clones were identified by blue-white screening, and the insert sizes were confirmed by colony PCR using M13F and M13R-40 vector primers. The insert sequences were validated through Sanger sequencing (Macrogen Europe BV, the Netherlands). To represent the sequence variability of satDNA monomers, several clones were usually combined for probe labeling. SatDNA probes were labeled by PCR using cloned inserts as templates and specific primers, incorporating biotin-16-(5-aminoallyl)-dUTP (Jena Bioscience, Germany) or aminoallyl-dUTP-Cy3 (Jena Bioscience). The labeled dUTP and regular dTTP were mixed in a 1:2 ratio in the labeling reactions. For the TmSat3 probe, considering TmSat3 monomer length of 1264 bp, we used four different primer pairs to bridge the entire monomer sequence, using the TmSat3 monomer fragment cloned into the plasmid vector pUC18 as a template. For TmSat2, given the partial sequence similarity between the TmSat1 and TmSat2 satellites, we constructed the TmSat2-specific probe from a 500 bp TmSat2-specific fragment that does not contain subunits sub_C and sub_D, which share the highest similarity to the TmSat1 satellite. The 500 bp TmSat2-specific fragment, cloned into the plasmid vector pUC18, was biotin-labeled by nick translation using the Nick Translation Kit (Roche, Switzerland).

### Chromosome preparations and fluorescence in situ hybridization (FISH)

Chromosome spreads were prepared from gonads dissected from *T. madens* male pupae using the squash technique as described previously [45]. Hybridization using biotin-labeled or Cy3-labeled satDNA probes was conducted overnight (~ 18 h) at 37 °C in a hybridization buffer containing 60% deionized formamide, 8% dextran sulfate, 1.6 × SSC, 20 mM sodium phosphate pH 7.0, and 50–100 ng/μL of the biotin-labeled DNA probe. Following hybridization, slides were subjected to post-hybridization washes in 50% formamide/2 × SSC at 37 °C. Cy3-labeled probes were detected directly after posthybridization washes, while detection of biotin-labeled probes was achieved using a fluorescein-conjugated avidin D (FITC-avidin D) and biotinylated anti-avidin D amplification system (Vector Laboratories, USA). Signal enhancement was performed through a three-step sequential incubation using the following reagent dilutions: 1:500 FITC-avidin D, 1:100 biotinylated anti-avidin D, and 1:2000 FITC-avidin D. Slides were subsequently counterstained with 4′,6-diamidino-2-phenylindole (DAPI) for 15 min, air-dried, and mounted in Mowiol 4–88 mounting medium (Sigma-Aldrich, USA).

### Confocal microscopy and image analyses

Following FISH, the slides were scanned using a Leica TCS SP8 X confocal laser scanning microscope (Leica Microsystems, Germany), equipped with an HC PL APO CS2 63 ×/1.40 oil immersion objective, a 405 nm diode laser, and a supercontinuum excitation laser. FITC and DAPI fluorescence signals were captured separately, and the images were processed using LAS X Office software version 1.4.7 (Leica Microsystems), ImageJ [71] and Adobe Photoshop CS5 (Adobe Systems, USA), applying only global adjustments that uniformly affected the entire image. A minimum of 10 metaphase spreads derived from 3 to 5 independent FISH experiments were analyzed for each satDNA.

## Supplementary Information

Additional file 1: Supplementary Tables S1-S12.

Additional file 2: Supplementary Figures S1-S21.

## Data Availability

The *T. madens* genome assembly Tmad1.0 generated in this study has been deposited in the European Nucleotide Archive (ENA) under the BioProject accession PRJEB89229 [72], with assembly accession GCA_965279765. Raw Illumina whole-genome sequencing data generated in this study have been deposited in the Sequence Read Archive (SRA) at the National Center for Biotechnology Information (NCBI) under BioProject accession PRJNA1255600 [73]. Consensus sequences of 124 *T. madens* satDNAs have been deposited in the NCBI GenBank under accession numbers PV575036-PV575159 [74]. The satDNA collection is cited as one reference in the reference list, and the accession numbers related to individual satDNAs are listed in Additional file 1: Table S4. Scripts used for the analyses in this study are available from Figshare [75]. Publicly available genome assemblies used in this study include *T. madens* Tmad_KSU_1.1 [76], *T. freemani* Tfree1.0 [31, 77] and *T. castaneum* TcasONT [17, 78].
