# Supplementary material for: Tribolium madens satellitome reveals a network of highly abundant satellite DNAs in megabase-sized regions hallmarked by macro-dyad symmetries
Source: Genome Biol. 2026 Mar 7;27:131. doi: 10.1186/s13059-026-04022-0 (PMC13081366; doi:10.1186/s13059-026-04022-0)
Supplement: Supplementary file 2 — Additional file 2: Supplementary Figures S1-S21. [file 13059_2026_4022_MOESM2_ESM.pdf]

Additional file 2: Supplementary figures S1-S21

for the manuscript

Tribolium madens satellitome reveals a network of highly abundant satellite DNAs in megabase-sized regions hallmarked by macro-dyad symmetries

by Veseljak D, Despot-Slade E, Volarić M, Horvat L, Meštrović N, Mravinac B

Fig. S1. Overview of transposable elements (TEs) identified in the Tmad1.0 assembly using the Earl Grey pipeline.

Fig. S2. Alignments of partial sequence segments between *T. madens* satDNAs and Repbase-deposited repetitive elements.

Fig. S3. Localization of the low-copy-number satDNAs TmSat5 **(A)**, TmSat6 **(B)**, TmSat7 **(C)**, TmSat8 **(D)**, TmSat9 **(E)**, TmSat10 **(F)**, and TmSat11 **(G)** on the *T. madens* metaphase chromosomes (2n=20+supernumeraries) determined by fluorescence *in situ* hybridization.

Fig. S4. Structural organization of TmSat1 and TmSat2 repeat units.

Fig. S5. Short (<10 bp) inverted repeats in the subunits of TmSat1 and TmSat2 **(A)**, and predicted secondary structures of TmSat1 and TmSat2 repeat units **(B)**.

Fig. S6. The ModDotPlot visualization of the multi-megabase sized regions containing inter-mingled arrays of TmSat1 and TmSat2 satDNAs in the four contigs (ptg000032l, ptg000011l, ptg000031l, and ptg000014l) from the Tmad1.0 assembly.

Fig. S7. The length of the TmSat1 arrays related to the number of subarrays they contain.

Fig. S8. Diverged subunits INV_1a and INV_1b from the inversion sites in the TmSat1 arrays.

Fig. S9. Structure of the inversion site INV_2 in the TmSat2 arrays and a possible explanation for its formation.

Fig. S10. Structure of the transition zones TRANS_1 and TRANS_2 between the TmSat1 and TmSat2 arrays.

Fig. S11. Visualization of the raw PacBio HiFi sequencing reads (>30 kb) demonstrating the authenticity of macro-dyad symmetries and conserved segments in TmSat1 and TmSat2 arrays.

Fig. S12. PCA clustering of TmSat1 and TmSat2 monomers, as well as INV_1a, INV_1b, INV_2, TRANS1, and TRANS2 segments derived from the 12 longest contigs of the Tmad1.0 assembly.

Fig. S13. Graph networks illustrating sequence-similarity relationships among TmSat1, TmSat2, INV_1a, INV_1b, INV_2, TRANS_1, and TRANS_2 sequences extracted from the 12 longest contigs of the Tmad1.0 assembly.

Fig. S14. Meiotic bouquet-like configurations showing associations among non-homologous chromosomes of *T. madens*.

Fig. S15. Predicted secondary structures of TmSat3 **(A)** and TmSat4 **(B)** repeat units.

Fig. S16. The abrupt transitions between **(A)** TmSat1 and TmSat3 arrays, and **(B)** TmSat1 and TmSat4 arrays.

Fig. S17. A raw PacBio HiFi read consisting entirely of TmSat4 repeats separated by microsatellite (AC)n arrays of different lengths.

Fig. S18. K-mer analysis of the satDNAs TmSat1, TmSat2 and TmSat4, performed to investigate the possible presence of the orthologous satDNAs in the congeneric species *T. castaneum* and *T. freemani*.

Fig. S19. The relationships between the orthologous satDNAs TmSat3 from *T. madens*, TfSat02 from *T. freemani*, and TCsat15 from *T. castaneum*.

Fig. S20. Alignments of the *T. madens* low-copy-number satDNAs and their orthologs among the known *T. castaneum* and *T. freemani* satDNAs.

Fig. S21. Comparison of the TmSat2 consensus sequence defined in this work and the GenBank entry U30599.

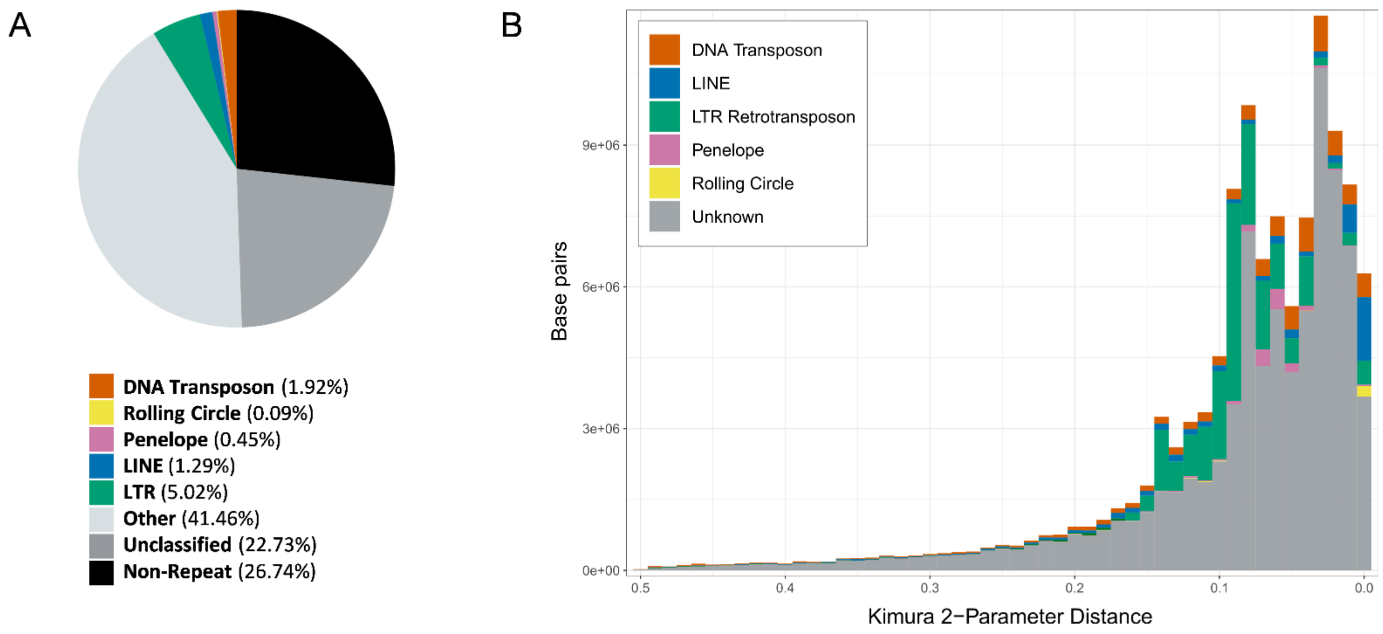

Fig. S1. Overview of transposable elements (TEs) identified in the Tmad1.0 assembly using the Earl Grey pipeline. **(A)** Summary chart showing the proportional representation of major TE classes in the assembly. **(B)** Repeat landscape plot summarizing TE activity using Kimura 2-Parameter Divergence.

TmSat1

Name	From	To	Name	From	To	Dir	Sim	Pos/Mm:Ts	Score
TmSat1	38	191	Harbinger-N3_AT	246	408	d	0.7125	3.7500	84

```
 38 tcaaagtatttcaaccaaatactggctc-taat-aagagttttcttctaaaataaagcaatttgagctat 105
    ||||||||||||||-||| | :||||||-||||- | || ||----- |||||||||-|: :| | ||||
246 tcaaagtatttcaa-caatttttggctcgtaatctacagatt-----aaaaataaag-agactcatctat 308

106 -t-ttc--aatccaatt--ttgctcgat-ttt------aatacagaattaattcaa--aactaa--tgta 158
    -|-|||--||| |-|||--||| ||-||-| |------| |||:||| |||||: |--:| |||--||||
309 ctcttcgtaatgc-attgattgatc-atatatgggagcattacggaaataattttattgagtaatttgta 376

159 ttttattagtataattgcttcaatcttaatttt 191
     ||||| |||-|||||  | :| |:||||||||
377 atttataagt-taatttatgtatttttaatttt 408
```

TmSat2

Name	From	To	Name	From	To	Dir	Sim	Pos/Mm:Ts	Score
TmSat2	85	143	DNA9-3_STu	2301	2353	d	0.8519	4.0000	78

```
  85 aaaataagacgatttgattaattttcaacagattttcttagatt---ttaagccagaaaaca 143
     ||||||||||  |||:|||||||||-||||||-----||||| |---|||||---|||||||
2301 aaaataagactttttaattaatttt-aacaga-----ttagagtgaattaag---gaaaaca 2353
```

Name	From	To	Name	From	To	Dir	Sim	Pos/Mm:Ts	Score
TmSat2	154	356	Gypsy21-PTR_I	12905	13118	d	0.7449	9.0000	72

```
  154 atgtatttcaacacaatacgtgtttaaatagccgttttcagccactataatagaatttacgcaacttta 223
      ||||||||||| | ||| |:|||| ||||-------------|||| | ||-| ||||-|---||----|
12905 atgtatttcaaaaaaatccatgttgaaat-------------cactttcat-gtattt-c---ac----a 12952

  224 atcgatttgg----ctcgattttaaatcaga--aatcgctctaaatca-----atgtatttc----aacg 278
      | |:: ||||----|| ||----|||| |||--|||-|||- ||||||-----|||||||||----|||
12953 aacaggttggtcacctgga----aaataagaccaat-gct-gaaatcactttcatgtatttcacaaaact 13016

  279 a---tgaaataac-----tgt-tttcagtcaaaa-t------aaagcaatttgagctatttc-c-aattc 330
      |---|||||| ||-----|||-|||||--|||||-|------||| || ||| |  ||||||-|-|| ||
13017 aatctgaaatcactttcatgtatttca--caaaactaatctgaaatcactttcatgtatttcacaaaatc 13084

  331 cattttg------cttt-at-ttttgcagaaaac 356
       ||||||------||||-||-| || || |||||
13085 aattttgaaatcactttcatatatttcacaaaac 13118
```

Name	From	To	Name	From	To	Dir	Sim	Pos/Mm:Ts	Score
TmSat2	394	427	RTEX-3_CaAu	4730	4766	c	0.8889	1.0000	72

```
 427 atttggctcaa--tttt-aaaccagtaattgaaccaa 394
     |||||||||||--||||-|||:||:||||||||||||
4730 atttggctcaaacttttcaaatcaataattgaaccaa 4766
```

TmSat3

Name	From	To	Name	From	To	Dir	Sim	Pos/Mm:Ts	Score
TmSat3	212	264	Kolobok-1_SIn	2179	2248	c	0.8929	2.0000	84

```
 264 aaaaagtgaaaa---------tt------gtaaaaat---tgtgaaactttaaacgcgtttttctacgaa 213
     ||||||||||||---------||------||| ||||---|||:|||||||||||||||||||||-||||
2179 aaaaagtgaaaaattctcatcttatacacgtataaatgtatgtaaaactttaaacgcgtttttct-cgaa 2247

 212 a 212
     |
2248 a 2248
```

Name	From	To	Name	From	To	Dir	Sim	Pos/Mm:Ts	Score
TmSat3	409	436	GYPSOR1_I	4719	4748	d	0.9310	99.0000	68

```
 409 gaaactaaaggagatagccc--aatgtggt 436
     |||||||||||||||||| |--||||||||
4719 gaaactaaaggagatagcacagaatgtggt 4748
```

Name	From	To	Name	From	To	Dir	Sim	Pos/Mm:Ts	Score
TmSat3	473	520	EnSpm-14N1_DR	1527	1576	c	0.7959	1.8000	72

```
 520 atctagtgtttttgaacatgtctcagat--tcgatttaaacctatcttat 473
     |||||:|||||||||| ||||||:||||--:| | ||||||:| |:||||
1527 atctaatgtttttgaaaatgtcttagatgacctaattaaactttttttat 1576
```

Name	From	To	Name	From	To	Dir	Sim	Pos/Mm:Ts	Score
TmSat3	590	643	Transib-N1_CQ	908	954	c	0.8163	3.5000	68

```
643 ccaatcgattccccggaacattttacataggtataggtcaaaatattatgtcat 590
    ||||||||||||||||| : |||||||||:| |------| | ||||||-||||
908 ccaatcgattccccggatttttttacataagaa------acactattat-tcat 954
```

Name	From	To	Name	From	To	Dir	Sim	Pos/Mm:Ts	Score
TmSat3	715	790	Academ-1_AP	6553	6629	c	0.7692	4.0000	84

```
 790 aaaattaaattttctgacatat--attaaaa--atttcagtgctc-tacgatgaatagttatcgagaaaa 726
     |||||||  ||||||  ||| |--|||| ||--||||:--||-||-|| :|| |||||||||||||||||
6553 aaaattattttttctttcatttttattataagtatttt--tg-tcgtaaaatcaatagttatcgagaaaa 6619

 725 tttctgataaa 715
     |:||-|| |||
6620 tctc-gaaaaa 6629
```

TmSat4

Name	From	To	Name	From	To	Dir	Sim	Pos/Mm:Ts	Score
TmSat4	202	271	piggyBac-5_XL	6057	6124	c	0.7857	4.0000	74

```
 271 cttcattttaaa-atgt-ggcatagtatttcgaaataagatcgaacaacgcatgcatctcgcca-a--ca 207
     || |||||:|||-|| |-|||-||| |||-----||| || |-||||||||||||| ||||:||-|--||
6057 ctgcatttcaaacatttaggc-taggatt-----atacgaac-aacaacgcatgcaactcgtcacatgca 6119

 206 acaaa 202
     |||||
6120 acaaa 6124
```

Name	From	To	Name	From	To	Dir	Sim	Pos/Mm:Ts	Score
TmSat4	274	320	Transib-2_CoFl	1885	1931	d	0.7917	2.6667	68

```
 274 cacttaaaaagaaaacgcaacc-tatattacagtaaaggtgtatgtat 320
     ||||||||||||:|||| :||||-||| |||- |||| | |||:|||||
1885 cacttaaaaaggaaacttaaccttatttta-tgtaatgttgtgtgtat 1931
```

Name	From	To	Name	From	To	Dir	Sim	Pos/Mm:Ts	Score
TmSat4	418	457	Gypsy-3_BlaAdu-I	611	651	c	0.8293	6.0000	70

```
457 attaaat-ccaaacgaaatactataccatttttattatttt 418
    |||||||-|| |||:| ||||||| || | |||||||||||
611 attaaatacctaacaacatactattccctatttattatttt 651
```

Name	From	To	Name	From	To	Dir	Sim	Pos/Mm:Ts	Score
TmSat4	606	666	piggyBac-3_DAn	33	91	d	0.7742	3.0000	70

```
606 tgatagttagtaaaaaactc-ttatcttattcttttcatgcttgttgact-atcaattgtgtt 666
    ||||-||||--|||| |-||-||||:||||||||||||| |:| ||||||-|  ||||: |||
 33 tgat-gtta--aaaata-tcgttattttattcttttcattcctttgactcaaaaattaagtt 91
```

TmSat7

Name	From	To	Name	From	To	Dir	Sim	Pos/Mm:Ts	Score
TmSat7	115	165	Academ-8_MyCo	3441	3485	d	0.8298	2.0000	70

```
 115 atttttgcagaaaatgaagcttattataagggcatttgactgtaaaaaaat 165
     || |||:||||:|||-----|||| |-|||||||||| |||||:|||||||
3441 atatttacagagaat-----ttataa-aagggcatttcactgtgaaaaaat 3485
```

TmSat9

Name	From	To	Name	From	To	Dir	Sim	Pos/Mm:Ts	Score
TmSat9	162	208	REP4_XT	180	227	d	0.9167	1.0000	112

```
162 aactgcagaccggtttcgccctt-ttggggctcatcagtgcagtgtag 208
    ||||:||||||||||||||||||-||||||||||||||||:||||:||
180 aactacagaccggtttcgcccttcttggggctcatcagtgtagtgcag 227
```

TmSat11

Name	From	To	Name	From	To	Dir	Sim	Pos/Mm:Ts	Score
TmSat11	264	330	Ginger1-5_HM	1109	1170	c	0.8413	99.0000	82

```
 330 aaacagaga---gcaataaa-atgtttttgattctaa-aataaccagatttgtttaaaaaatttcgtttg 266
     |||||| ||---||||||||-|||||||-------||-||||||-||||||||||| ||||--| | |||
1109 aaacagtgaatggcaataaatatgtttt-------aacaataac-agatttgtttacaaaa--tggattg 1168

 265 tt 264
     ||
1169 tt 1170
```

TmSat13

Name	From	To	Name	From	To	Dir	Sim	Pos/Mm:Ts	Score
TmSat13	79	121	Dada-U6_DaAe	4028	4079	d	0.8478	4.0000	68

```
  79 tgctgatggtatcatcgg---atca-----ttgttattttcttta-tttgtc 121
     |||||||:||||||| | ---||||-----|||||||||| ||||-||||||
4028 tgctgatagtatcatagctacatcactgttttgttattttatttattttgtc 4079
```

TmSat16

Name	From	To	Name	From	To	Dir	Sim	Pos/Mm:Ts	Score
TmSat16	78	450	Helitron-N10_LMi	399	804	c	0.7195	2.3714	256

```
 450 gatttgggctttcggttaaaaatgaagaaacacgtgttttaggatttttgaaaattccaccctctagggg 381
     ||| |||: |:|:|||:|::| |:||:||||||| ||||:|| |||||||:|||||| ||   : ||:||
 399 gatatggaatcttggtcaggattaaaaaaacacgagtttcagtatttttggaaattcaacaagtaagagg 468

 380 gttaaacaggg-atgacaatttgtatt--aaaa--------tat---gtcatttataaagttaaatcctt 325
     || |||:||||-|||| || || | ||--||||--------|||---| || || |:|||:||:| |--|
 469 gtaaaataggggatgaaaaattctttttgaaaacacgttgctattcagacaattttgaagctagagc--t 536

 324 g-aaaatttg-tatttaggctt-tcgattaaaaatcaagtaatacgtgtttcaggatttttggaaatttc 258
     |-|||| |||-|||||-|  ||-|:|:|||:|| |:||: ||||||||||||:| |||||||||||||:
 537 gcaaaaattgatattt-gtattgttggttagaatttaaaaaatacgtgtttcggtatttttggaaattca 605

 257 accctcaaggggggttgaa---ca-agggta-aaacttcgtaaaatatccgtt----ttt------tttt- 204
     ||| | |||||||||:||--- |-|||| |-:  :||||-|||--|||:|||----|||------||||-
 606 accataaaggggggttaaattggagaggggatgtttttcg-aaa--atctgttgctatttaggcaattttg 672

 203 aagttaaatctatgaaaattggtatttg-----ggtgttc-agttaaaaacaaaatagcacgtgtttcaa 140
     ||||||:| |||::||  || | |||||-----|| ||||-|:|| |||| |------||||||||||::
 673 aagttagaactacaaattttcggatttggattcggggttcgaatttaaaaga------cacgtgtttcgg 736

 139 ga-ttttggagaattctatccctaagggggtcaaataagggatg-aaa----g-tttgtataaatatgt 78
      |-|||||||-||||| |:|  ||||||||| ||||| ||||||-|||----|-||| :| ||||||||
 737 tatttttgga-aattcgaccaataaggggggtgaaatacgggatgcaaaaattgattttcaaaatatgt 804
```

TmSat17

Name	From	To	Name	From	To	Dir	Sim	Pos/Mm:Ts	Score
TmSat17	9	54	L1-68_TAe	1820	1866	d	0.8085	2.3333	70

```
   9 caagttactttactatagcccaaaaaatgac--actcttttaatatta 54
     |||-| || |||:||||||:|||:|||| ||--||||||||||| |||
1820 caa-taacattattatagctcaagaaattacccactcttttaatctta 1866
```

TmSat20

Name	From	To	Name	From	To	Dir	Sim	Pos/Mm:Ts	Score
TmSat20	85	122	Helitron-41_CGi	9976	10018	c	0.8780	2.0000	74

```
 122 tttgagtgcttacattg-tcagtgtta---ccaaatt-aagat 85
     ||||| |||||||||||-|:|||||||---|||||||-|||||
9976 tttgattgcttacattgattagtgttatagccaaattcaagat 10018
```

TmSat21

Name	From	To	Name	From	To	Dir	Sim	Pos/Mm:Ts	Score
TmSat21	144	240	Helitron-N9_AT	1755	1853	c	0.7872	3.0000	70

```
 240 cat-aattttttaataaa--c--tag-----atgttcgaataagagtacaaa----aaattgagtt--tt 187
     |||-||||:| |||||||--|--|||-----|||||::|||--||-||| ||----||||||||  --||
1755 catcaattctgtaataaattcaatagtatccatgtttaaat--ga-taccaataataaattgagaaactt 1821

 186 ttaataaatttattaattaataaatgatgatcaataaacattt 144
     ||||--||||||| |||||-|| |--------|||||||||||
1822 ttaa--aatttataaatta-taca--------aataaacattt 1853
```

Name	From	To	Name	From	To	Dir	Sim	Pos/Mm:Ts	Score
TmSat21	261	354	Athena-T1_As	21911	21986	c	0.7738	2.2000	74

```
  354 ttcttattaatttcaaatttaactctctagttacagtttttttcgtaattaaattagtgtctcgttagta 285
      ||| | ||||||| :|||||||||||:|--||--||||| |||-|: |:|||||--||-|||:|-----|
21911 ttcgtcttaatttagaatttaactcttt--tt--agtttgttt-gctactaaat--gt-tcttg-----a 21967

  284 ttatttatagtcataaaaaactta 261
      |||||| |||--||---|||||||
21968 ttatttttag--at---aaactta 21986
```

Name	From	To	Name	From	To	Dir	Sim	Pos/Mm:Ts	Score
TmSat21	360	434	Athena-I1_As	410	475	c	0.7971	4.0000	72

```
434 tgtggctca-gctgaattatttaaaaatgttc-agcttcgtctcttaaactcacttttactcctaataac 367
    ||| |||||-|||||||||||||||||---||-|:| ||:||  |||--------||||-|  |||||||
410 tgttgctcatgctgaattatttaaaaa---tcgaacatcatcaatta--------ttta-tgataataac 467

366 -ataatat 360
    -|||||||
468 aataatat 475
```

TmSat24

Name	From	To	Name	From	To	Dir	Sim	Pos/Mm:Ts	Score
TmSat24	275	447	MuDR-1_PaM	520	672	d	0.7333	2.1667	104

```
275 aattttcaagaaaagttta-ttttcat-tattt--ttacataattttagagctttctaaattatctatag 340
    |||||||||||||| ||||-|||| ||-:||||--||| |-||||||||---||----| ||||- :|||
520 aattttcaagaaaattttagttttgatacatttagttaga-aattttag---tt----atttat-ggtag 580

341 -ttttaattatttaataattggataacagcttgtcatttgattaatttt-gtggttttgctaggaaggtt 408
    -|||||:||| ||-|| |||||-|---|| ||-|:| ||::||||||||-||:| |||--||| ||--||
581 attttagttagtt-attattgg-t---agatt-ttagttagttaattttggtagattt--tagtaa--tt 640

409 attacaagtgacaaaattcaagccatttttgataaaatt 447
    -||-----|||- |:|||  ||::|||||||:||:||||
641 -tt-----tga-tagattgtagttatttttggtagaatt 672
```

TmSat25

Name	From	To	Name	From	To	Dir	Sim	Pos/Mm:Ts	Score
TmSat25	42	114	Crack-5_SP	4555	4624	c	0.7808	11.0000	88

```
 114 tttccaacatgcatgtaatatactatttca--taatttttttcgcaaaagttttaatattatttcttttg 47
     |||||| -|||-||||||||||--||||||--| ||||| |:|| ||| | || |||| ||| ||||||-
4555 tttccat-atg-atgtaatata--atttcatttcattttatccgaaaatgattaaataatatatctttt- 4619

  46 ttctg 42
      ||||
4620 atctg 4624
```

TmSat28

Name	From	To	Name	From	To	Dir	Sim	Pos/Mm:Ts	Score
TmSat28	91	116	I-1_DPe	3130	3156	d	0.9630	99.0000	70

```
  91 taccaacaacactttcttacc-tcaaa 116
     |||||||||||||||||||||-|||||
3130 taccaacaacactttcttaccatcaaa 3156
```

Name	From	To	Name	From	To	Dir	Sim	Pos/Mm:Ts	Score
TmSat28	320	372	Gypsy18-VV_LTR	1098	1153	c	0.8000	4.0000	74

```
 372 agaatattttcaaaaatttgag---aaa-attgagtaatattttggaaagtagagat 320
     ||||||||||||:||||||| ||---|||-| | ||| |-|||||||| | ||||:||
1098 agaatattttcgaaaattttagggtaaatagttagtta-attttggatattagaaat 1153
```

Name	From	To	Name	From	To	Dir	Sim	Pos/Mm:Ts	Score
TmSat28	378	417	hATm-1_AA	5357	5394	d	0.8500	99.0000	72

```
 378 ttactcaagtcccaaaaagggccatttttttcaaacacaaa 417
     |||||-|||||||-|||||| | |||||||||| | |||
5357 ttact-aagtccc-aaaaggtcgatttttttcaaaaaaaaa 5394
```

TmSat29

Name	From	To	Name	From	To	Dir	Sim	Pos/Mm:Ts	Score
TmSat29	1	40	Harbinger-13_CGi	2530	2570	d	0.8293	6.0000	70

```
   1 ttgca-aaattaagtcccaaaagcgttttttcgcctgtttt 40
     |||:|-|||||||||||||    |||||||||||||| |||
2530 ttgtacaaattaagtcccattcccgttttttcgcctgattt 2570
```

TmSat30

Name	From	To	Name	From	To	Dir	Sim	Pos/Mm:Ts	Score
TmSat30	280	332	Gypsy-3_PomCan-I	38	87	d	0.8302	5.0000	82

```
280 atttc-ccagacaactttccaggtttgaggactttctttctcatttttaaatat 332
    |||||-||||| |||||||-||||--|:| || ||-||||||||||| ||||||
 38 atttctccagaaaactttc-aggt--gggcacgtt-tttctcattttgaaatat 87
```

TmSat32

Name	From	To	Name	From	To	Dir	Sim	Pos/Mm:Ts	Score
TmSat32	112	285	EnSpm-3N1_RSa	755	892	c	0.7740	4.7500	96

```
285 tgtc-aaataccaaactccaaaactcattctgacagatgacataacacatcaaattttaaactattgaaa 217
    ||||-|||| |||||||||||| |:||||------|||--------------||||||| | | || |||
755 tgtctaaatcccaaactccaaaccccatt------gat--------------aattttataattttcaaa 804

216 agtcaaattatatttcc----gttcgaaaaaatt-tagtttttatttaaaaaacgtggttataaacattt 152
    ||----|||||||||:|---- |||-||||:|||-||-|||||:||  ||||-------|| |||-||||
805 ag----attatattttcaaatcttc-aaaagattata-tttttgttgcaaaa-------tagaaa-attt 860

151 aaaaatactaatataatattatttctgttttaatttgata 112
    |||-----|||-||||| | ||||-||-| ||| || |||
861 aaa-----taa-ataatttgattt-tg-tataagtttata 892
```

Name	From	To	Name	From	To	Dir	Sim	Pos/Mm:Ts	Score
TmSat32	294	371	Harbinger-8_CGi	5930	6005	c	0.7848	6.0000	70

```
 371 aaagaatttggtacttatattccat-acaatttttt-taa-ta---a--aaattacttcccgccacattt 310
     ||| | |||: ||||||||||-|||-| ||||||||-|||-||---|--|||||--|||---|||-||||
5930 aaatattttactacttatatt-cataagaatttttttgtaagtatttaccaaatt--ttc---cca-attt 5992

 309 ttgacacctgtcaaat 294
     ||||-|| ||--||||
5993 ttga-acatg--aaat 6005
```

TmSat33

Name	From	To	Name	From	To	Dir	Sim	Pos/Mm:Ts	Score
TmSat33	2	100	HARB-7_ALy	4647	4734	c	0.7553	2.6667	88

```
 100 tattttagaagtgattaattttaattgagatttatttgtgctctatcttattgattttattccaactcta 31
     ||||:|||||---||||| |||||---|-||--|||||||||:||| ||||| || ||||-- ||:| ||
4647 tattctagaa---attaagtttaa---a-at--atttgtgctttatgttatttatgttat--aaattgta 4705

  30 ccttcattta-gctctattttttgttattt 2
     :|-|||| ||-|:|:|| ||||| | ||||
4706 tc-tcatgtatgttttaatttttttaattt 4734
```

TmSat34

Name	From	To	Name	From	To	Dir	Sim	Pos/Mm:Ts	Score
TmSat34	11	80	Tx1-17_CGi	2958	3022	c	0.8182	4.5000	92

```
  80 aaatcttaaaaatacgatttttttgacata--aaatagaagacaaaaaccatgtataaccagttaaaata 13
     ||||||| ||||||---|||||||:|| ||--||| || |||||||||| | ||||||----|||||: |
2958 aaatcttcaaaata---tttttttaacttagaaaaaagtagacaaaaacaaagtataa----ttaaagaa 3020

  12 ct 11
     ||
3021 ct 3022
```

Name	From	To	Name	From	To	Dir	Sim	Pos/Mm:Ts	Score
TmSat34	130	189	KolobokP-10_CGi	1603	1661	d	0.7869	3.0000	78

```
 130 aaaaattggtctcagagcaatttactcgtt--tttttaggaatttcaaaaatgtaacaaaat 189
     ||||||||||:||||||||:||||-|-|||-- || :|  | |||||||||| |-|||||||
1603 aaaaattggtttcagagcagttta-t-gttaaattacattattttcaaaaatct-acaaaat 1661
```

TmSat36

Name	From	To	Name	From	To	Dir	Sim	Pos/Mm:Ts	Score
TmSat36	36	157	hAT-73_HM	219	341	d	0.7815	7.0000	100

```
 36 acaattc-tataagacaatttttttagtttttgttgattttaacattactt--------t----tgtttt 92
    ||||||:-| ||| |--||||||||--||||||||--||| || |||||||--------|----||||||
219 acaatttgtttaata--attttttt--tttttgtt--tttaaaaattacttaaagaaaataaagtgtttt 282

 93 ataagtttgaaa---ttaaggctaaaatttaattttttaagaaactccatttcataagtataaaaact 157
    |--|||||-|||---|| |---| | ||||||||||--||-| |:|  ||||||||||| || |||||
283 a--agttt-aaacttttca---tcatatttaatttt--aa-atattagatttcataagtttataaact 341
```

TmSat37

Name	From	To	Name	From	To	Dir	Sim	Pos/Mm:Ts	Score
TmSat37	38	130	Polinton-2_DTa	8473	8565	d	0.7579	2.6000	82

```
  38 ttggaa-aactttg-caaaacatatcgaattttttt--tatattttcttctaaataaaaataaacaacat 103
     ||||||-|| |||:-||||||-||:|:||||||||:--||-||| |||  || |||||||||||| ||--
8473 ttggaacaagtttaacaaaac-taccaaattttttcacta-attgtctaatacataaaaataaaccac-- 8538

 104 gtactaacgagttgt-aatta---aatttgt 130
     -:||| |-||--|||- ||||---|||||||
8539 -cactta-ga--tgtgcattaaataatttgt 8565
```

TmSat39

Name	From	To	Name	From	To	Dir	Sim	Pos/Mm:Ts	Score
TmSat39	27	97	Kolobok-7_CGi	2600	2674	c	0.8082	7.0000	86

```
  97 atttcagctaaaaacgcaattat--tttgcaaaattgcgttgaag-a-taagccaaa----aa-ttcaac 37
     ||||-|||||||||:|||| || --||| ||| ||||----||||-|-| || ||||----||-||||||
2600 attt-agctaaaaatgcaagtaactttccaatattg----gaagcactcaggcaaatgaaaacttcaac 2664

  36 aaaatagtgg 27
     ||||||||||
2665 aaaatagtgg 2674
```

Name	From	To	Name	From	To	Dir	Sim	Pos/Mm:Ts	Score
TmSat39	137	340	Gypsy-131_DTa-I	1167	1317	c	0.7590	3.6667	82

```
 340 ataaaaaaatttaaacaagttaaaaaattataaaattaaatcgaaagcccaaaaataaattcaaatagat 271
     ||||||||--| |||:||: ||||| |  ||||||| |||| -|||----|||||||||:| |||||:|-
1167 ataaaaaa--taaaataaaataaaataaaataaaataaaata-aaa----aaaaataaactaaaataaa- 1228

 270 gttatttcgtttatttcggtcgattaaaa-aaaatcactctgtatgtactactgaattagtgaattggta 202
     ----------------------|| ||||-||||| | -----||-||:||---|||||-----|||||-
1229 ----------------------ataaaaataaaataaa-----at-tatta---aatta-----ttggt- 1261

 201 atcccgattgataatttcgtcaattttc-ttaaagaaattgaaaactaccaatttggttg-aaaat 137
     -||:| -||||||||||||-||||||||-||--||--|||  |||| |--|||||--|||- ||||
1262 -tctct-ttgataatttcgt-aaattttcgtt--ag--attttaaacaa--aattt--ttgttaaat 1317
```

TmSat40

Name	From	To	Name	From	To	Dir	Sim	Pos/Mm:Ts	Score
TmSat40	58	86	MuDR-43_TAe	8544	8573	d	0.9333	1.0000	72

```
  58 gaaaaacaa-catgttttgcgcccaatttt 86
     |||||||||-|||||||||:||||||||||
8544 gaaaaacaaacatgttttgtgcccaatttt 8573
```

Name	From	To	Name	From	To	Dir	Sim	Pos/Mm:Ts	Score
TmSat40	227	269	Gypsy-17_NeMe-I	2051	2098	d	0.8667	99.0000	70

```
 227 gacaaat---tttccttaaaaaa--tta--cagatttgcaagcagatttg 269
     |||| ||---|||||--||||||--|||--|||| |||||||||||||||
2051 gacacatggctttcc--aaaaaaccttatgcagaattgcaagcagatttg 2098
```

TmSat43

Name	From	To	Name	From	To	Dir	Sim	Pos/Mm:Ts	Score
TmSat43	133	168	Tad1-21_PoXa	526	562	c	0.8889	2.0000	68

```
168 aaagacctcaacctaaaag---acctgaagataatcttc 133
    ||||--||||:||||||||---|||||||||||||| ||
526 aaag--ctcagcctaaaagcagacctgaagataatcgtc 562
```

TmSat44

Name	From	To	Name	From	To	Dir	Sim	Pos/Mm:Ts	Score
TmSat44	1	50	Gypsy-16_LSal-I	3227	3276	c	0.8039	2.0000	76

```
  50 tacttt-cttttctttgttaaataaggcaaaaaataggtcaagaattgttt 1
     ||||||-|| |||:|-|||||||||:| |:|||||| ||:|| ||||||||
3227 tactttgctgttcct-gttaaataaagaagaaaatatgttaacaattgttt 3276
```

TmSat45

Name	From	To	Name	From	To	Dir	Sim	Pos/Mm:Ts	Score
TmSat45	105	151	Daphne-9_LMi	1901	1948	c	0.7959	7.0000	68

```
 151 accaca-cgagaaagattgcaccaaat-tctacaaatgtgaccatggaa 105
     ||||||-| | ||   |||-|||||||-|:|||||||||| ||||||||
1901 accacagccacaatccttg-accaaatatttacaaatgtgcccatggaa 1948
```

Name	From	To	Name	From	To	Dir	Sim	Pos/Mm:Ts	Score
TmSat45	210	249	Kolobok-1_CGi	1861	1905	c	0.8571	2.0000	72

```
 249 gaaaagccaacaacttca-ggaact----ccaaaaccaactccac 210
     |||||||||| |||||||-||||||----|||||| |||:|:|||
1861 gaaaagccaaaaacttcagggaactcacaccaaaaacaatttcac 1905
```

TmSat47

Name	From	To	Name	From	To	Dir	Sim	Pos/Mm:Ts	Score
TmSat47	113	154	BEL-32_EnFl-I	1417	1458	d	0.8372	4.0000	68

```
 113 gattattaaattga--aatgtcacaattttatagtcacttgtca 154
     |||:|||  |||||--||||||||||||||||-| ||-||||||
1417 gatcattttattgacaaatgtcacaattttat-gaca-ttgtca 1458
```

Name	From	To	Name	From	To	Dir	Sim	Pos/Mm:Ts	Score
TmSat47	234	324	Gypsy-30_DPu-I	4013	4100	d	0.7614	2.5000	80

```
 234 tttttttgtcactactctacattattcaagttggcaacattgactatcacattca----a-atgtcacaa 298
     |||:|| ||||: || ||-|||:||||-------|| | |:|-||||||||||||----|-|| ||||||
4013 tttcttagtcataacgct-catcattc-------catcttcg-ctatcacattcatatcatatttcacaa 4073

 299 tttcatagttaac-tgtcatttatctc 324
     ||||||| || :|-|||| ||:|||||
4074 tttcatattttgcttgtccttcatctc 4100
```

TmSat49

Name	From	To	Name	From	To	Dir	Sim	Pos/Mm:Ts	Score
TmSat49	123	199	Keno-7_XT	2727	2800	c	0.7692	2.2500	72

```
 199 ctttta-ctc---tattcttgtga-catttagtcataaaagtggtcacttttgtgatattttatggtgaa 135
     ||||||-|| ---||||-|| |||-|||: |||: |||--||| ||--||:|-|||-||||||||||||
2727 cttttaactaatatatt-ttctgatcatcaagttttaa--gtgctc--ttct-tga-atttatggtgaa 2789

 134 aaaagagattaa 123
     |||||-|:||||
2790 aaaag-ggttaa 2800
```

Name	From	To	Name	From	To	Dir	Sim	Pos/Mm:Ts	Score
TmSat49	223	279	Sola2-4B_HM	4653	4709	c	0.7586	3.0000	70

```
 279 gtacaaaatgtcaaataatgtacctctcctg-aagcagcatgtcagggtaaaagtaaa 223
     ||||||||| |||:|-| |||| |  :| ||:-||||| |  |:| ||||||||||||||
4653 gtacaaaatttcaga-actgtagcattactacaagcaccttataagggtaaaagtaaa 4709
```

TmSat50

Name	From	To	Name	From	To	Dir	Sim	Pos/Mm:Ts	Score
TmSat50	32	158	Helitron-N7C_ALy	526	669	d	0.7500	2.6667	82

```
 32 ttttaatatttataatt--attccgattaccacttgtttttttaatt---tt-ttttacttgataaaa-a 94
    ||| ||||||||||-||--|||:|-||-||-|:||:|||||| || |---||-|||||: |: || ||-|
526 tttgaatatttata-ttcaatttc-at-ac-atttatttttttgaaatgtattattttatatactacaata 591

 95 atattttt------tttaacttt--aacaccttgtataatt--tttt--tt--tatt---c--gaatttt 145
    | ||||||------|||||-|||--||||---|:|| ||||--||||--||--||||---|--|||| |-
592 agattttttaaatattttaa-tttgcaaca---tataaaattagttttacttgatatttttcaagaatat- 656

146 cataaatatttaa 158
    ||| |||||||||
657 catcaatatttaa 669
```

TmSat52

Name	From	To	Name	From	To	Dir	Sim	Pos/Mm:Ts	Score
TmSat52	208	514	Helitron-1_ArHy	1795	2087	c	0.7176	4.0909	112

```
 514 gtta--aatttgtaa---gtaatagtacttaataaaagtcaaaag-tacgattacaatttacttaattaa 451
     ||||--||||| |||---||| |--||:||  |||||---|||| -||--||||:|||||--||||||||
1795 gttattaatttttaatgtgtatt--tatttttaaaa---aaaatcta--attataattt--ttaattaa 1855

 450 a--attaa--tta-aatg--aaatttgaaggttttaaat-tatttattttttaaatgtagataaccagg 389
     |--|||| --|||-||| --||||||:||---||:||||-||:||-|||||||:||| |---| ||--|
1856 agtattatgtttagaattttaaatttaaa---ttcaaatatactt-tttttttgaattt---ttac--gt 1916

 388 aacacaattt-acgcatacattgcttaa-aagtta---actttcgacgttccaa--aagc-aattaaatt 327
     || -||||||-||:-|||: ||--||||-|| |||---| |||  | |||---|--| ||-||||| |--
1917 aag-caatttgaca-atatttt--ttaagaatttatataatttataagtt---attatgctaattaca-- 1977

 326 taagttaattata-taaa---aataaaatt-t--gtataattaattac---aaataatttaaaattacta 267
     -||| --| ||||-||||---||||:||||-|--|| | ||||| -||---|||| |||||----|||||
1978 -aagg--agtataataaagtaaatagaattatcagtcttattaag-acgtgaaattattta----tacta 2039

 266 gtacttacttaattacgctaattaggcacaaaagtttgagaaaaagtatattaataaag 208
     -|--|||   || |--|-||||   |: :|||--||| ||||||||||---||||||||
2040 -t--ttaaaaaaat--g-taatactgtttaaa--ttttagaaaaagta---taataaag 2087
```

TmSat53

Name	From	To	Name	From	To	Dir	Sim	Pos/Mm:Ts	Score
TmSat53	55	137	L1-170_XL	1382	1465	c	0.7711	3.2500	84

```
 137 aatt-ataggtttaccatcggaagtttt----cttaatgtcttgagaatttttctcaatattttgaggtt 73
     ||||-| ||  |||||||||||||:|||----|||-|||||-|||  ||| | :|||||:||||||----
1382 aattgaaagcattaccatcggaagctttcacactt-atgtc-tgattattatgttcaatgttttga---- 1445

  72 tccta--gtggtttcttttt 55
     |||||--|| ||||: ||||
1446 tcctaccgttgttttgtttt 1465
```

TmSat54

Name	From	To	Name	From	To	Dir	Sim	Pos/Mm:Ts	Score
TmSat54	19	160	Mariner-13_OT	7644	7798	d	0.7230	4.5000	84

```
  19 atgacaatcgt---t-atttct-tttttt---ctggtttcctttc-taa--accaataaagtattaaata 77
     |||| |||:||---|-|||| |-||||||---|||||||||| ||-:||--||:||||||-||--|| ||
7644 atgaaaattgtcattcatttatattttttagactggtttcctatcgcaagtactaataaa-ta--aatta 7710

  78 atactttagaggtgtttcctttg-----tcttttttaattccactttttt-----ttacaatgcttttttt 137
     | ||||| | | |: || :||||-----  || |||| || ||-||||||-----|||| |-|| ||||
7711 agacttttgtgttagttgttttgaagaagattatttatttgca-ttttttaaaaattacta-gcatttta 7778

 138 ctctgttcttaatcaaatctctt 160
     ||: | ||||-||--||||| ||
7779 cttgggtctt-at--aatctgtt 7798
```

TmSat55

Name	From	To	Name	From	To	Dir	Sim	Pos/Mm:Ts	Score
TmSat55	140	204	Kiri-3_DSuz	2239	2303	d	0.7538	2.8000	72

```
 140 tttttttcc--acagcaataacccaaattaaaaatttgatgaatcatgaaaatggaatttttgtgtt 204
     ||:|||| |--|||| :|| ||  ||||  ||| | |:||||||:|--||||:||||||||||||||
2239 ttcttttacgtacagagattacaaaaatataaactataatgaatta--aaaacggaatttttgtgtt 2303
```

TmSat56

Name	From	To	Name	From	To	Dir	Sim	Pos/Mm:Ts	Score
TmSat56	96	150	Gypsy-21_ST-LTR	84	131	c	0.8235	1.6667	68

```
 150 tttttttaaagttatcttaaatctccaaat-gtctactgtaattttttttttacaat 96
     ||||||| |||||||||----|||--||||-||:|--||| |||||||||:|:|||
  84 tttttttcaagttatct----tct--aaatagttt--tgtcattttttttcataat 131
```

TmSat57

Name	From	To	Name	From	To	Dir	Sim	Pos/Mm:Ts	Score
TmSat57	132	242	Kolobok-20_HMa	2560	2658	d	0.7736	3.3333	84

```
 132 ttgtttctaattgagaaaaatgatgaggttttatgc-ttgttttgc-ga--c-gaat--aaaaacgtaaa 194
     ||||||-|||----||||:-|||||---||||| ||-|| ||||||-||--|-| ||--|||||||||||
2560 ttgttt-taa----gaaag-tgatg---ttttaggcatttttttgctgattcagcattaaaaaacgtaaa 2620

 195 atttatttctgtagttttatgcccgttttgcgacaattaaaataaaaa 242
     ||||-|||:| |--|||| |----||||--|-|:|||| ||||| |||
2621 attt-ttttttt--tttttt----gttt--c-ataattcaaatataaa 2658
```

Name	From	To	Name	From	To	Dir	Sim	Pos/Mm:Ts	Score
TmSat57	326	370	CR1-74_AAe	5019	5059	d	0.8333	6.0000	68

```
 326 ttgtgtttatttgacaagaaagacgacgaggttttatgcccgttt 370
     || |||| || ||| |||||----|| ||||||||||||||:|||
5019 ttctgttgatatgaaaagaa----gaggaggttttatgcccattt 5059
```

TmSat58

Name	From	To	Name	From	To	Dir	Sim	Pos/Mm:Ts	Score
TmSat58	50	81	ERV3-1_CLan-I	1756	1790	d	0.9091	2.0000	70

```
  50 ggaatcaaa---cctcaaccagttcccggatttga 81
     ||||||| :---|||||||||||||||||||||||
1756 ggaatcatggcccctcaaccagttcccggatttga 1790
```

TmSat60

Name	From	To	Name	From	To	Dir	Sim	Pos/Mm:Ts	Score
TmSat60	42	79	MuDR-17_CGi	6549	6585	d	0.8718	2.0000	72

```
  42 tatttcaactttgatgttttctttaatagtaaa-tgtag 79
     ||||||||||| -|||||||||||||-|:||||-|||||
6549 tatttcaactta-atgttttctttaa-aataaattgtag 6585
```

Name	From	To	Name	From	To	Dir	Sim	Pos/Mm:Ts	Score
TmSat60	86	118	DIRS-50_CGi	507	538	c	0.8788	1.0000	68

```
118 aaaacttgggttttgatggagacacaaattgat 86
    |||||||||||:||||||:|||-|||||||:||
507 aaaacttgggtcttgatgaaga-acaaattaat 538
```

Name	From	To	Name	From	To	Dir	Sim	Pos/Mm:Ts	Score
TmSat60	196	231	CACTA-1_PB	4580	4617	d	0.8919	1.5000	78

```
 196 atacattttttttt--aattaccttaacatattgtat 231
     |||||||||||||||--||||::||||||||||| |||
4580 atacattttttttttcaaattgtcttaacatattttat 4617
```

TmSat61

Name	From	To	Name	From	To	Dir	Sim	Pos/Mm:Ts	Score
TmSat61	163	217	hAT-8_AvMa	2529	2578	c	0.7925	1.3333	68

```
 217 ttaaaaaatgtttaatgactttgttgttgaatggagagaatttaactattaagaa 163
     ||:||:||-|||:|||||:|||||||||||--|||:|:|||| ||--| ||||||
2529 ttgaagaa-gttcaatgattttgttgttga--ggaaaaaattgaa--agtaagaa 2578
```

TmSat62

Name	From	To	Name	From	To	Dir	Sim	Pos/Mm:Ts	Score
TmSat62	151	293	EnSpm-N30_DTa	223	369	d	0.7571	2.8750	106

```
151 gaaataataatgttttcaaagtgataat--taaatgt-attttgataaatagtaaagatcaagaaattta 217
    |||||||: ||:|||-||||:--| |||--|||  ||-| |||||||   |   ||||||||||   |||:|
223 gaaataaccatattt-caaaa--aaaatggtaatggtcaatttgatatttttaaagatcaagttattca 289

218 tatcatttttttaacaagga-atcaatcaaaagataaaattaat-gttttttat--------c---aaaa 274
    |||||||||---------||-|| |||--|||||| | :|||||- ||||||||--------|---||||
290 tatcatttt---------gatatgaat--aaagattatgttaatcttttttttatattatttgctcaaaaa 348

275 atac--aatttaacatttttt 293
    ||||--|||||:::|||||||
349 atactaaatttggtatttttt 369
```

TmSat63

Name	From	To	Name	From	To	Dir	Sim	Pos/Mm:Ts	Score
TmSat63	177	225	ATCOPIA65_I	3094	3140	c	0.8298	99.0000	70

```
 225 taaatgacaccaa--ctcaaagagttgaagttcgaaccctgtctaaaacaa 177
     |||||||----||--||||||||||||||||||||||   ||||  ||| ||
3094 taaatga----aattctcaaagagttgaagttcgaaaaatgtcacaaagaa 3140
```

TmSat64

Name	From	To	Name	From	To	Dir	Sim	Pos/Mm:Ts	Score
TmSat64	115	263	Medea	5181	5358	c	0.7405	3.8571	94

```
 263 atcta-atttttgattt---tttgga--c-tgttttaacgaaattttgcaaag-aaattagtttttagct 202
     ||| |-||||||| |||---|||| |--|-||||||||  |: || || ||||-|||| |||||:|:|:|
5181 atcaacatttttgctttatttttgtaaacatgttttaaacagtttatgaaaagtaaataagtttctggtt 5250

 201 taaaaacgtactcaaatgctagaaaaag-------------g---ctaaaat---g--attatatttacg 153
     :||:|||| ||-|||||-|||| |||| -------------|---|| || |---|--||| | |||||-
5251 caagaacgaac-caaat-ctagtaaaacttccatattatttgtcactcaattttagcaatttttttac- 5317

 152 ttctaatatagcaatttttggtttattgt-----aaaaatta 115
     -|| |:|-|||||| ||||||||||||| |-----||| ||||
5318 -tcaagt-tagcaaatttttggtttattttcagcgaaatatta 5358
```

TmSat65

Name	From	To	Name	From	To	Dir	Sim	Pos/Mm:Ts	Score
TmSat65	199	244	MuDR-37_MT	1682	1722	d	0.8571	99.0000	68

```
 199 gcaaaatttgatcaatttctataaggtaacaatttgg-gaaaaaaat 244
     ||||||||||||| ||||------|||||||||| ||-||||  |||
1682 gcaaaatttgatctattt------ggtaacaattgggtgaaatcaat 1722
```

Name	From	To	Name	From	To	Dir	Sim	Pos/Mm:Ts	Score
TmSat65	293	352	Tx1-27_DR	1222	1283	c	0.7903	2.0000	74

```
 352 tttactcaccaa--a--ttttaacaaaatctgaaattatttgtttgagttgtaaattaatt-tct 293
     ||||:||-||||--|--||||:|||||||||| |:|: |||||||-- || ||||||||||-|||
1222 tttattc-ccaactactttttgacaaaatctgtagtcttttgttt--cttttaaattaattatct 1283
```

TmSat67

Name	From	To	Name	From	To	Dir	Sim	Pos/Mm:Ts	Score
TmSat67	113	151	Gypsy-55_DTa-LTR	1728	1768	c	0.8537	4.0000	74

```
 151 ggt-aattagaagtaaattgag-tgtattttccataataat 113
     |||-|||||||| |||||| | -||||||:|||||||||||
1728 ggtcaattagaattaaattcatatgtattctccataataat 1768
```

TmSat69

Name	From	To	Name	From	To	Dir	Sim	Pos/Mm:Ts	Score
TmSat69	150	204	L1-84_SpPu	2181	2242	d	0.8070	4.5000	78

```
 150 aagaaaaatca-ctgt------taaaagcacaaaataggaagaactagaaactggtatcaaa 204
     |||||||||||-||||------||  || |  ||| |||:||||:||| |||||||||||||
2181 aagaaaaatcagctgtcacttgtattagaaacaaagagggagaattagtaactggtatcaaa 2242
```

TmSat70

Name	From	To	Name	From	To	Dir	Sim	Pos/Mm:Ts	Score
TmSat70	47	122	Kolobok-N12_XT	322	390	d	0.8056	2.0000	86

```
 47 caaaaac-atcacaaacttgtaa-aaaagtaatattgtaaaatcaagggctgatt-caatttttcagttt 113
    |||||||-||:||||||||||||-||| ||-||||||:|||-------|||| ||- ||||||--:||||
322 caaaaaccattacaaacttgtaataaatgt-atattgcaaa-------gctgcttagaatttt--ggttt 381

114 tttttttta 122
    :|||| |||
382 cttttatta 390
```

TmSat71

Name	From	To	Name	From	To	Dir	Sim	Pos/Mm:Ts	Score
TmSat71	9	90	Helitron-N44_ALy	1801	1884	d	0.7558	4.6667	88

```
   9 ttaaaa-aaaacaaaaaaa--caccaaatt-gtcacccaagaaggaaaaaacatgcgtttcaaaattaca 74
     ||||||-|||| |||||| --|||: ||||-||-| | ||-||||||||| | | : ||||| ||||| :|
1801 ttaaaacaaaagaaaaaattccacttaattagt-agcaaa-aaggaaaatagaaaagtttctaaattcta 1868

  75 aa-ttgtcgttttagta 90
     ||-|||-||||||||||
1869 aagttg-cgttttagta 1884
```

Name	From	To	Name	From	To	Dir	Sim	Pos/Mm:Ts	Score
TmSat71	187	270	Copia-2_CoGl-I	1794	1867	c	0.8133	3.5000	76

```
 270 aaatagtctgtaaagtaactcaaaacagttggtaaaatagtacttttatag---act--tatattgtgag 206
     |||||| || ||||---|||:|| ||--||--||||||| |||||||||||---|||--||||||-----
1794 aaatagactttaaa---acttaatac--tt--taaaatattacttttatagataacttctatatt----- 1851

 205 gtaaaatagtat-ttaaaat 187
     ----|||| |||-|||:|||
1852 ----aatattatcttagaat 1867
```

Name	From	To	Name	From	To	Dir	Sim	Pos/Mm:Ts	Score
TmSat71	296	326	RTEX-12_SK	124	153	c	0.9032	99.0000	68

```
326 actgattttttgaacaatgattaagtatttt 296
    ||||||||||||||| |||-|||||| ||||
124 actgattttttgaactatg-ttaagtctttt 153
```

TmSat72

Name	From	To	Name	From	To	Dir	Sim	Pos/Mm:Ts	Score
TmSat72	4	117	Kolobok-N2_HM	967	1052	d	0.7917	4.5000	76

```
   4 aaaaattacaggattagtttttttaagggtttaagcacgtttttaagcattattttttttttagacagcaag 73
     |||||:--||| |||--||||||| ---|||||---||||||||||| |||-----|||||:|||  |||
 967 aaaaac--cagcatt--tttttttac---tttaa---cgtttttaagcctta-----ttttaaacatgaag 1021

  74 aatctatgaaaattc-atgtatgcttcaatccgtaaaatttttttt 117
     ----|||---|||||-|| |||--|--|| ||---||||||||||
1022 ----tat---aattcgatttat--t--aaacc---aaatttttttt 1052
```

Name	From	To	Name	From	To	Dir	Sim	Pos/Mm:Ts	Score
TmSat72	269	495	CryptonV-2_CTe	255	482	c	0.7412	2.1538	118

```
495 ataacaaaaacttgacgtttgattaatattaataatattattaatgaca--accagt-atctt-att--- 433
    ||||||  ||-|||||-|||||:||||--||||||:|||||||||||-|--||::||-|:|||-|||---
255 ataacattaa-ttgac-tttgactaat--taataacattattaatga-atgactggtcaccttcatttac 319

432 g-taatt-cgtttttaatttaattc--gtttctttgaatggtaaaaaactttccttatcctacttat-tt 368
    |-|||||-| |||  || |--||||--:||||----------|| |||-||||---||:||| ||||-|
320 gataattacctttgaaaat--attcaaatttc----------aacaaa-tttc---attctaattatgta 373

367 ggaaaaaatccgaacaacgacaataacttagtgt---aaca-tttg--c--gttaaataatttgttt--- 309
    ||| || ||--|-:||--||||:|:|--|:||:|---||||-||||--|--|||-- ||| :|||||---
374 ggataacat--g-gca--gacagtga--tggtatgacaacactttgatcatgtt--ttaagctgtttcaa 434

308 tttgattaat-t-tacacat---tttttaccaa--agt-ttcttattt 269
    ||||||||||-|-|||||||---||||| ||||--|||-|||| ||||
435 tttgattaatatatacacattaatttttttccaatcagtgttctaattt 482
```

Name	From	To	Name	From	To	Dir	Sim	Pos/Mm:Ts	Score
TmSat72	879	1172	Medea	6441	6746	c	0.7320	2.0385	166

```
1172 aaaatctcgcaaaaaaactgtgattttagttcaaaaacttgcttaaaact-tgaaaaaataaagtaacac 1104
     ||||||||||||||| |||||:||  |||||||||||| |||| ||||| -| ||||||--||-||| |
6441 aaaatctcgcaaaaacactgtaataatagttcaaaaacgtgctaaaaacgctcaaaaaa--aa-taaaaa 6507

1103 aattttcgaccaaattttatggtttttagcttgttttttgac--aaactttcactcaaaacgagtattaa 1036
     ||------||  ||:|||:|:-||||::||||:||:||-|||--|||--|| :||||||||:||: --||
6508 aa------acataactttgta-ttttcggcttattctt-gacgcaaa--ttggctcaaaacaagcc--aa 6565

1035 atcgaatgaaacaaacaaaatattatc---aatttaaa--tctaatttggtaatttttcagtcagtttt- 972
     ||:|||:||||:|: || |||-||| |---||||| : --|:|||----||||||||||||::|:||||-
6566 attgaacgaaatagtcataat-ttagcccaaattttgcggtttaa----gtaatttttcagctaatttta 6630

 971 ----------------aacaatactttg----tattttggaaa---ccgaaaaacta-taaaaatgcaaa 926
     ----------------||||| |:||||----|||| ::| ||---||:||||| ||-| ||||---|||
6631 aaaaaatttcaaaaaaaaacaaaatttgtgtctattacagcaatttccaaaaaaatactcaaaa---aaa 6697

 925 attaacacta-a----tttag-gtttattttg-gtaaatttttggtttgttttt 879
      ||||-||||-|----||| |-|:  ||||||-||---|||||-||:|||||||
6698 tttaa-actatacaattttcgagcaaattttgtgt---ttttt-gtctgttttt 6746
```

TmSat73

Name	From	To	Name	From	To	Dir	Sim	Pos/Mm:Ts	Score
TmSat73	40	181	Gypsy-103_NS-I	1041	1178	d	0.7286	4.5000	98

```
  40 aaaaagttgtttttgtttgtttcctttctagtaatacttgtttaaatta-a---tcaattttc--gaaaa 103
     ||||||| -||||| |||:||||||||||||-|| | || |||---|||-|---:||||||||--|||||
1041 aaaaagta-tttttctttatttcctttctag-aaaagtttttt---ttagaccccaattttcaagaaaa 1105

 104 attatgtcaaaaaacaagtattttcaagttgtaatatatt--gttattattggacatt-tattaagaaaa 170
     |--||-:||||||-----||||||| | |  |:||:| ||--|  | | ||    |||-| ||||:||||
1106 a--at-ccaaaaa-----tattttccattattgatgtctttagaaagtctttttaattgttttaaaaaaa 1167

 171 aacaattaaaa 181
     || || |||||
1168 aaaaaataaaa 1178
```

TmSat74

Name	From	To	Name	From	To	Dir	Sim	Pos/Mm:Ts	Score
TmSat74	322	364	L1-78_XT	1828	1869	d	0.8140	3.5000	70

```
 322 agactcaccttacatggaagcaaattaccccatttaaaacaca 364
     ||||||||-|| || |:||:|||| |||||||||| ||| |||
1828 agactcac-ttccaggaaaacaaaataccccatttaaaaaca 1869
```

Name	From	To	Name	From	To	Dir	Sim	Pos/Mm:Ts	Score
TmSat74	476	541	DasNov-4.624	4177	4237	c	0.8125	9.0000	94

```
 541 ttctacactttttgtttaccctgtatgtgtttttcaagagtcgtctatttcaataaagtttaaaaa 476
     || ||||||| |||||||---||||||| | | |-| |||| :| ||||||||||||-||||||||
4177 ttatacacttattgttta---tgtatgtttatgt-atgagtgatatatttcaataaa-tttaaaaa 4237
```

Name	From	To	Name	From	To	Dir	Sim	Pos/Mm:Ts	Score
TmSat74	549	740	Mariner-38_OT	8222	8432	c	0.7222	4.4286	78

```
 740 ttaaacaattcataaaa--aaac--ac-tattg--a-tttaaaaacaattttattt------------gt 691
     |||||:|||  ||||||--||||--||-|||| --|-||||||||:||||||||||------------||
8222 ttaaataataaataaaacaaaacttacgtattttagtttaaaaataattttatttacaatcttatttgt 8291

 690 tggattgttagatcatagtctttgatcaaaaaattggttat--t--ataagttataaa----aaatggtg 629
     ||:|||:||-|--|----|:|| ||  ||||||||-- |||--|--|||| |||-|||----|:||--||
8292 tgaattatt-g--c----ttttagagaaaaaaatt--atatcatccataattta-aaatcttagat--tg 8349

 628 aacttaaaaaataaaaagtattttga-a----aaaatattagtgaaaa--caatcg-tatttt-cataat 568
     |||| :   ||| ||   |||||||-|----||||  ||--|||| |--|||  |-||||||-||-||-
8350 aactwgtttaattaactttattttgagagaggaaaagttt--tgaatattcaaaagttattttgca-aa- 8415

 567 acttagtttagataaaata 549
     |||||--||| ||| | ||
8416 actta--ttatatacacta 8432
```

TmSat75

Name	From	To	Name	From	To	Dir	Sim	Pos/Mm:Ts	Score
TmSat75	357	394	P-41_HM	3012	3052	c	0.8500	99.0000	70

```
 394 ttttttttgtaat--at-aaaaaatgtgatcgtattttaa 357
     |||||||| |||||--||-||||||||| ||| |||| |||
3012 tttttttagtaatcgattaaaaaatgttatcttattgtaa 3052
```

TmSat76

Name	From	To	Name	From	To	Dir	Sim	Pos/Mm:Ts	Score
TmSat76	80	136	Gypsy-3_DSer-I	6885	6942	d	0.7931	8.0000	72

```
  80 tattccttaattaatttaggactttc-ctatttcac-tcaaattg--aaacatgaattaat 136
     ||||:| |||||||||||  |||||- ||||||||-|---|| |--| |||||||| |||
6885 tatttcataattaatttagttctttctatatttcacgt---atggccatacatgaataaat 6942
```

Name	From	To	Name	From	To	Dir	Sim	Pos/Mm:Ts	Score
TmSat76	212	544	ISL2EU-N3_BTa	1005	1295	d	0.7138	3.8000	96

```
 212 aaaaac---aagcataattacaaaatcatttgcaaaaaaactacaaaacaccatacgaaaataaaataaa 278
     ||||||---||--| |||||-|||||-|||-|:||||||--|  ||||-----------||||||| |||
1005 aaaaacttgaa--aaaatta-aaaat-att-gtaaaaaa--ttgaaaa-----------aataaaaaaaa 1056

 279 attttgcaaaagaaaattaaaaagtgttaagtttttaataa---gacataataacaaaagaacattaaaa 345
     ||----|||| :||||||||||----||---|| ||| ||---|| | ||||| ||||-| ||   |||
1057 at----caaataaaaattaaaaa----ta---ttgtaaaaaattgaaaaaataaaaaaa-atcaaataaa 1114

 346 aaataaagttctgaat-ttgaacctcgaaaagttagaactgaaatcggaattttataaattggaattaga 414
     || ||||------|||-|||||------|||-----||:|||||----|| || ||||| | :||| |:|
1115 aattaaa------aatattgaa------aaa-----aattgaaa----aaattaataaaatcaaataaaa 1163

 415 gtttcaaaagtttcagttcacaaatttgtcttaaaacagtcagaaaacaaggat--attttgaacgaaa 482
     :||  |||-----||||----||| | :| |||||| |:-||-||| :||| ||--||| |||:| | |
1164 attagaaa-----cagt----aaaataatattaaaaaaa-ca-aaattaagtatcaattattggaggcat 1222

 483 gtttctg-------aattatgacttttca--gatctgctaatacatac--ata--cttagcaataatgtt 539
      ||| | -------|||| |-|:| || |--| |-||  |||| :||:--|||--|||||||||:|:||
1223 ctttattttaaaataatttt-attattaattgtt-tgaaaataagtattaataaacttagcaatagtatt 1290

 540 tttgc 544
     |||||
1291 tttgc 1295
```

TmSat77

Name	From	To	Name	From	To	Dir	Sim	Pos/Mm:Ts	Score
TmSat77	1	81	Sola3-2_NV	1035	1115	c	0.7561	3.2000	90

```
  81 ttattttagcttgttttgctcgatttagtttgtt--ttttaaatagagacttatttattttgccaaaat- 15
     ||||||||| ||||||--||:: ||||  || ||--||:|||| |:|:|| |||| ||  |||||||||-
1035 ttattttagattgttt--cttattttatgttcttcattctaaaaaaaaacatattaatgatgccaaaatt 1102

  14 atgattaaaaatta 1
     || -||||||||||
1103 att-ttaaaaatta 1115
```

Name	From	To	Name	From	To	Dir	Sim	Pos/Mm:Ts	Score
TmSat77	290	624	Medea	6512	6810	d	0.7255	2.7368	126

```
 290 ataatttt---ttttcaatttaaatct---cgcagatctggcttaaaa-aggcaaaaat-aac-acattt 350
     ||||:|||---|||||:::||| -|||---||||:||-|||||:||||-|:|| ||| |-|||-| || -
6512 ataactttgtattttcggcttat-tcttgacgcaaat-tggctcaaaacaagccaaattgaacgaaata- 6578

 351 ttggtc-taatt----ctataattttt---tttgacttatgtttaacgaaattttacaaataggaacgtt 412
     ---|||-|||||----|:|-|||||| ---|||:| | || ||| |   |||||||-|||----||----
6579 ---gtcataatttagccca-aattttgcggtttaagtaattttcagctaattta-aaa----aa---- 6635

 413 attacctgaaaaacatgctaaaacactttttttttgttt-tattttagcaaaatctcgaaaaaaactgag 481
     ||| |  :|||||||-----|||--------|||||| |-|||| :|||||--|:|| |||||||-----
6636 atttcaaaaaaaaca-----aaa--------ttttgtgtctattacagcaa--tttccaaaaaaa----- 6685

 482 attttcgttcactataaagacttgaaaaggtcaagtaacaccattttcgaccaaattttgtgtttttcg- 550
     ---| |--|||----|||:| || |||----| |-||-||--|||||||| |||||||||||||||||:|-
6686 ---tac--tca----aaaaaatttaaa----cta-ta-ca--atttcgagcaaattttgtgtttttttgt 6738

 551 --gttaatattttgccaaaatcttactcaaaatga-gctaaatccaa---caaaataacattaaattaga 614
     --|||----|||||:||||| :|||:||||||  |-|||||||-|||---| || | |  :||| ||:|-
6739 ctgtt----ttttgtcaaaagtttattcaaaaacaagctaaat-caagctccaatttagtctaatttgg- 6802

 615 cttatttttt 624
     --||||||||
6803 --tatttttt 6810
```

TmSat78

Name	From	To	Name	From	To	Dir	Sim	Pos/Mm:Ts	Score
TmSat78	87	370	MuDR-N16_RSa	285	530	c	0.7050	5.3000	108

```
370 aaaagagcaacaacacacataa--attttaatgaatttttagcataaaa--aatgagtcaaaaaaatatt 305
    ||||:| ||-|||||---||||--|||||| |---|| |||--||||||--|||:|:  | ||||||||
285 aaaaaatca-caaca---ataattattttatt---ttatta--ataaaataaataaaaaataaaaatata 345

304 aaaaaaattactaaaatatgaagataaacaaattattgctgtgttcacataacgcaaaataaa----tac 239
    | || ||| |--||| |-||||:|-|| ||||  |||--------||:| ||  |||||||||----||
346 ataataataa--aaact-tgaaaa-aatcaaaaaatt--------cataaaaatcaaaataaaaaaataa 403

238 atttagataatgattcacttcattgtggagcagaatactttaagctaacaatccacaagaat-aatcta 170
    |||: |||||||||||---||:||| |--| :|||||-|--|| |---| |||-||  |||||-|||-|-
404 attcaagataatgatt---tttatttt--attagaat-c--tacg---agaat-cagtagaatcaat-t- 459

169 acaataattcaggtgcgggatagaataagacataaatattctaaattaatttagtttaaatatcaatagt 100
    |||| || |||----|-----|:|||||    |  || || | ||| || | |:| ||||-|| ||||
460 acaaaaaatca----c-----aaaataattattttattttattaataaaataaatataaa-ataaatata 519

 99 taaaaaagttaat 87
    |||||||--||||
520 taaaaaa--taat 530
```

Name	From	To	Name	From	To	Dir	Sim	Pos/Mm:Ts	Score
TmSat78	19	73	MuDR-5_SeTo	4105	4162	d	0.7759	5.0000	74

```
  19 taat-taaattacgaatattttttatttt-ctggattaaatat-caaaaactagacttt 73
     ||||-| | ||||||||||||||||||||-|| :  |||||||-|| ||| || |:|||
4105 taatattatttacgaatattttttatttacttacataaatatccacaaaatatatttt 4162
```

TmSat79

Name	From	To	Name	From	To	Dir	Sim	Pos/Mm:Ts	Score
TmSat79	11	42	EnSpm-28_HM	7403	7432	c	0.9062	99.0000	68

```
  42 taagacaactttgaaactaaaatttaaaaaaa 11
     ||| |-||||||||||-||||||||||||||
7403 taata-aactttgaaa-taaaatttaaaaaaa 7432
```

Name	From	To	Name	From	To	Dir	Sim	Pos/Mm:Ts	Score
TmSat79	106	341	CryptonH-7_HM	3962	4198	d	0.7186	3.8182	100

```
 106 atttt---gtagatttt---tttttttaatttcaaagctgtcttagttgatataattttattgtttgcgc 169
     |||||---|||| ||||---|| |||| ||| |||----||--|||||| || ||||||||||------|
3962 atttctagtagtttttatgttatttttattgcaa----gt--tagttgttaaaattttattg------c 4019

 170 actcgtgcttcgtcctcgcgta--ctaaagtgttaatt--ttat--agttattga-caatt---ttt--t 227
     | |-----||::   | ---||--| |||   |||| |--||||--|||  || |-|||||---|||--|
4020 aat-----tttaaaata---tatgcaaaatatttaaatagttatccagtattttatcaattctgtttact 4081

 228 ttaatt-ccaatatcaaatttggtataaaagtttt------gttaatt--ttataga-ttttt-gtaatt 286
     ||:|||-|||| :|||||||-||-|-|||||||||------|||| ||--|| | ||-|||||-|  | |
4082 ttgattgccaaggtcaaatt-gg-a-aaaagttttattcaagttatttagttttcgactttttcgacaat 4148

 287 tttttttttcatttcaatatcaaagttgtcaaagttgggaagttggtatataaaa 341
     ||||||||| :|||:  |:|:|:  ||||||||:||-:||||||----|||||||
4149 tttttttttagtttttttgttagctttgtcaaaatt-agaagtt----tataaaa 4198
```

TmSat80

Name	From	To	Name	From	To	Dir	Sim	Pos/Mm:Ts	Score
TmSat80	9	45	TE-X-11_DR	1360	1401	c	0.8974	2.0000	76

```
  45 attattttacaatt--aaaaattgtcgag---aaaatcgtca 9
     ||||||||||||||--|||||||||| |||---||||:|||||
1360 attattttacaatttaaaaaattgtggagtgtaaaaccgtca 1401
```

Name	From	To	Name	From	To	Dir	Sim	Pos/Mm:Ts	Score
TmSat80	385	439	EnSpm-N3_OS	415	477	d	0.7903	3.0000	72

```
385 tttttc-ctttttttaaac-ggttttatatgttt-ctgta-aa--taattgct-ta-aaaatgc 439
    || |||-|||||||::||-|||||||||||||||-| |||-||--||||  ||-||-|||||||
415 ttattctctttttttggactggttttatatgtttgcggtacaatttaatgtctctagaaaatgc 477
```

Name	From	To	Name	From	To	Dir	Sim	Pos/Mm:Ts	Score
TmSat80	139	361	Medea	6315	6552	c	0.7130	3.5385	100

```
 361 gaaatttcgcagaatatttgcatttctgcctgaaaaacgtccgaaaa----aaacaaattg--ttaacat 298
     |||||||:||:||||| :||||||||||:||| ||||||| | ||||----||| ||| :|--  ||||
6315 gaaattttgcggaataactgcatttctgtctgtaaaacgtgccaaaacaataaaaaaaacgcaaaaacaa 6384

 297 ttt--tta--atttt-----aaagtcattttgtatacaatttttagtct-ttttag-----tgt----- 248
     | |--:||--||| |-----|||||-||||||-||||: ||| ||| | |-|| |||-----| |-----
6385 tgtacctactattatcgacaaaagt-attttt-tatattattattattatgttatagcaaaatctcgcaa 6452

 247 ------tttaatttatcttaaaaaaacgtgtttaaaa-ggtcgaaaaagagaacattaacaacattttt 185
     ------| |||-| ||  |  |||||||||:| ||||-| ||:|||||:| ||-|--|| |||||-----
6453 aaacactgtaa-taatagttcaaaaacgtgctaaaaacgctcaaaaaaaataa-a--aaaaacat----- 6513

 184 caactaaatttggtaattttgtattatttttgaaaaaatttgtct 139
     -||||---||--||-|||||:|  |||||:||||  : || ||| ||
6514 -aact---tt--gt-attttcggcttattcttgacgcaaattggct 6552
```

TmSat83

Name	From	To	Name	From	To	Dir	Sim	Pos/Mm:Ts	Score
TmSat83	60	96	KolobokD-11_MyEd	10397	10436	c	0.8462	2.0000	70

```
   96 atttaataaaga-aca--actaattgtctttattcttctt 60
      | ||||||||:|-|||--||: ||||||||||||||||||
10397 awttaataaaaacacagcacctattgtctttattcttctt 10436
```

TmSat86

Name	From	To	Name	From	To	Dir	Sim	Pos/Mm:Ts	Score
TmSat86	46	192	Gypsy-5N_PPa-LTR	186	323	d	0.7343	3.0000	94

```
 46 tgtccattttaaagagctctcaatagaaaaaatagta---taagaaacac-gtgctttttagtttcttg 111
    || |:||||||||-|---||||| |||||||||||||---| ||||:  |-:|| ||||||--|||||||
186 tgactattttaaa-a---ctcaaaagaaaaaatagtaaatttagaaggtctatgatttttt--tttcttg 249

112 gagtggcac-taaaatcattttttt--ttctcaaa-taaagttaagttaataaaattagtattactt-ta 176
    :| |---||-| ||||| |||||||--|  | |||-|| |:||---|||:||:||||--| |--|||-||
250 aatt---actttaaatcttttttttagtgataaaactacaatt---ttagtagaatt--tct--cttcta 309

177 ccattctaacaaatta 192
    --||||| | :|||||
310 --attcttaggaatta 323
```

Name	From	To	Name	From	To	Dir	Sim	Pos/Mm:Ts	Score
TmSat86	303	332	Polinton-4_SM	7301	7332	c	0.9062	99.0000	68

```
 332 aataatt-ta-atcttgtaataaatcaaggtt 303
     |||||||-||-||||| |||||||||||||||
7301 aataattctagatcttttaataaatcaaggtt 7332
```

Name	From	To	Name	From	To	Dir	Sim	Pos/Mm:Ts	Score
TmSat86	345	399	Helitron-13_ZM	6889	6938	d	0.8148	4.0000	68

```
 345 ttggaaatgactgacaatttcctataaaagtgtgacgtt-tg-tatttgacaatcaa 399
     |||||||-|| -||||||||||||| |||||---|:|||-||-||--||||||| ||
6889 ttggaaa-gaa-gacaatttcctattaaagt---atgttatgcta--tgacaataaa 6938
```

TmSat87

Name	From	To	Name	From	To	Dir	Sim	Pos/Mm:Ts	Score
TmSat87	29	92	hATm-41_HM	366	424	c	0.7969	1.2500	70

```
 92 aactgttaatcgtagccgaat-aa-tttttacggaat-aatatttcttgattcattttacatcagaa 29
    ||:|:|||||--||-|:||||-||-|||||---||||-||:||||||| ||||||||||-||-||||
366 aattattaat--ta-ctgaattaacttttt---gaattaacatttcttcattcatttta-at-agaa 424
```

Name	From	To	Name	From	To	Dir	Sim	Pos/Mm:Ts	Score
TmSat87	188	231	PABL_AI	1307	1353	c	0.8261	6.0000	74

```
 231 taaataatttaacagaaaaaa-taa--actcgacgaatgattttca 188
     |||||| |||||||||||||||- ||--||| | |:|||| ||||||
1307 taaatactttaacagaaaaaaggaaagactagtcaaatgcttttca 1353
```

TmSat88

Name	From	To	Name	From	To	Dir	Sim	Pos/Mm:Ts	Score
TmSat88	153	371	Helitron-N42_ALy	582	806	d	0.7169	3.2500	96

```
153 tatttgtaataatattagtttta-attaataatacag----agtgttccagcttgctaatgtttg-tttt 216
    |||||: |||||||||| |||||-||||| || |  |----|:||||--|--| |: ||||| ||-|| |
582 tatttaaaataatattatttttagattaaaaaaaatggttaaatgtt--a--tagtaaatgtatgattgt 647

217 aattgtttttgcaataaaaaaggaatgaatt--tgggaataaaactcgaa---cacatgtca----agta 277
    ||  |||||| :|| | | |||:|||-||||--|---| | ||| |:|||---:|:|| |||----||||
648 aaaagtttttttaagatataagaaat-aattcat---atttaaaattgaatattatatttcaagagagta 713

278 a----ataaattcgaagcat---cgaattaaattcgttttaaccaattaatttaattatcttagt-ttaa 339
    |----||||||||-|--|||---||||--|||---- | |||- ||||||||--- |||||||:|-|||-
714 atagtataaattc-a--cattgacgaa--aaa----atataa-aaattaatt---gtatcttaatatta- 769

340 caacaatgg--aagta-aac---aaaaataaaaaattt 371
    -||: ||:|--||:||-|||---|||||||| ||| ||
770 -aattatagtaaaatataacaataaaaataataaagtt 806
```

Name	From	To	Name	From	To	Dir	Sim	Pos/Mm:Ts	Score
TmSat88	381	447	Helitron-1_DVir	586	651	c	0.8209	1.3333	78

```
447 acactaa-ata-acatgtggtaataatttcatcaaaatcggtt--gat---tagatctcgagttattaaa 385
    ||| |||-|||-||-|||:-|||-|||||||||||:|||||||--|||---||||-----||||||||||
586 acaataagatatac-tgta-taa-aatttcatcaagatcggttaagatacataga-----agttattaaa 647

384 aaaa 381
    :|||
648 gaaa 651
```

Name	From	To	Name	From	To	Dir	Sim	Pos/Mm:Ts	Score
TmSat88	494	579	hAT-125_HM	2095	2181	d	0.7674	2.1667	80

```
 494 cattttt-tactaatttttaataaatc--agtttttttttaattt---gctctccgatcaattgtttttg 557
     |||||||-||--|| | ||| |: |:|--||| |||:||:|||||---|||||::|||---|| ||||||
2095 catttttgta--aaatattattgtaccctagtatttcttcaattttgagctctttgat---ttttttttg 2159

 558 aggcatt--aaataaaaagttgtt 579
     |--||||--|||||||||| ||||
2160 a--cattttaaataaaaaggtgtt 2181
```

TmSat89

Name	From	To	Name	From	To	Dir	Sim	Pos/Mm:Ts	Score
TmSat89	30	109	Polinton-5_HM	3956	4029	d	0.7600	3.0000	76

```
  30 tttctaatatattattttgctaaaataggccaaaaaa-tcacttttttgtaaaaaaattgtgtaaaaata 98
     |||:|||||||||| |||||||||| |:   ||||||-|:||--------|||||||||:|   || |||
3956 tttttaatatattagtttgctaaaaaaataaaaaaaagttac--------aaaaaaattattgcaatata 4017

  99 gaaagtg-aatt 109
     :|| |||-||||
4018 aaatgtgtaatt 4029
```

TmSat90

Name	From	To	Name	From	To	Dir	Sim	Pos/Mm:Ts	Score
TmSat90	17	47	Mariner-52_HSal	163	193	c	0.9032	1.5000	72

```
 47 atttttttaaatagaacaccctgtattttat 17
    |||||||||||||||||||||::| ||||||
163 atttttttaaatagaacaccccattttttat 193
```

Name	From	To	Name	From	To	Dir	Sim	Pos/Mm:Ts	Score
TmSat90	97	190	Tad1-8_PoXa	1342	1430	d	0.7527	2.6000	74

```
  97 aattct-tgaaatgttggctaaatgaagctctatgacgctgcaa---aaaccgt-tttgtgctatctttt 161
     |||| |-||||||:|-|||:|  : |||||--||--|-||||||---||||| |-|||||||||||| ||
1342 aattgtctgaaatat-ggccactctaagct--at--c-ctgcaagagaaaccctatttgtgctatctgtt 1405

 162 caaataagggcacaatttcactt-tttaca 190
     | ||-|||----| ||:|:||||-||||||
1406 ccaa-aag----ccatcttacttctttaca 1430
```

Name	From	To	Name	From	To	Dir	Sim	Pos/Mm:Ts	Score
TmSat90	215	277	I-56_AAe	6205	6263	c	0.7812	2.0000	76

```
 277 aaaaatgatgtcaa-ttgtggaaaaaatttaacttctgtgatgctaatgcaaaaccaaatttgt 215
     ||||||-|-|||||-||:| || ||:||||:|-|| |:|-| ||||||||||||||-|||||||
6205 aaaaat-a-gtcaacttattgataagatttga-ttgtat-aggctaatgcaaaacc-aatttgt 6263
```

TmSat91

Name	From	To	Name	From	To	Dir	Sim	Pos/Mm:Ts	Score
TmSat91	155	218	EnSpm-20_CGi	3901	3972	d	0.7941	8.0000	76

```
 155 atttttgttataaaagtttc-attttaaccaaaaaatca-taaaa-----at-gt--tgattttttgata 214
     ||||||||||||||| || |-| ||||||--|||| |||-||| |-----||-||--|||||||| :|||
3901 atttttgttataaaatttactaatttaac--aaaattcactaatattctcatagttatgatttttaaata 3968

 215 tgat 218
     | ||
3969 tcat 3972
```

TmSat93

Name	From	To	Name	From	To	Dir	Sim	Pos/Mm:Ts	Score
TmSat93	16	567	MuDR-8_VV	9649	10222	d	0.6827	4.9545	96

```
  16 taaataaaaaattattaaaattataa---aaa--------att--cgaatata--agttatttc--t--- 65
     ||||-|||||| -||-||||||||||---|||--------|||--| | ||||--|-|||||||--|---
9649 taaa-aaaaaaw-at-aaaattataattgaaatctcagttattaactactatayta-ttatttcgataaa 9714

  66 gttgaaatta-c-taa---aaattagtagtagtagtgataatttttaattatttaaagactttctattaa 130
     | |:|| |||-|-|||---|||| |--| ||:||-|  |||   ||||: | ||||| |: ||  |||||
9715 gataaatttatcataatataaataa--attaata-tyttaaaaattaacaaattaaatatattaaattaa 9781

 131 ta--t-------ttttctat-ttctttctttgacatat---tttaggcatgacaaagactatt--ttta- 184
     ||--|-------|||| |||-|| ||| | || :||||---||||- :||- : ||||:||||--||||-
9782 taawttatacaattttatatattatttatatgttatataagttta-ttat-tttaagattattaatttat 9849

 185 gaatta-------attcttttctacgagattttgctctaataaatataaacattt-t-tttaattaacta 245
      ||| |-------|||| ||| ||--| ||:|---|||| |---||| ||-||||-|-|||: | || ||
9850 taataaatcaatattcatttata--atatct---tctatt---tatcaa-atttatgtttgttgaaata 9910

 246 a-atta-atttt--g-ttaaatat--ttaattccttaaaaaagta-a----gacaaattt-aataactc 302
     |-|:||-||||||--|-||||||||-- |||||: |||   |||||-|---- |-||||||-||| |-|
9911 atactagattttaagtttaaatataaataatttattatttaagtatattttta-aaatttcaatca-ta 9978

 303 cagtgt--ttt-atagtgttgaaataaacttta---tacaaaaggaaacaaaatca-aatttaaaaaa 365
      | |||--|||-||||| || || ||||:|  |---|| |||||--|||:|||||:|-|||| |||
9979 aattgtsatttaatagttttaattaaattkgaaagtaaaaaa--aaataaaattataattgaaatctc 10046

  366 aattgtgtgcacta-aaaatgattatgt--at-aacaaaaattaaaaaatcgagttttataaagtcgaaa 431
      |:||:|-|- ||||- | |  ||||| |--||-|| | |||||---- |||-|-|  ||||||-|--|||
10047 agttat-t-aactactataytattatttygataaagataaatt----tatc-a-taatataaa-t--aaa 10105

  432 ttaactatacagggtgttaaaaa--aagattgtaaattttttgaaggctttcacactttgattttttt 499
      ||||-|||-|------|||||||--|| |   ||||| | ||--||--- || |:|  ||-||-------
10106 ttaa-tat-c------ttaaaaattaacaaattaaatatmtt--aa---attaataawtt-at------- 10154

  500 gaaaattcgttttgttttctttta--ctatat-tttttttatttctaaatt-ttaaat--ttaac 563
      -| |||---||| |:| || ||| ||--:|||||-  ||| ||||||--|||:|||-|||| |--||||:
10155 -acaat---tttatatattatttatatgttatataartttattattt--taagattattaatttattaat 10218

  564 aaat 567
      ||||
10219 aaat 10222
```

TmSat94

Name	From	To	Name	From	To	Dir	Sim	Pos/Mm:Ts	Score
TmSat94	102	159	Helitron-N21_ALy	15057	15106	d	0.8364	2.0000	70

```
  102 gatgtgcctttgcatgct-taaaaattgacaacaagtgataaaaatgagcaaaatt-aca 159
      |||| |--||||---|||-|||||||-||:|||||---|-|||||||||||||||-|||
15057 gatgag--tttg---gctataaaaat-gataacaa---a-aaaaatgagcaaaattgaca 15106
```

TmSat96

Name	From	To	Name	From	To	Dir	Sim	Pos/Mm:Ts	Score
TmSat96	73	109	Tad1-67_PoXa	3771	3809	c	0.8462	99.0000	68

```
 109 aacaccaaaaaa-ctacaagaaaattgctgtt-tctcag 73
     ||||| ||||| -||||||||||||||| ||-||| ||
3771 aacacgaaaaacgctacaagaaaattgctcttctctaag 3809
```

TmSat98

Name	From	To	Name	From	To	Dir	Sim	Pos/Mm:Ts	Score
TmSat98	205	270	Merlin9_SM	951	1009	d	0.7937	2.0000	74

```
205 ctactttgtttttatacagagtgtcctttttttaataaatttg-cgaaaaatctaattttgggtcgt 270
    |||||||||||||| |:|:|||||--||| ||| ||-:|||||-|| |||----||||||:||-|||
951 ctactttgtttttaaataaagtgt--tttattttat-gatttgtcgcaaa----aattttagg-cgt 1009
```

TmSat103

Name	From	To	Name	From	To	Dir	Sim	Pos/Mm:Ts	Score
TmSat103	13	62	Gypsy-9_RC-LTR	874	921	d	0.7959	4.5000	72

```
 13 tatatttttcatgatttttgaagtttgaatgttgttggaaaaaatatgt 62
    ||| || ||  ||||||||||||:|||||||| | |||--|:||| ||||
874 tatcttgttgaatgatttttgaaatttgaatgatcttg--agaaagatgt 921
```

TmSat104

Name	From	To	Name	From	To	Dir	Sim	Pos/Mm:Ts	Score
TmSat104	18	65	KolobokE-3_MyEd	10299	10352	c	0.8113	2.5000	72

```
   65 aatttaagagat-g--taaca-acacag-a-acatcaaataaagttttttgatt 18
      ||||||||||||-|--| |||-|||:||-|-|||| |:||||||||||||| ||
10299 aatttaagagatagcatcacatacatagcatacataagataaagttttttgttt 10352
```

Name	From	To	Name	From	To	Dir	Sim	Pos/Mm:Ts	Score
TmSat104	75	122	BEL-30_CloCur-I	5139	5183	c	0.8261	7.0000	74

```
 122 acacgatttttgaccgattttcttccatatttgcaatttttaagccat 75
     ||||||||| |---|| || ||| |||:|||||||||||| | |||||
5139 acacgatttgt---cgtttgtctgccacatttgcaattttgatgccat 5183
```

Name	From	To	Name	From	To	Dir	Sim	Pos/Mm:Ts	Score
TmSat104	194	300	Troyka-1_PtyJor-I	774	901	d	0.7876	4.0000	92

```
194 attaattataaaaatattggtagtcaaaaa----aata-t-------cacgacca---aaataagtt-at 247
    ||| ||||||||| ||||||||-| | |||----|:||-|-------||| || |---||||||:||-||
774 attcattataaaattattggta-taacaaattttagtacttttaccgcactacaaattaaataaattaat 842

248 t-tt-atg----------tcaaaaaatgcattgaatatgaattttactaatttttttagtgataa 300
    |-||-|||----------|:||| |||||||| |||||||||||||--||-| |||||--|||||
843 tattaatggtataataattaaataatgcattcaatatgaattta--aa-tattta--gataa 901
```

TmSat105

Name	From	To	Name	From	To	Dir	Sim	Pos/Mm:Ts	Score
TmSat105	90	123	Copia-7_RC-I	1203	1233	c	0.9091	1.0000	70

```
 123 gaaaatttgctcaaatttcctaaataattttctc 90
     |||||||--|||||||||||:||||||-||||||
1203 gaaaatt--ctcaaatttcccaaataa-tttctc 1233
```

TmSat106

Name	From	To	Name	From	To	Dir	Sim	Pos/Mm:Ts	Score
TmSat106	26	89	Gypsy-14_Cas-LTR	681	740	c	0.8254	1.6667	86

```
 89 aaaatccttatttttagctcaaattaccttgcacgtaaaaagtatgacatttcatc-ttc--attta 26
    |||||||||||||||--||||||-|||:||:||| ||||---|||- |||||:|||-|||--|||||
681 aaaatccttattttt--ctcaaa-tactttacacctaaa---tat-ccattttatctttccaattta 740
```

Name	From	To	Name	From	To	Dir	Sim	Pos/Mm:Ts	Score
TmSat106	94	121	Neptune-13_PMon	6357	6382	d	0.9630	99.0000	68

```
  94 gaaaactagaaaaatagtagattacata 121
     |||||||||||||||--|||||||||||
6357 gaaaactagaaaaat--tagattacata 6382
```

Name	From	To	Name	From	To	Dir	Sim	Pos/Mm:Ts	Score
TmSat106	183	212	Gypsy-42_CAn-I	2404	2433	c	0.9333	2.0000	76

```
 212 gttttcttttcttgaaagaagatgattgtg 183
     ||||||:||||||||| |||||||||||||
2404 gttttcctttcttgaatgaagatgattgtg 2433
```

TmSat107

Name	From	To	Name	From	To	Dir	Sim	Pos/Mm:Ts	Score
TmSat107	52	90	Gypsy-33_AnFu-I	3710	3749	c	0.8718	99.0000	70

```
  90 ataaca---aaagacaataaaccaacagcttcaaaccaaatt 52
     ||||||---|||||||||| |||||||--| || ||||||||
3710 ataacactgaaagacaatataccaaca--tacataccaaatt 3749
```

Name	From	To	Name	From	To	Dir	Sim	Pos/Mm:Ts	Score
TmSat107	158	207	Gypsy-655_AA-I	4612	4654	c	0.8667	99.0000	76

```
 207 ccaacagcttcaaatcttagtaataaaaagaaacaaggttcaaaaattcg 158
     ||||-||||| |||||  ||| |||||||||||||||------|||||||
4612 ccaa-agcttgaaatcgaagttataaaaagaaacaag------aaattcg 4654
```

TmSat108

Name	From	To	Name	From	To	Dir	Sim	Pos/Mm:Ts	Score
TmSat108	309	372	hAT-3N1_CGi	1275	1336	c	0.7656	2.4000	76

```
 372 aaaaacattt-tatagataccgaaaaagcctcaaaaagtctttgttttttgaaaaatcaaatgaa 309
     ||| ||| ||-|||| ::|| ||||| |:-||||||||||:|||||| |||| ||||--:|||||
1275 aaatacagttgtatacgcacggaaaacgt-tcaaaaagtccttgtttattgacaaat--gatgaa 1336
```

TmSat109

Name	From	To	Name	From	To	Dir	Sim	Pos/Mm:Ts	Score
TmSat109	10	70	Gypsy-35_NVi-I	2155	2212	d	0.8000	3.5000	72

```
  10 tatttaccaagttaaaaaaaag--tgcaaaacattccctgtttttgtataaactt-aaaaaaat 70
     || || |||||--||:||:|||--|| ||||-||||---|||||||||| |||||- |||||||
2155 tagttcccaag--aagaagaaggatggaaaa-attc---gtttttgtattaacttccaaaaaat 2212
```

Name	From	To	Name	From	To	Dir	Sim	Pos/Mm:Ts	Score
TmSat109	204	240	Gypsy-53_SpEx-LTR	26	66	c	0.8718	99.0000	72

```
240 att-ttttacaa---gtcggtgaaaacaattctatttttaa 204
    |||-||||||||---|||||| ||| ||| |||||||||||
 26 attgttttacaatttgtcggttaaatcaagtctatttttaa 66
```

TmSat110

Name	From	To	Name	From	To	Dir	Sim	Pos/Mm:Ts	Score
TmSat110	22	86	Gypsy-50_CQ-I	8125	8185	c	0.8226	6.0000	80

```
  86 agtaatttaagtaatg--accagaaattgaaaaatactgcttgattaaatt--cttattcaaaaaaaaa 22
     ||||  ||||||||||--|:|--||||||||||| |-----|||-||||||--||||  ||||||||||
8125 agtatattaagtaatggtatc--aaattgaaaaaaa-----tga-taaattcacttaaacaaaaaaaaa 8185
```

TmSat111

Name	From	To	Name	From	To	Dir	Sim	Pos/Mm:Ts	Score
TmSat111	47	112	Gypsy-339K_ZM-LTR	683	751	d	0.7500	7.0000	72

```
 47 aaacaacatgttttcaaacatgtt---ttttgtatca-aaaatttacagaaaatgcccaaaaatctgtga 112
    ||| | |||| |:| |||||| | ---||||:|-|||-|||||  ||||||||||   ||||||||| ||
683 aaaaatcatgatctaaaacatctgcaattttat-tcataaaataaacagaaaatggggaaaaatctgaga 751
```

Name	From	To	Name	From	To	Dir	Sim	Pos/Mm:Ts	Score
TmSat111	144	299	Tx1-3_SPur	3657	3805	c	0.7237	3.3750	86

```
 299 aaaatgttatttgtaaaaacaaaaaaaa------atatttgctataatttgga---caagaaattcgtcg 239
     |||||||||||||-||| |||||:||||------|||:||-| |||||  |||---:|||||| |  | |
3657 aaaatgttatttg-aaatacaaagaaaatataagatactt-cgataatgaggaagttaagaaagtattag 3724

 238 aaattagtcatacaat-ttc-tcgtttcataaattaaaaagtgctttttggtgt-tacaattggcatgac 172
     ||||--||--|| |||- ||-|--||| | | ||||-----||-||  |  |||-||:||||--||||--
3725 aaat--gt--taaaatgatcat--tttaaganatta-----tg-ttgatcttgtatataatt--catg-- 3778

 171 ttttattatatttcatttttcaaataat 144
     |||:||| ||:||-:|||| :|||||||
3779 tttcattttactt-gttttgtaaataat 3805
```

TmSat115

Name	From	To	Name	From	To	Dir	Sim	Pos/Mm:Ts	Score
TmSat115	4	99	Daphne-30_LMi	3174	3260	d	0.7802	8.0000	70

```
   4 actgctttgaacaaaa-tct--gaatatcactttgatttccga-aaaacttggaaaacaccttcttttat 69
     ||||||||||| ||||-|||--|-:||-||---|||||----|-|||||| |||----||| |-| | ||
3174 actgctttgaaaaaaattcttcg-gta-ca---tgatt----ataaaactagga----accgt-tgtaat 3229

  70 cttcaaactctt---agaaaaaa-aaattgaaaa 99
     || ||---||||---||||||||-||| ||||||
3230 ctgca---tcttgaaagaaaaaataaagtgaaaa 3260
```

TmSat116

Name	From	To	Name	From	To	Dir	Sim	Pos/Mm:Ts	Score
TmSat116	14	188	Polinton-1C_HM	9553	9752	d	0.7322	5.6667	112

```
  14 tttataaaaa---acaaacaaaataagaaataataattataaa-aat--agaaaaaaaatattttttttt 77
     || |||||||---|  ||| ||| ||| |||||| | ||||||-|||--||| ||  |||:||||    |
9553 ttgataaaaatttagcaactaaaaaagcaataattaatataaagaatgaagataatcaatgttttaagtg 9622

  78 gtaaaatattgcaag---attaaa---aa-a---aataggtctgaaa----agctaaa-aacttaaaaac 132
     ||:---|||||||||---||||||---||-|---||||: :|| |||----|| ||||-|:||||||||-
9623 gtg---tattgcaagagcattaaatccaacagataataatcctcaaagagtagataaagagcttaaaaa- 9688

 133 ttcaag----aattctaaaatcggataaaaaagcgaaaatcca--at-----aatcgaagaacacaa 188
     -|||||----||   |||| | |||||||| |--|||  ||||--||-----||||:||  ||||||
9689 -tcaagctgaaaaaataaactgggataaaata--gaatttccagtatccttaaatcaaatcacacaa 9752
```

TmSat117

Name	From	To	Name	From	To	Dir	Sim	Pos/Mm:Ts	Score
TmSat117	26	77	DIRS-4_SpEx	5112	5157	d	0.8542	99.0000	76

```
  26 cataaagtctcg-tgttaattaggaattaatgccttgttttgagctgaaaaaa 77
     |||||||----|-|||||||||---|| ||||||||||||| | || ||||||
5112 cataaag----gatgttaatta---ataaatgccttgttttcaccttaaaaaa 5157
```

TmSat118

Name	From	To	Name	From	To	Dir	Sim	Pos/Mm:Ts	Score
TmSat118	269	324	Gypsy-N1_OD-I	1748	1801	d	0.7857	9.0000	72

```
 269 aaaaaaaattctttcaaagatttaaaacgtgtgaataaaattcacaact-tcaaaat 324
     |||| ||||||  --||| |||| :|||  |||||||||||||-|||||- ||||||
1748 aaaataaattcga--aaatattttgaactagtgaataaaattc-caactggcaaaat 1801
```

TmSat120

Name	From	To	Name	From	To	Dir	Sim	Pos/Mm:Ts	Score
TmSat120	81	124	KolobokE-1_OcyOle	4755	4794	d	0.8571	99.0000	74

```
  81 agaaaaattgcataattagcgtcaaattgacgtaaatttgtttc 124
     ||||||||||||||||||---||| |||  |- |||||||||||
4755 agaaaaattgcataatta---tcatattttc-aaaatttgtttc 4794
```

TmSat121

Name	From	To	Name	From	To	Dir	Sim	Pos/Mm:Ts	Score
TmSat121	4	31	Mariner3-2N1_LMi	805	833	c	0.9310	99.0000	70

```
 31 agtgtaacggaatttaattttcaa-acct 4
    ||||||||| ||||||||||||||-||||
805 agtgtaacgtaatttaattttcaatacct 833
```

Name	From	To	Name	From	To	Dir	Sim	Pos/Mm:Ts	Score
TmSat121	35	84	EnSpm-N1_DTa	868	923	d	0.8113	3.5000	74

```
 35 tataaaaatatttcttaaaaat-gaaatgtttt-tgaa----gagcggtttttcaa 84
    || |||||||||||| ||||||-|||||:||||-||||----:||  | |||||||
868 tagaaaaatatttctgaaaaatagaaatattttctgaatttcaaggcgattttcaa 923
```

Name	From	To	Name	From	To	Dir	Sim	Pos/Mm:Ts	Score
TmSat121	119	217	hAT-79_SM	1466	1571	c	0.7549	7.5000	78

```
 217 ttactcttttcttaatgaattttattggaaaaactgtattg--aatat------t--aaaaaaaatatac 158
     ||| || |||--|||--|||||| :| ||||||||||||||-- ||||------|--|| |||||| |||
1466 ttaatcattt--taa--aattttcttgaaaaactgtattgcctatatagccaatccaataaaaatctac 1531

 157 agaagc-taa--aaaaagcaaagtagatactt-gtttcgtaat 119
      |:|||-|||--| |  |||||--||||-|||-|||| | |||
1532 tggagcttaacgatattgcaaa--agat-cttggtttagaaat 1571
```

TmSat122

Name	From	To	Name	From	To	Dir	Sim	Pos/Mm:Ts	Score
TmSat122	55	128	Academ-59_ArMa	978	1049	c	0.8082	4.0000	90

```
128 ttttacgtcaaatcgatcttaaaaacaaaggtcacagctgaatgttttgacaa---attaaaga-aggaa 63
    ||||||||----||-||: |||||||||||| | ||||||||:| |||--|||---||||||||-|  ||
978 ttttacgt----tc-attgtaaaaacaaaggacccagctgaacgattt--caacggattaaagacattaa 1040

  62 aaaa-tgag 55
     ||||-||||
1041 aaaactgag 1049
```

TmSat123

Name	From	To	Name	From	To	Dir	Sim	Pos/Mm:Ts	Score
TmSat123	359	454	Gypsy-15_HRo-I	1486	1592	c	0.7812	1.8333	68

```
 454 tgagaaattaaaaaaacgtcatttgataaag--ttcaagatttcattaaagaagtgg--ttaaaaat--- 392
     |||:||||||:|||----|||---||:||| --||||||||||||  |||||--|||-- |||||||---
1486 tgaaaaattagaaa----tca---gacaaacaattcaagatttcaagaaaga--tggaagtaaaaataaa 1546

 391 gtgagatttcaa-------ataaaa-----at--ttcaacagcttag 359
     :|-|:|||||||-------|||||-----||--||||||| ||:||
1547 at-aaatttcaatgaacatataaaaacgacatgcttcaacatctcag 1592
```

Fig. S2. Alignments of partial sequence segments between *T. madens* satDNAs and Repbase-deposited repetitive elements. Similarities between satDNAs and Repbase entries were accessed by the CENSOR tool on October 17th 2025.

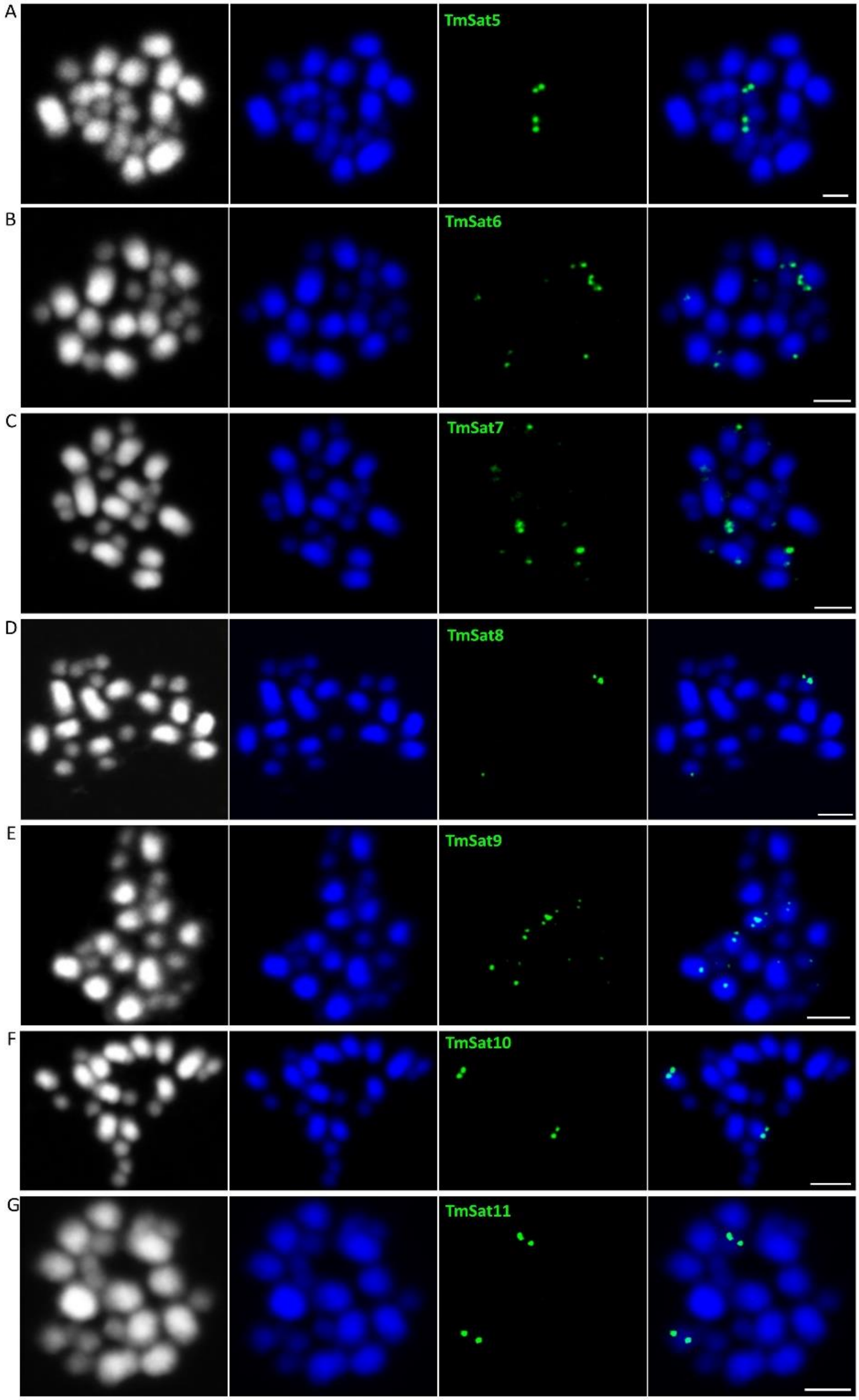

Fig. S3. Localization of the low-copy-number satDNAs TmSat5 **(A)**, TmSat6 **(B)**, TmSat7 **(C)**, TmSat8 **(D)**, TmSat9 **(E)**, TmSat10 **(F)**, and TmSat11 **(G)** on the *T. madens* metaphase chromosomes (2n=20+supernumeraries) determined by fluorescence *in situ* hybridization. The first panels show the chromosomes in a black and white version to better visualize the contours of the chromosomes. The chromosomes are stained in DAPI (blue fluorescence), while satDNA-specific probes are shown in green. The bar represents 3 µm.

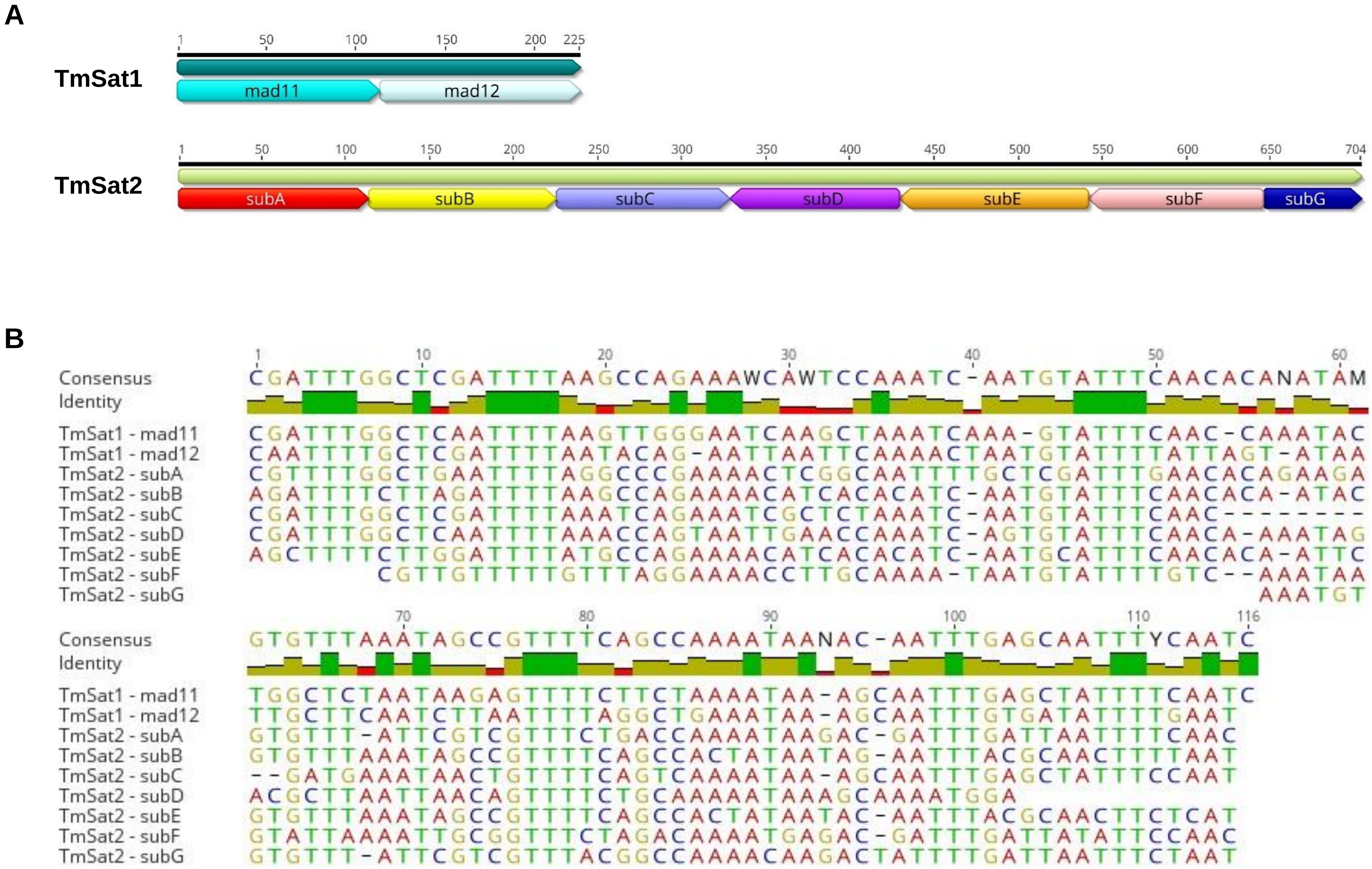

	mad1_1	mad1_2	subA	subB	subC	subD	subE	subF	subG
mad1_1		67.54%	57.39%	65.22%	74.34%	71.84%	59.13%	50.47%	56.67%
mad1_2	67.54%		53.91%	61.40%	63.72%	63.11%	55.26%	51.85%	56.67%
subA	57.39%	53.91%		59.65%	54.78%	57.28%	59.65%	59.81%	77.97%
subB	65.22%	61.40%	59.65%		66.37%	63.73%	92.86%	57.94%	63.33%
subC	74.34%	63.72%	54.78%	66.37%		70.30%	62.83%	54.72%	53.33%
subD	71.84%	63.11%	57.28%	63.73%	70.30%		60.78%	49.47%	58.33%
subE	59.13%	55.26%	59.65%	92.86%	62.83%	60.78%		57.94%	65.00%
subF	50.47%	51.85%	59.81%	57.94%	54.72%	49.47%	57.94%		63.33%
subG	56.67%	56.67%	77.97%	63.33%	53.33%	58.33%	65.00%	63.33%	

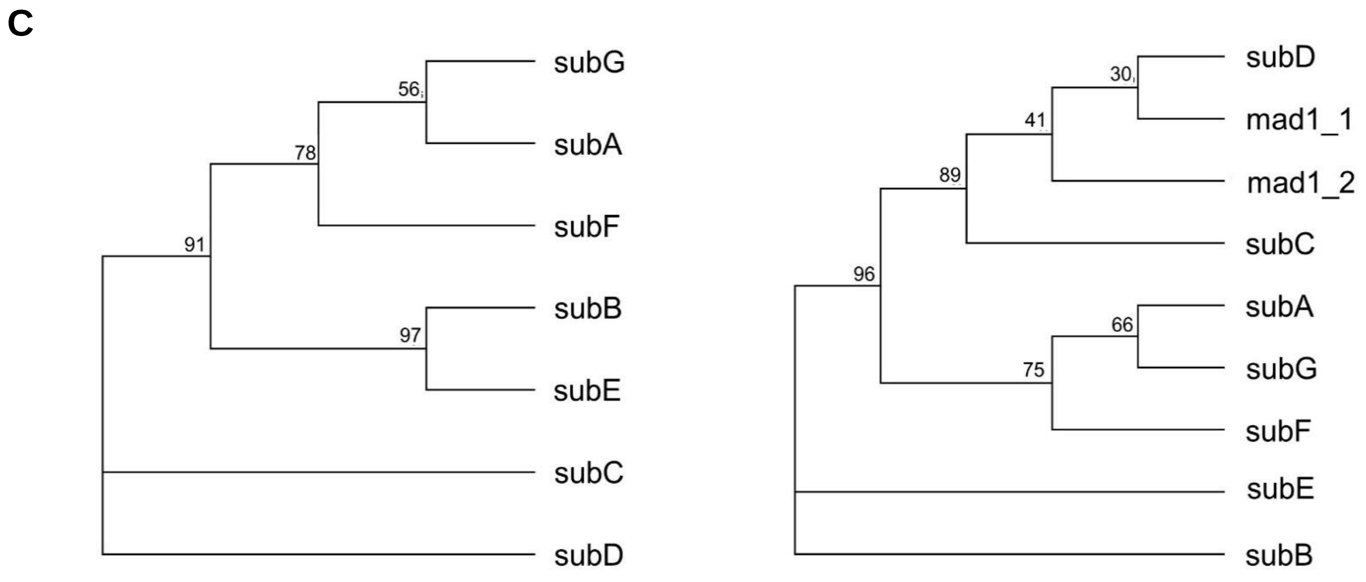

Fig. S4. Structural organization of TmSat1 and TmSat2 repeat units. **(A)** Schematic representation of TmSat1 and TmSat2 structure and the organization of their subunits. The arrows indicate the orientation of the subunits. **(B)** Alignment of the TmSat1 and TmSat2 subunits and matrix of their pairwise similarities. **(C)** Maximum likelihood trees reflecting relationships between the TmSat2 subunits (left) and the TmSat1-TmSat2 subunits (right). The numbers above the branches indicate the percentage of bootstrap values for 100 replicates.

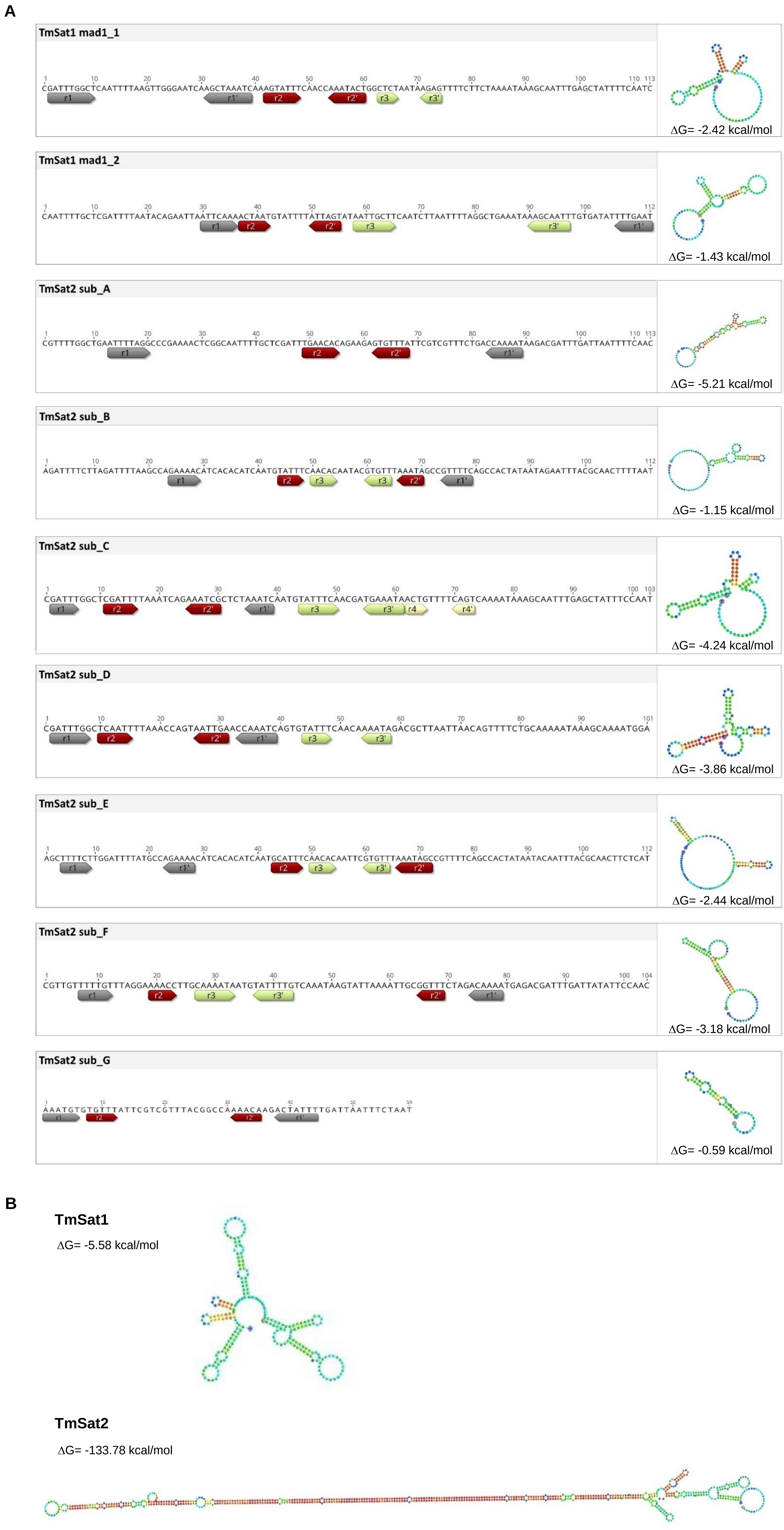

Fig. S5. Short (<10 bp) inverted repeats in the subunits of TmSat1 and TmSat2 **(A)**, and predicted secondary structures of TmSat1 and TmSat2 repeat units **(B)**. Potential secondary structures were predicted using the RNAfold tool [63] and DNA Matthews 2004 energy model.

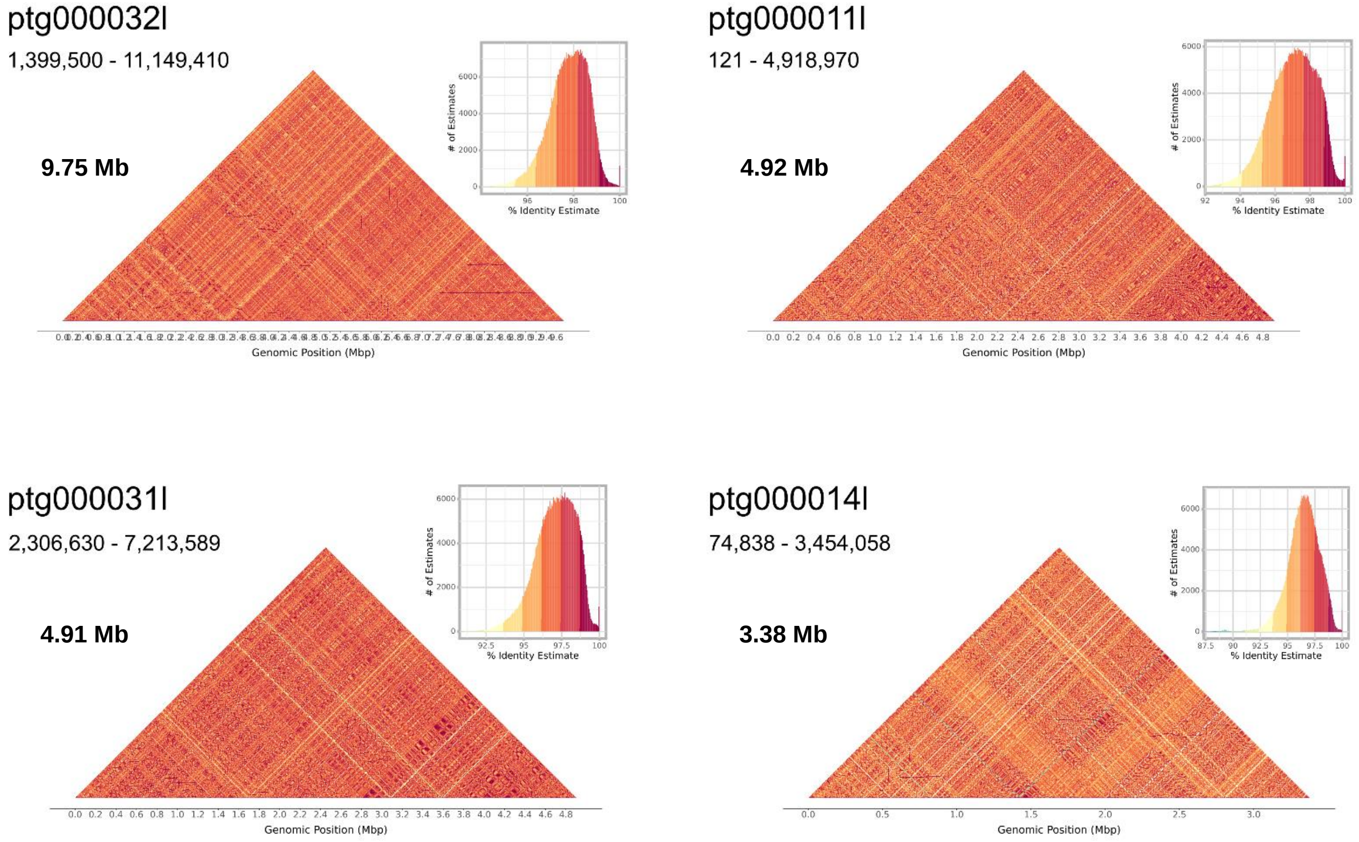

Fig. S6. The ModDotPlot visualization of the multi-megabase sized regions containing intermingled arrays of TmSat1 and TmSat2 satDNAs in the four contigs (ptg000032l, ptg000011l, ptg000031l, and ptg000014l) from the Tmad1.0 assembly. The lengths and positions of the TmSat1-TmSat2 regions within the contigs are indicated below the contig names, and the identity estimate values are shown in the color histogram along each plot.

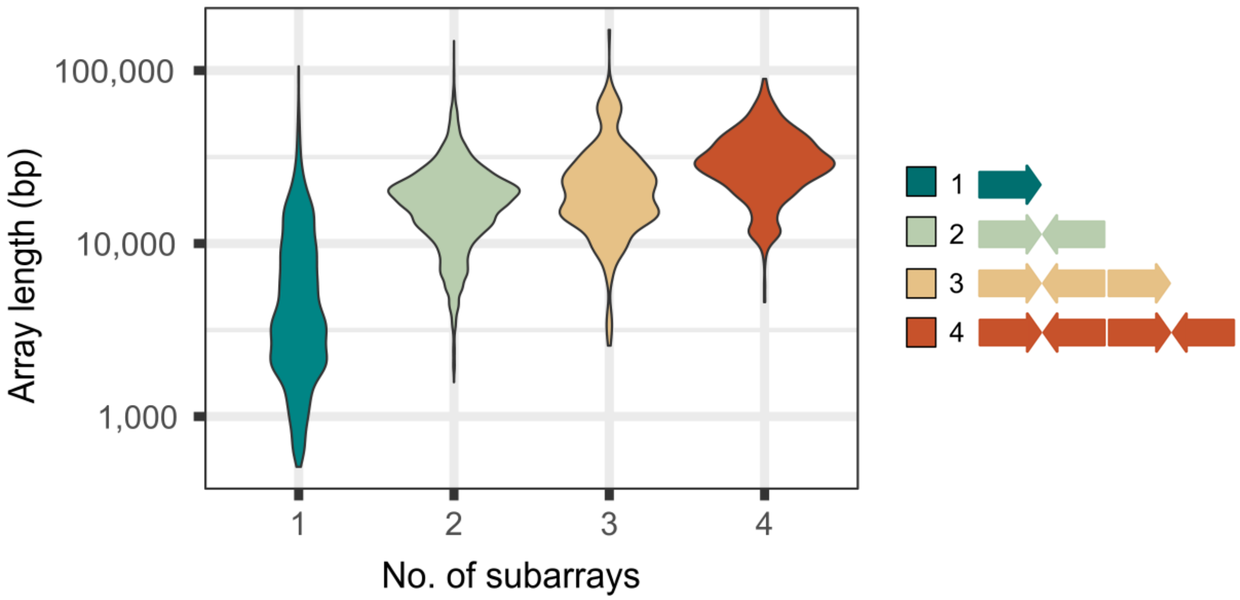

Fig. S7. The length of the TmSat1 arrays related to the number of subarrays they contain.

A

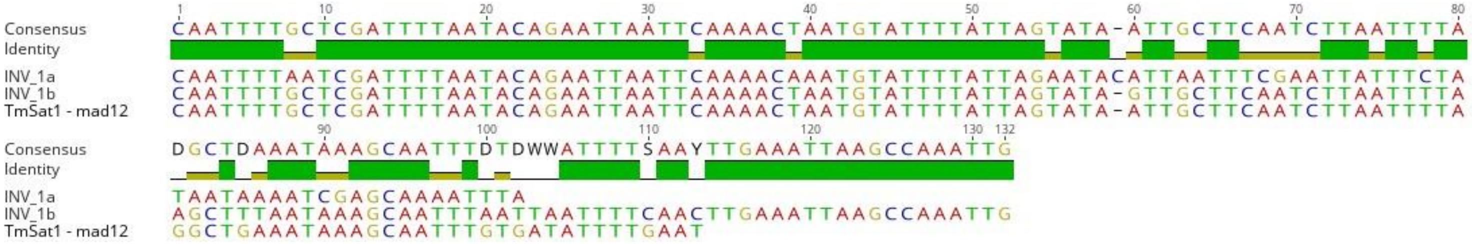

	INV_1a	INV_1b
INV_1a		72.55%
INV_1b	72.55%	
TmSat1 - mad12	76.47%	89.29%

B

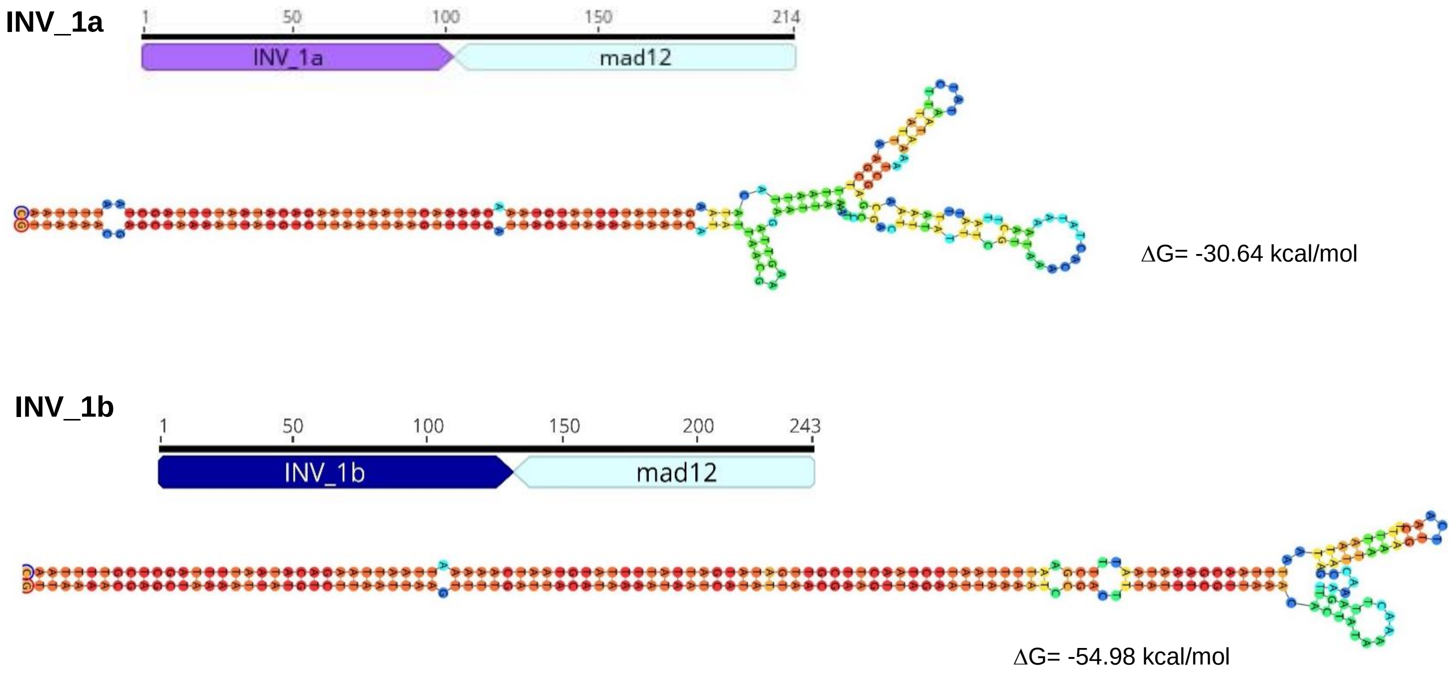

Fig. S8. Diverged subunits INV_1a and INV_1b from the inversion sites in the TmSat1 arrays. **(A)** Alignment of INV_1a, INV_1b and the canonical TmSat1 subunit mad1_2, and the matrix of their pairwise similarities. **(B)** Predicted secondary structures formed at the INV_1a and INV_1b inversion sites. The potential hairpin conformations were predicted using the RNAfold tool [63] and DNA Matthews 2004 energy model.

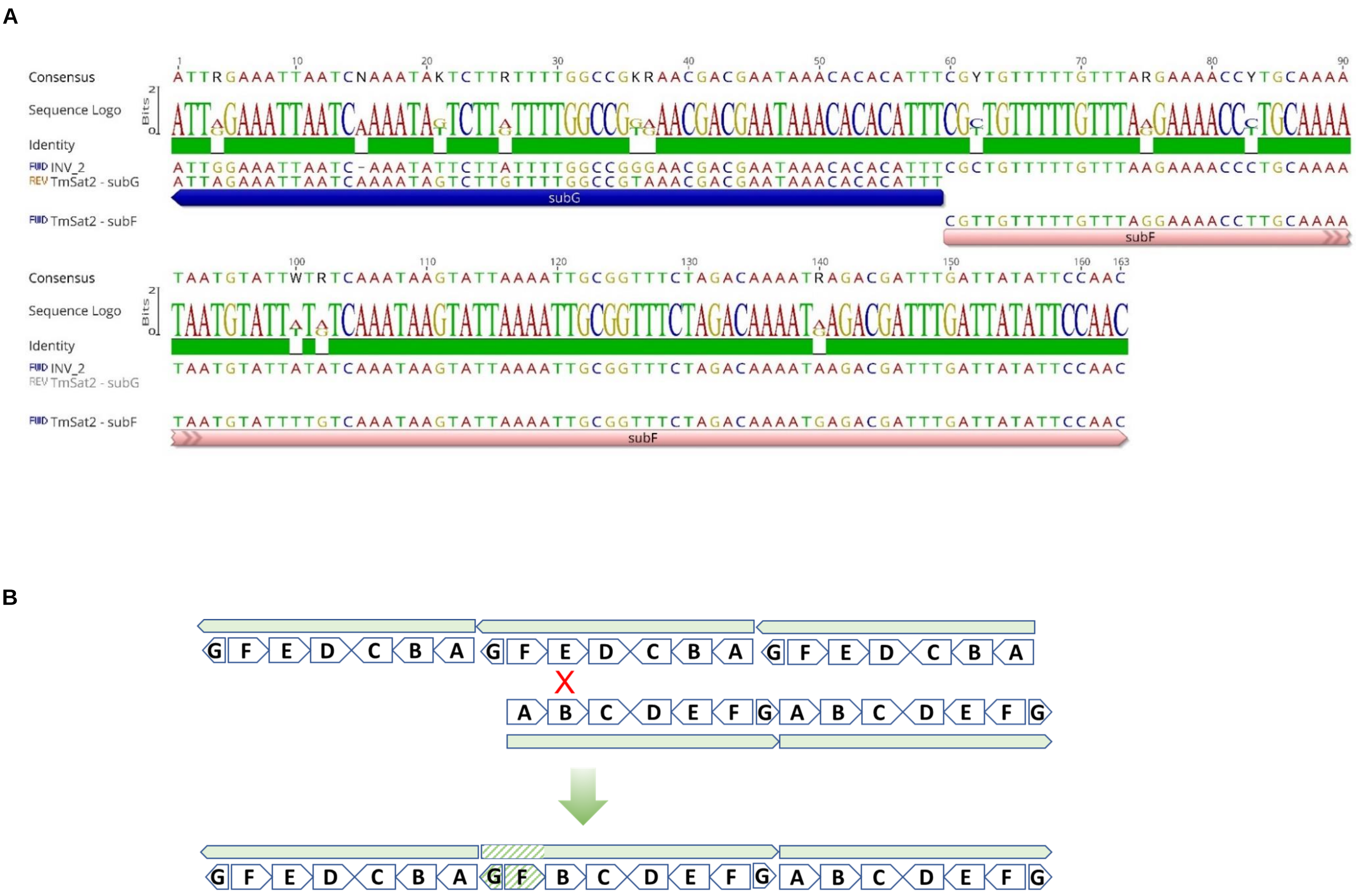

Fig. S9. Structure of the inversion site INV_2 in the TmSat2 arrays and a possible explanation for its formation. **(A)** Alignment of the INV_2 sequence and the canonical TmSat2 subunits sub_G and sub_F. The sequence logo indicates 12 nucleotide differences between INV_2 and the subG+subF subunits. **(B)** The most parsimonious scenario of how an inversion rearrangement could occur between TmSat2 tandem repeats and generate the INV_2 site. We propose that ectopic recombination has occurred between two oppositely oriented TmSat2 arrays, most likely between the highly similar subunits sub_E and sub_B, which share the highest pairwise similarity (92.9%) among the TmSat2 subunits (as indicated in Fig. S4B-C).

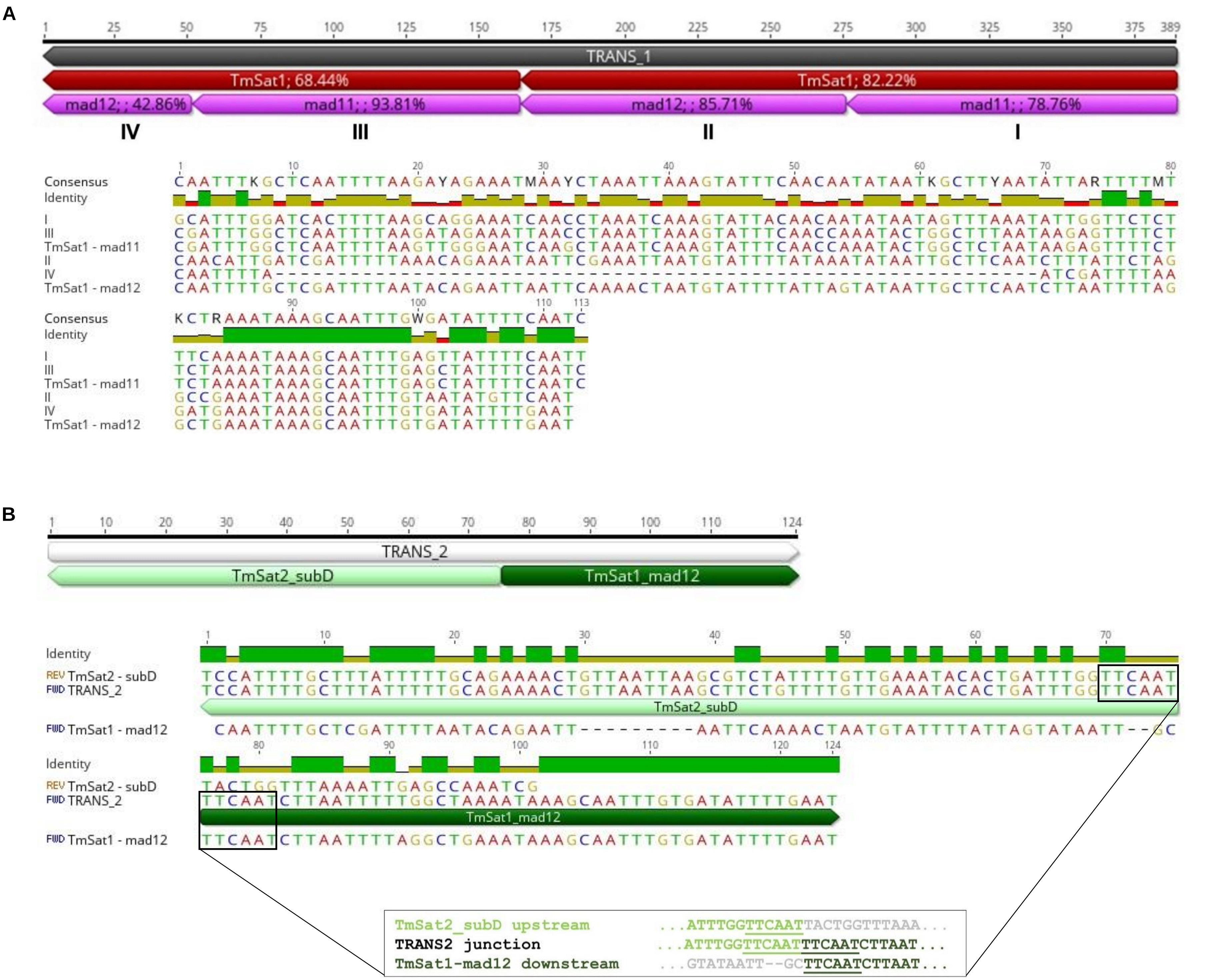

Fig. S10. Structure of the transition zones TRANS_1 and TRANS_2 between the TmSat1 and TmSat2 arrays. **(A)** Schematic representation of the TRANS_1 segment and the alignment of the four subunits (I-IV) and the TmSat1 subunits mad1_1 and mad1_2. The percentages in the arrows indicate the similarity to the corresponding sequences (TmSat1 monomer, subunit mad1_1 and subunit mad1_2). **(B)** Schematic representation of the TRANS_2 segment and the alignment of TRANS_2, TmSat2 subunit sub_D and TmSat1 subunit mad1_2. The box highlights the sharp transition between the subunits sub_D and mad1_2 with the underlined duplication of the TTCAAT motif.

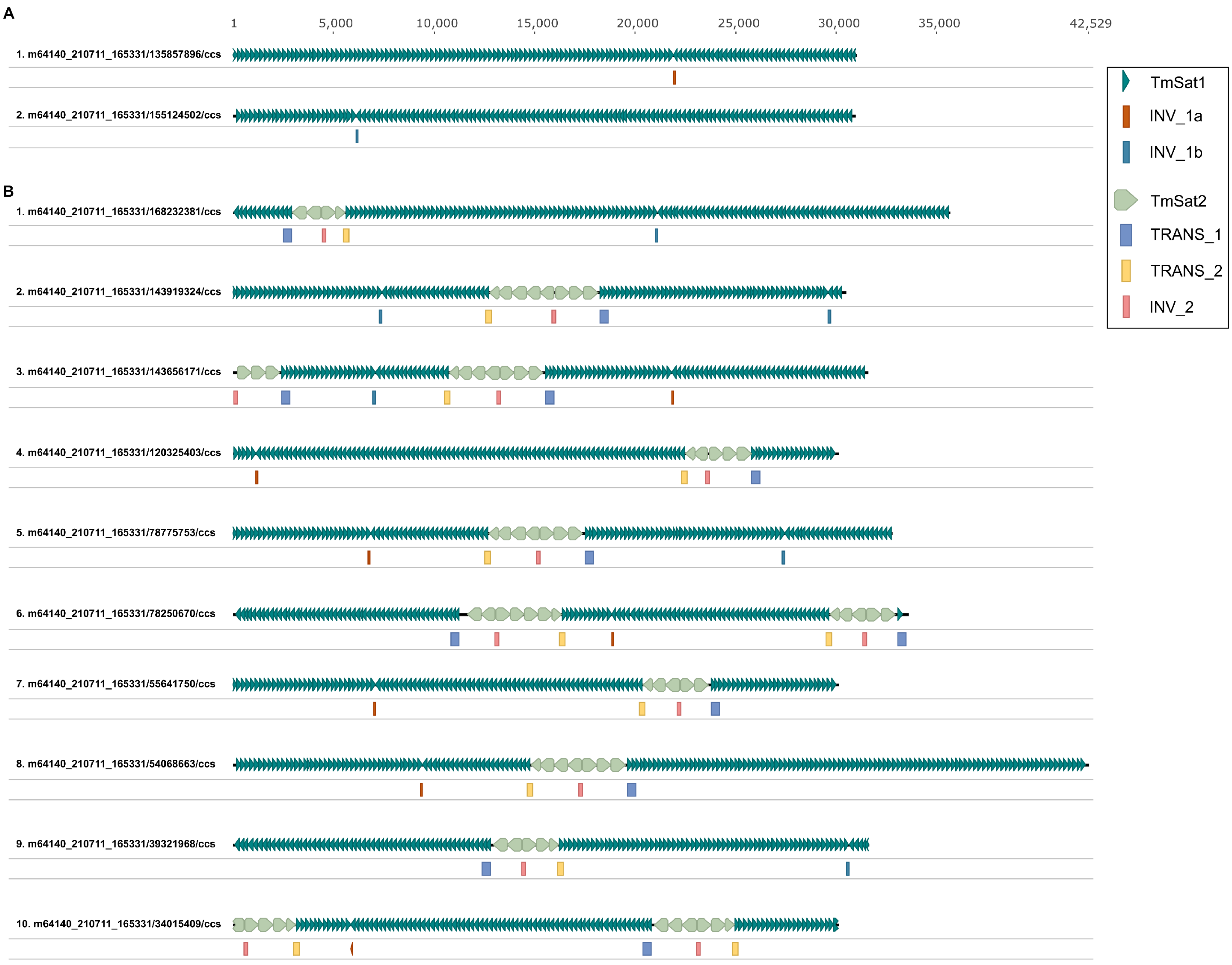

Fig. S11. Visualization of the raw PacBio HiFi sequencing reads (>30 kb) demonstrating the authenticity of macro-dyad symmetries and conserved segments in TmSat1 and TmSat2 arrays. **(A)** The raw reads consisting entirely of TmSat1 repeats, with the conserved INV_1a and INV_1b inversion sites indicated. **(B)** The raw reads showing the intermingled TmSat1 and TmSat2 arrays forming macro-dyad symmetries. The conserved inversion sites INV_1a, INV_1b and INV_2, as well as transition zones TRANS_1 and TRANS_2 are indicated.

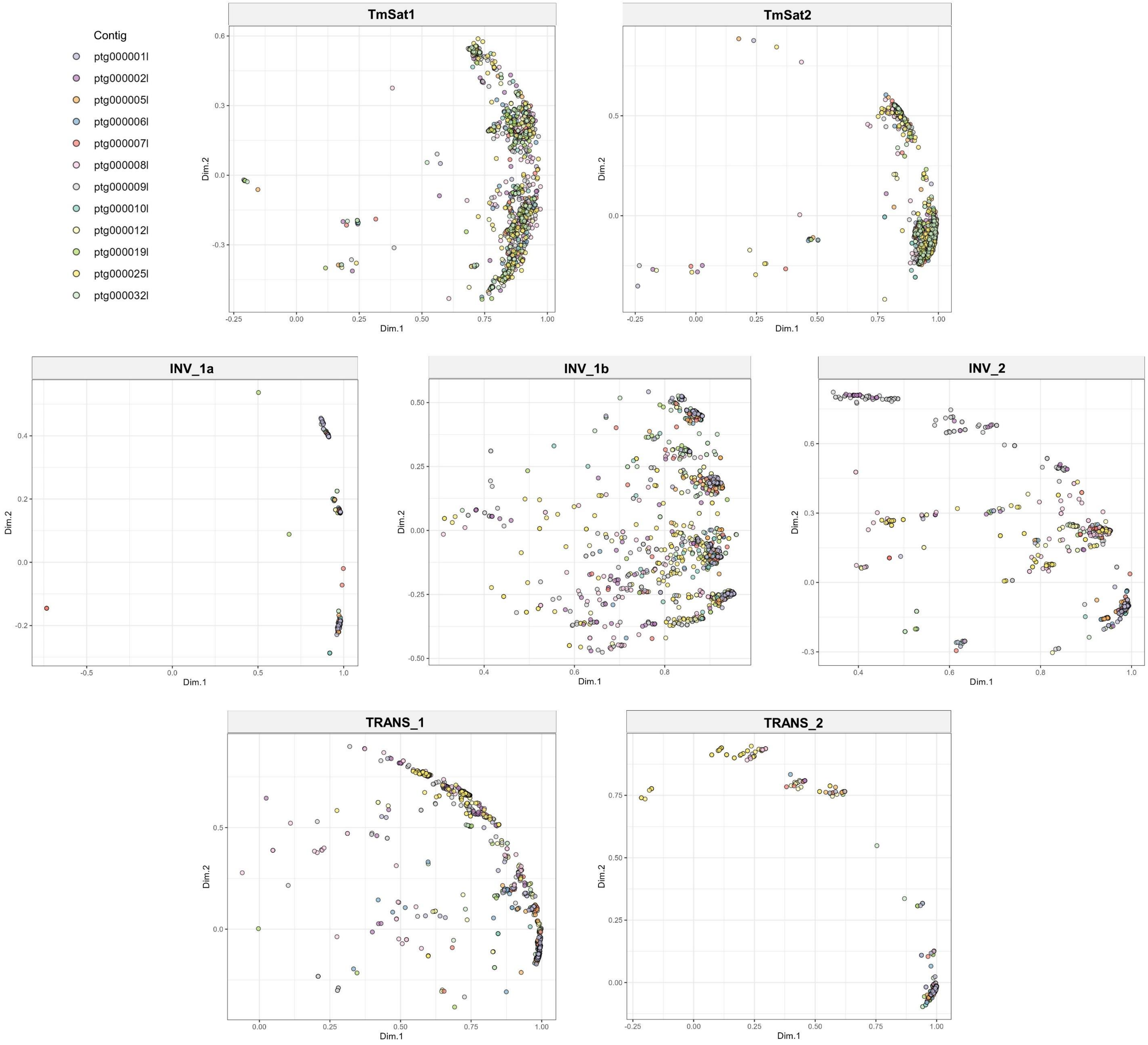

Fig. S12. PCA clustering of TmSat1 and TmSat2 monomers, as well as INV_1a, INV_1b, INV_2, TRANS1, and TRANS2 segments derived from the 12 longest contigs of the Tmad1.0 assembly. Owing to the large number of annotated TmSat1 and TmSat2 monomers, these datasets were downsampled by randomly selecting 100 monomers from each contig. For all other sequence sets, the complete sets of annotated sequences were included. Each dot corresponds to an individual sequence, colored according to its contig of origin.

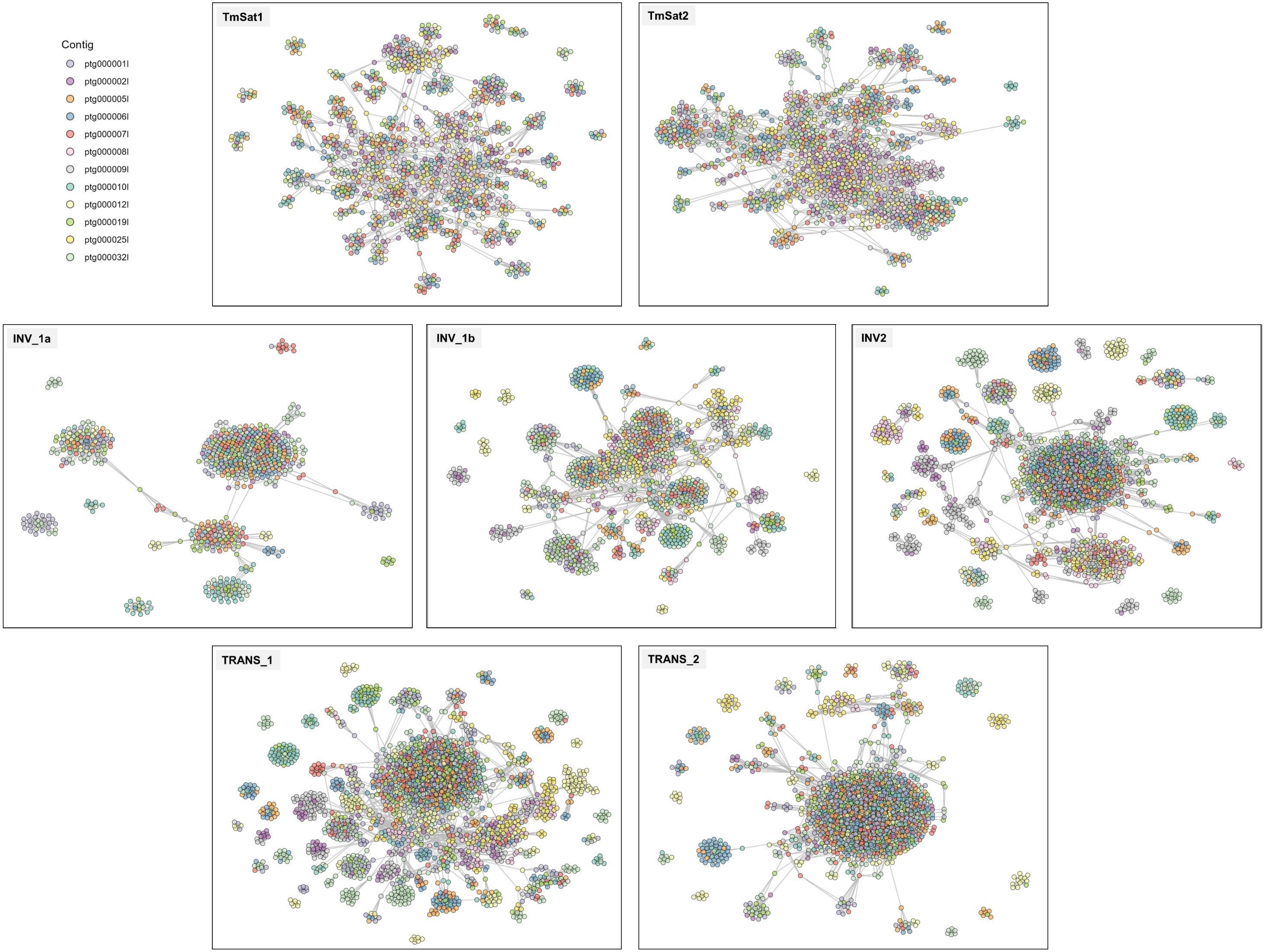

Fig. S13. Graph networks illustrating sequence-similarity relationships among TmSat1, TmSat2, INV_1a, INV_1b, INV_2, TRANS_1, and TRANS_2 sequences extracted from the 12 longest contigs of the Tmad1.0 assembly. These networks were generated from the same sequences analyzed by PCA in Suppl. Fig. S12. Each dot represents an individual sequence, colored according to the Tmad1.0 contig on which it is annotated. A color-coded contig legend is provided.

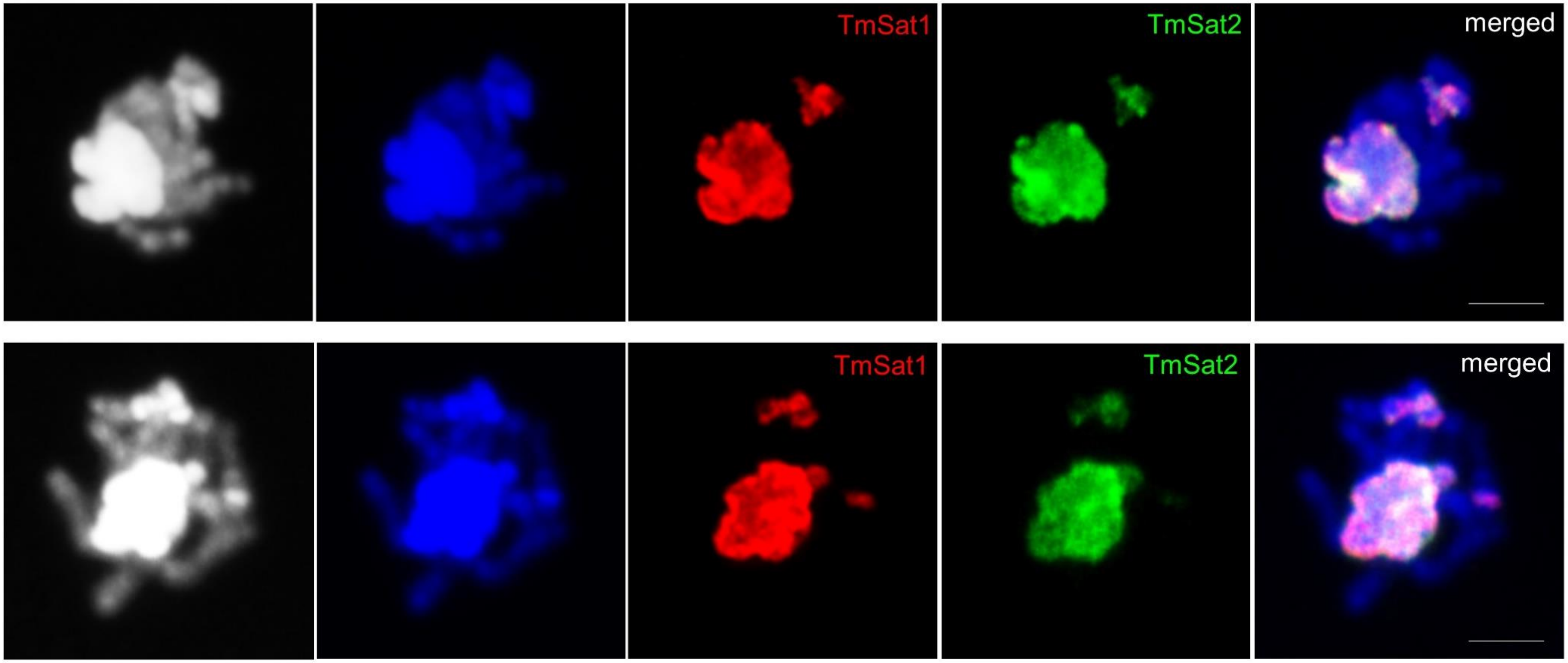

Fig. S14. Meiotic bouquet-like configurations showing associations among non-homologous chromosomes of *T. madens*. The first panels display chromosomes in black and white to better visualize their contours. DAPI-stained chromosomes (blue) were analyzed by two-color FISH using TmSat1 (red) and TmSat2 (green) probes. The final panels show the overlap of the signals. Scale bar = 3 µm.

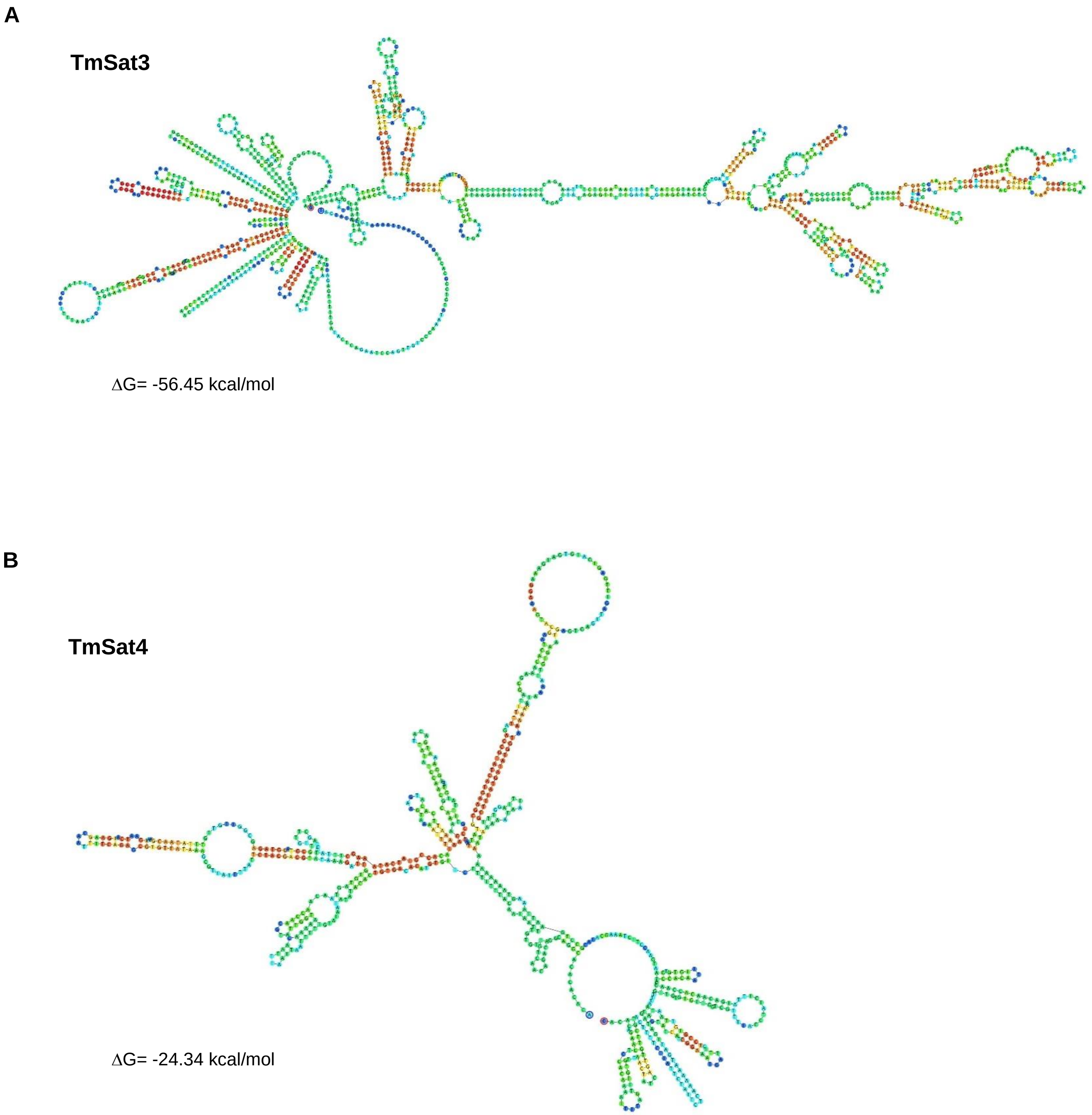

Fig. S15. Predicted secondary structures of TmSat3 **(A)** and TmSat4 **(B)** repeat units. Potential DNA secondary structures were predicted using the RNAfold tool [63] and DNA Matthews 2004 energy model.

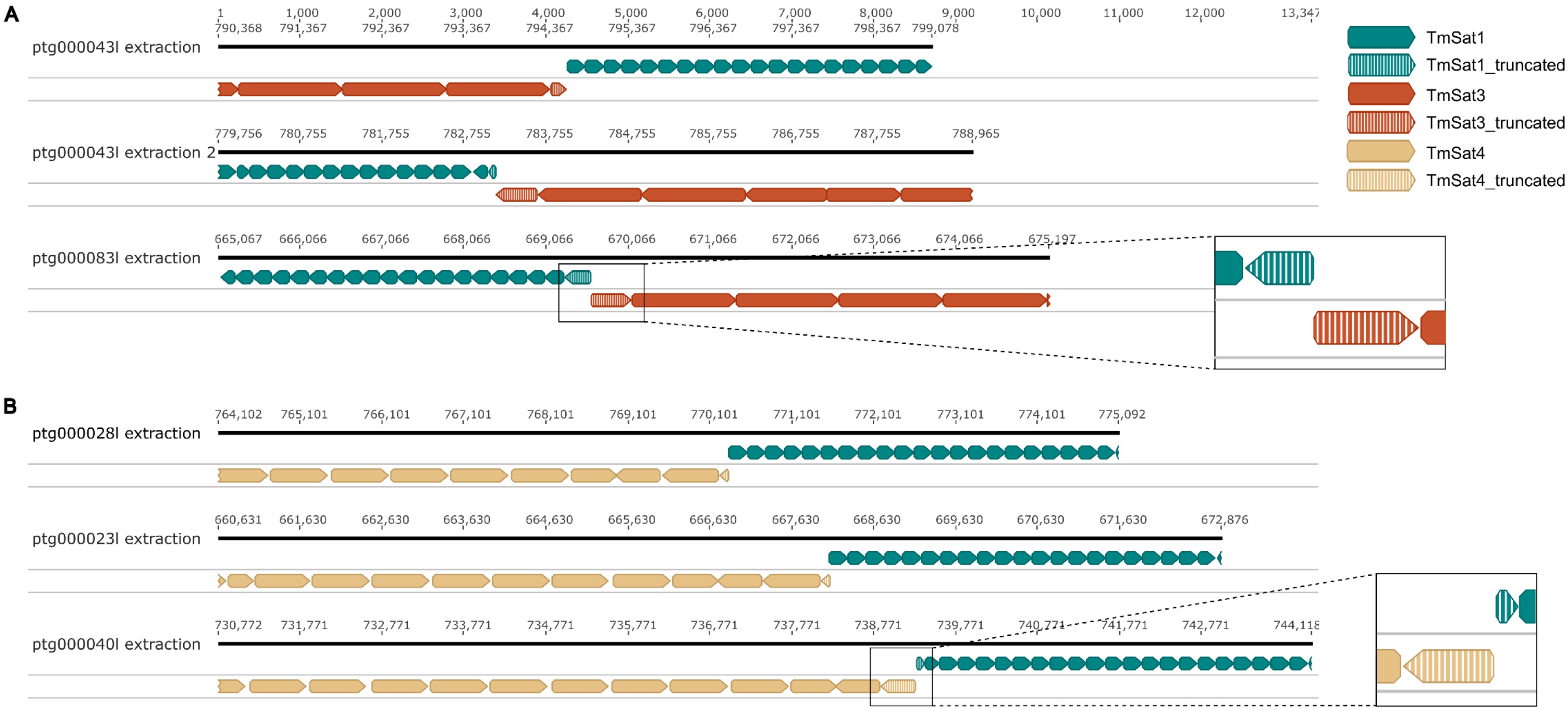

Fig. S16. The abrupt transitions between **(A)** TmSat1 and TmSat3 arrays, and **(B)** TmSat1 and TmSat4 arrays. The transitions represent the junctions of truncated repeats (marked by vertical stripes) from the juxtaposed satDNAs.

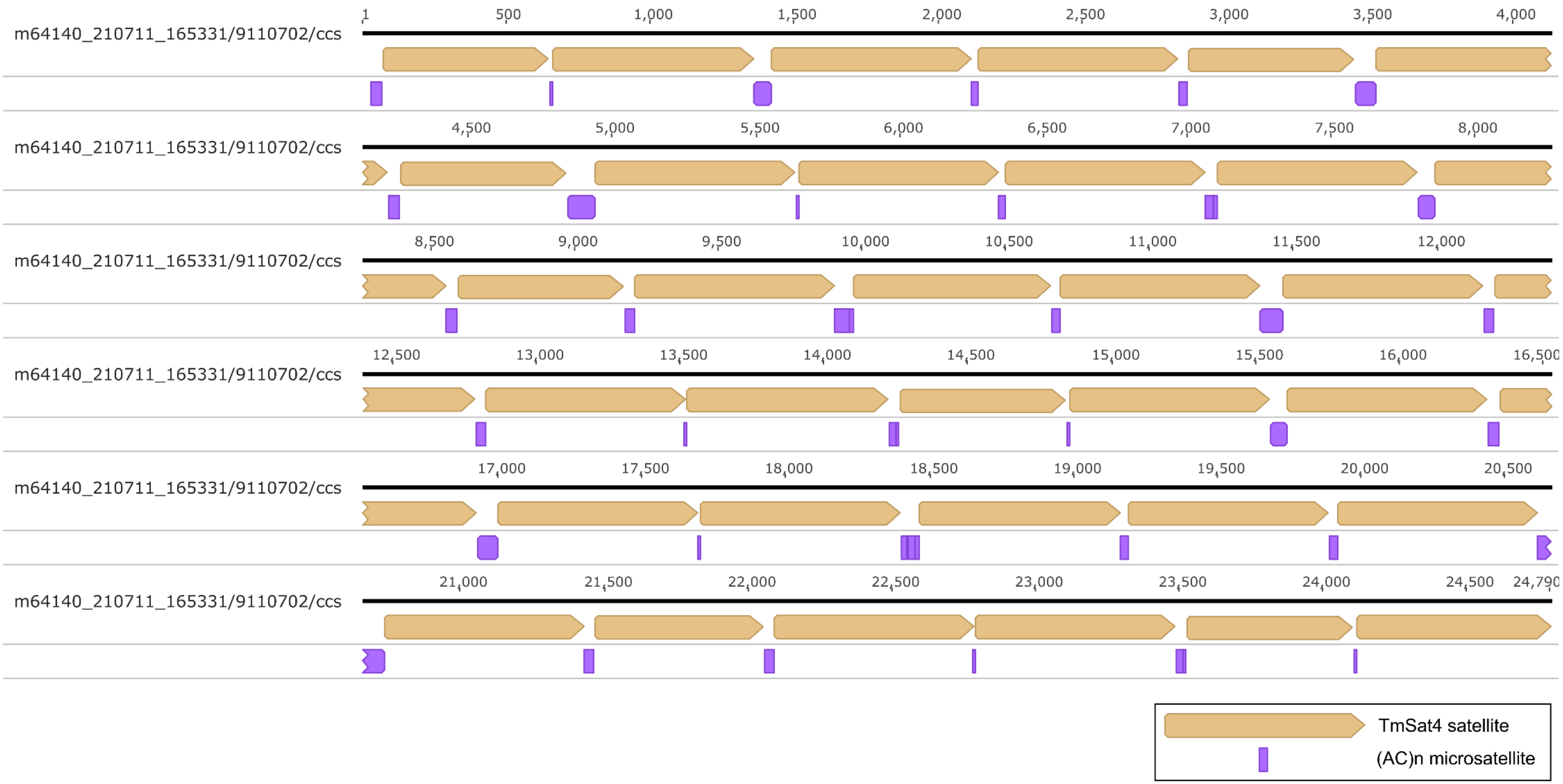

Fig. S17. A raw PacBio HiFi read consisting entirely of TmSat4 repeats separated by microsatellite (AC)n arrays of different lengths.

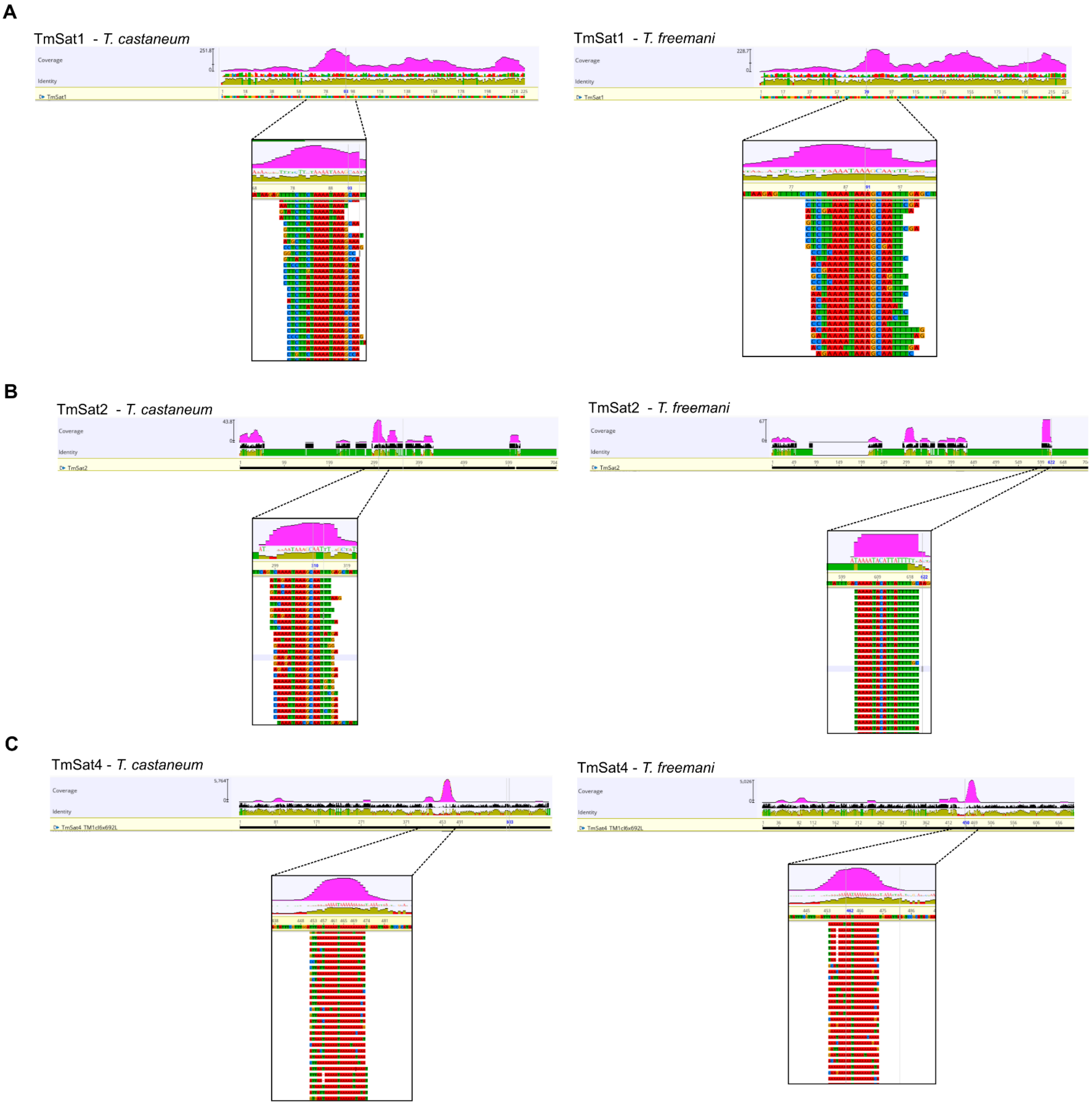

Fig. S18. K-mer analysis of the satDNAs TmSat1, TmSat2 and TmSat4, performed to investigate the possible presence of the orthologous satDNAs in the congeneric species *T. castaneum* and *T. freemani*. Representative k-mers derived from each satDNA were used as queries in a search against the genome assemblies of *T. castaneum* (TcasONT) and *T. freemani* (Tfree1.0). The resulting matching sequences, representing hits from each genome, were then aligned to the corresponding satDNA consensus using the Bowtie algorithm implemented in the Geneious software. This approach revealed that the primary mappings of the TmSat-derived k-mers are mainly to the A+T-rich regions of TmSat1 **(A)**, TmSat2 **(B)** or TmSat4 **(C)** consensus sequences.

A

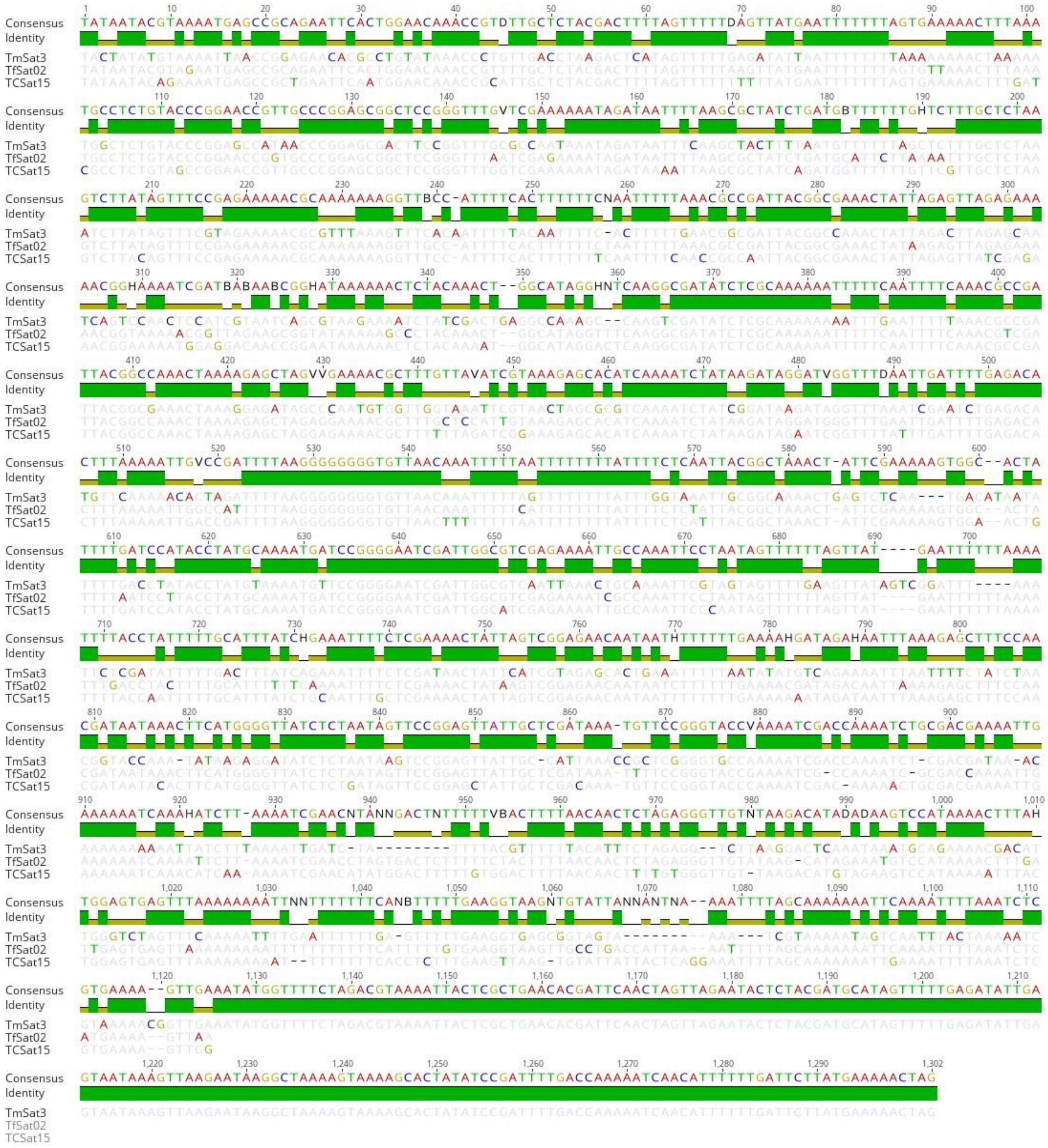

	TmSat3	TfSat02	TCSat15
TmSat3		67.50%	66.49%
TfSat02	67.50%		85.69%
TCSat15	66.49%	85.69%	

B

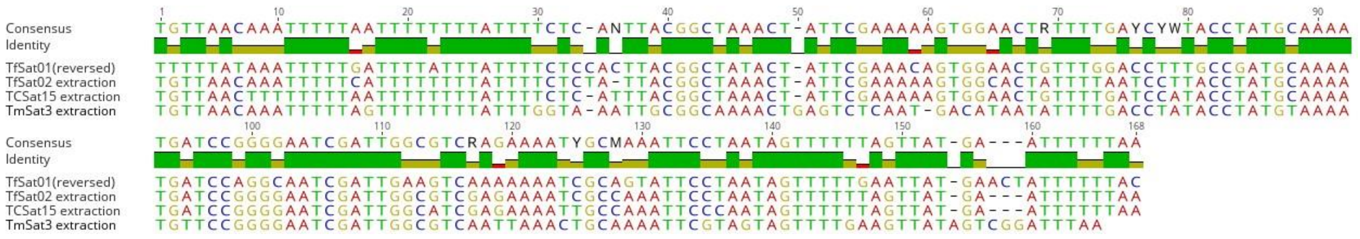

	TfSat01(rev...	TfSat02 ext...	TCSat15 ex...	TmSat3 ext...
TfSat01(reversed)		80.72%	78.31%	67.27%
TfSat02 extraction	80.72%		92.02%	73.94%
TCSat15 extraction	78.31%	92.02%		73.17%
TmSat3 extraction	67.27%	73.94%	73.17%	

Fig. S19. The relationships between the orthologous satDNAs TmSat3 from *T. madens*, TfSat02 from *T. freemani*, and TCsat15 from *T. castaneum*. **(A)** The alignment between TmSat3, TfSat02 and TCsat15, and the corresponding pairwise similarity matrix. **(B)** The alignment between the *T. freemani* major satDNA TfSat01 and the corresponding TfSat01-like segments extracted from TmSat3, TfSat02 and TCsat15. The pairwise similarity matrix is also shown.

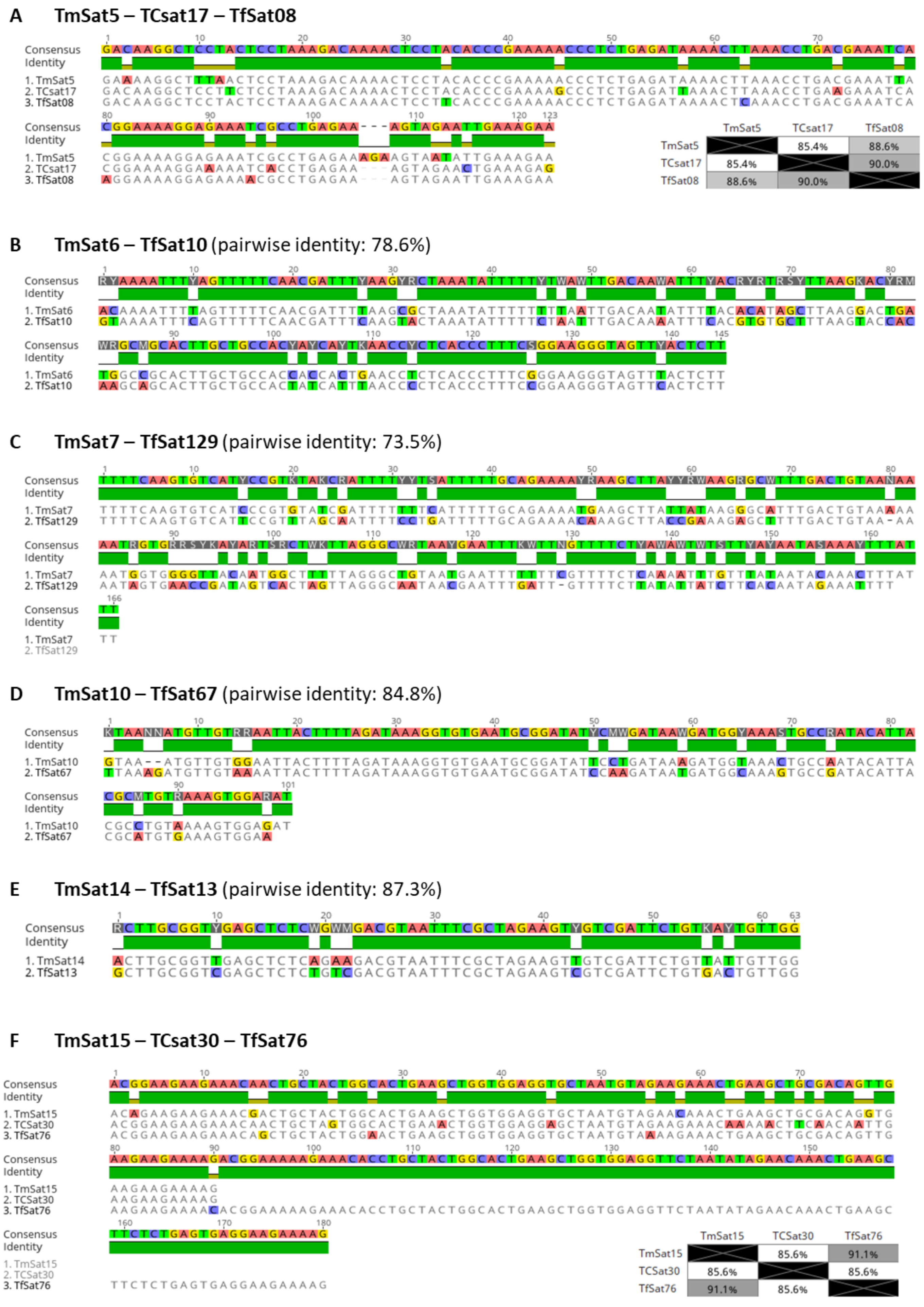

Fig. S20. Alignments of the *T. madens* low-copy-number satDNAs and their orthologs among the known *T. castaneum* and *T. freemani* satDNAs. Pairwise identities between the consensus sequences are indicated.

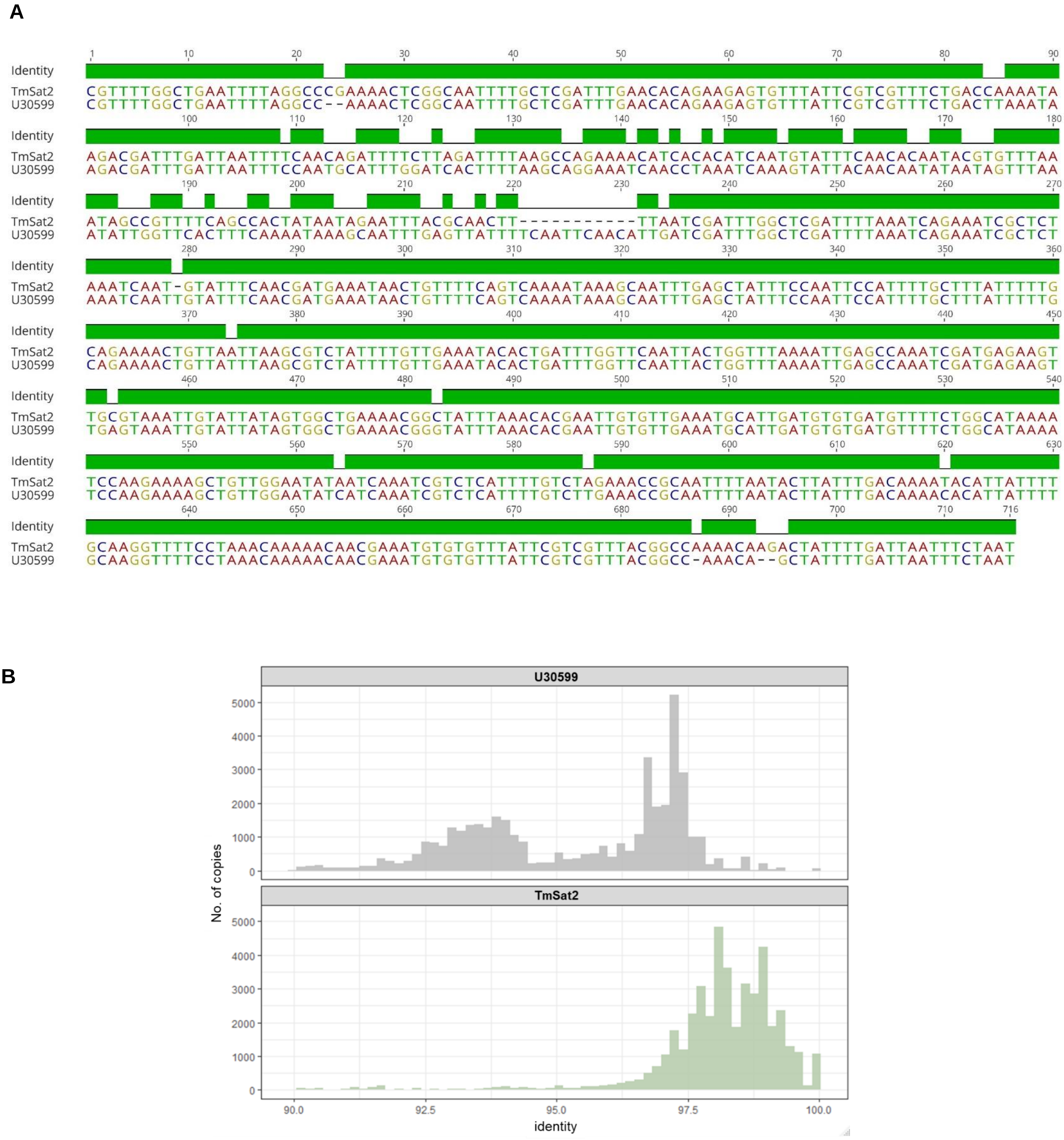

Fig. S21. Comparison of the TmSat2 consensus sequence defined in this work and the GenBank entry U30599 [23]. **(A)** The alignment of the consensus sequences, showing 90.4% of pairwise similarity. **(B)** Distribution of nucleotide sequence similarities between monomers annotated in the *T. madens* genome assembly Tmad1.0 and the sequence U30599 (upper panel), and the consensus sequence TmSat2 (bottom panel). The similarities above 90% are shown.
